# Antimalarial Imidazopyridines Incorporating an Intramolecular Hydrogen Bonding Motif: Medicinal Chemistry and Mechanistic Studies

**DOI:** 10.1021/acsinfecdis.2c00584

**Published:** 2023-03-22

**Authors:** Henrietta D. Attram, Constance M. Korkor, Dale Taylor, Mathew Njoroge, Kelly Chibale

**Affiliations:** † Department of Chemistry, University of Cape Town, Rondebosch 7701, South Africa; ‡ Drug Discovery and Development Centre (H3D), University of Cape Town, Rondebosch 7701, South Africa; § South African Medical Research Council Drug Discovery and Development Research Unit, University of Cape Town, Rondebosch 7701, South Africa; ∥ Institute of Infectious Disease and Molecular Medicine, University of Cape Town, Rondebosch 7701, South Africa

**Keywords:** intramolecular hydrogen bonding, imidazopyridines, *Plasmodium falciparum*, live-cell microscopy, heme fractionation

## Abstract

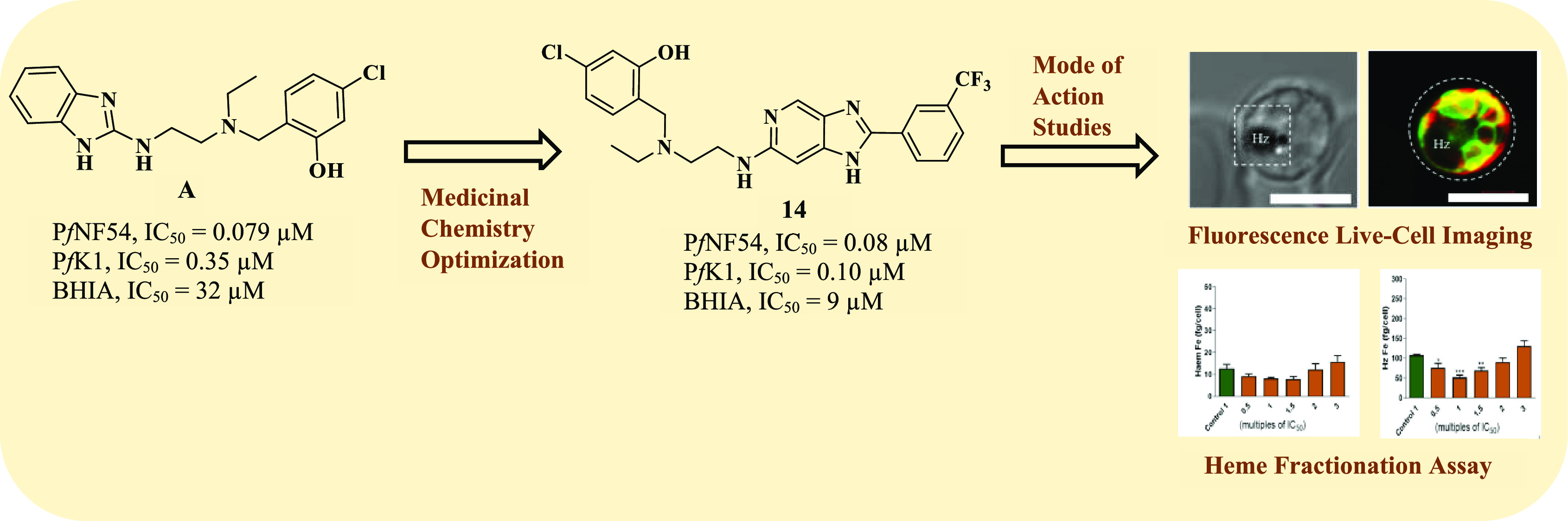

We previously identified a novel class of antimalarial benzimidazoles incorporating an intramolecular hydrogen bonding motif. The frontrunner of the series, analogue **A**, showed nanomolar activity against the chloroquine-sensitive NF54 and multi-drug-resistant K1 strains of *Plasmodium falciparum* (*Pf*NF54 IC_50_ = 0.079 μM; *Pf*K1 IC_50_ = 0.335 μM). Here, we describe a cell-based medicinal chemistry structure–activity relationship study using compound **A** as a basis. This effort led to the identification of novel antimalarial imidazopyridines with activities of <1 μM, favorable cytotoxicity profiles, and good physicochemical properties. Analogue **14** ( *Pf*NF54 IC_50_ = 0.08 μM; *Pf*K1 IC_50_ = 0.10 μM) was identified as the frontrunner of the series. Preliminary mode of action studies employing molecular docking, live-cell confocal microscopy, and a cellular heme fractionation assay revealed that **14** does not directly inhibit the conversion of heme to hemozoin, although it could be involved in other processes in the parasite’s digestive vacuole.

Malaria, an infectious disease caused by *Plasmodium* parasites, remains a significant cause of illness and malaise in the world’s population that is already plagued with other disease burdens and poverty. The World Health Organization’s African region has consistently accounted for more than 94% of the recorded cases, with pregnant women and children under five years old being the most affected.^1^ Despite the many successes in reducing the malaria burden between 2000 and 2020, the disease still accounted for 627,000 deaths, with an estimated 241 million cases in 2021. The outbreak of the COVID-19 pandemic and the restrictions related to its response interfered with malaria relief services and overwhelmed healthcare systems around the world, especially in low- and middle-income countries.^2^ This led to an increased number of cases compared to those reported in 2019.^1^ The effect of the pandemic on malaria prevention and treatment, coupled with the development of resistance to almost all available antimalarial chemotherapies, underscores the need to expand the antimalarial drug arsenal by exploring and developing new compound classes, with, among other attributes, a combination of novel modes of action, multistage activity, and no cross-resistance to existing clinically used drugs.

Intramolecular hydrogen bond formation has been shown to have tremendous effects on molecular structure and influences properties such as water solubility, lipophilicity, membrane permeability, pharmacokinetic and pharmacodynamic processes, as well as protein binding affinity, which ultimately translates to enhanced biological activity.^3−6^ Within the context of malaria, several compound classes incorporating an intramolecular hydrogen bond are known and include the clinically used amodiaquine, mefloquine, WR-194,965, JPC-2997, and JPC-3210 (Figure 1).^7−11^

**Figure 1:**
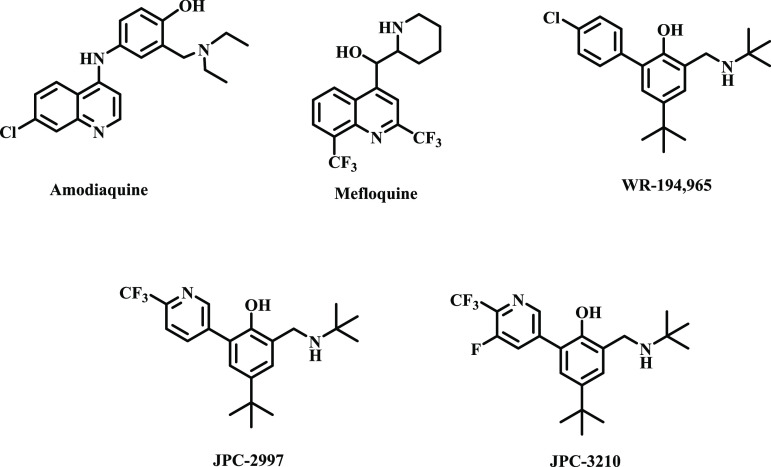
Antimalarials incorporating an intramolecular hydrogen bonding motif.

The benzimidazole and imidazopyridine scaffolds, on the other hand, are among some of the most privileged scaffolds in medicinal chemistry.^12,13^ This is due to their ability to interact selectively with diverse receptors, pathways, and enzymes, thereby yielding a variety of therapeutic effects. Previously, Nchinda and co-workers reported the development of fast-acting 2,6-disubstituted imidazopyridine compounds stemming from a phenotypic whole-cell high-throughput screening of a Soft-Focus Kinase library.^14^ Two compounds were identified from hit-to-lead drug discovery efforts with promising antiparasitic activities in vitro. This translated into an in vivo efficacy in *P. falciparum* NOD-*scid IL-2R*γ^*null*^ (NSG) mouse model.^14^

In a separate study, we synthesized and evaluated a novel class of benzimidazoles incorporating an intramolecular hydrogen-bonding motif. The compounds showed good antiplasmodium activities in vitro against the human malaria parasite and were generally non-cytotoxic.^15^ Based on the above precedents, we sought to further explore the medicinal chemistry of these chemical series by pursuing a scaffold-hopping approach wherein the benzimidazole core was replaced with an imidazopyridine core present in our previously described antimalarial agents.^14^ For this new series of compounds, the **R** substituents were carefully selected to partly introduce structural diversity around the imidazopyridine core and also reduce the lipophilicity of target compounds (Figure 2).

**Figure 2:**
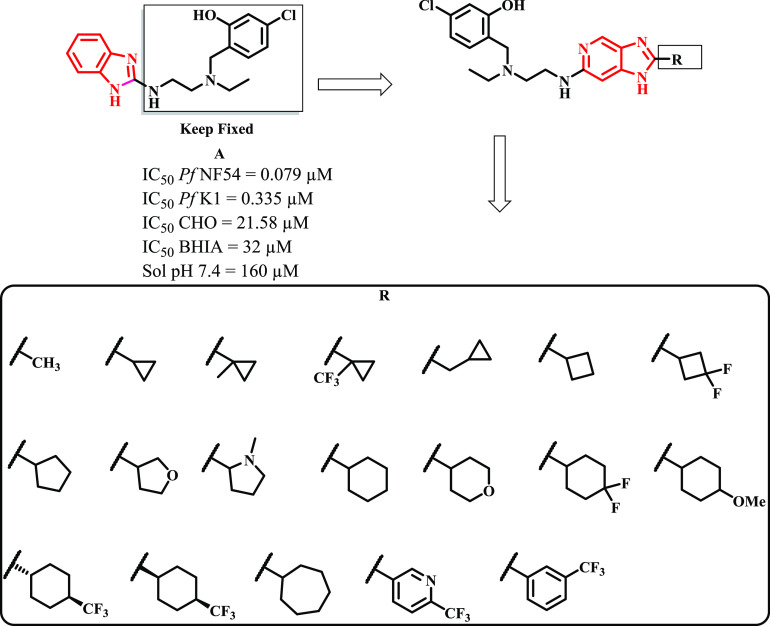
SAR plan implemented in this work. CHO: Chinese hamster ovarian cells; BHIA: β-hematin inhibition activity; sol: solubility.

To provide insight into the mechanism of action of this class of compounds, we employed a combination of molecular docking, live-cell confocal microscopy, and a cellular heme fractionation assay.

## Chemistry

**Scheme 1:**
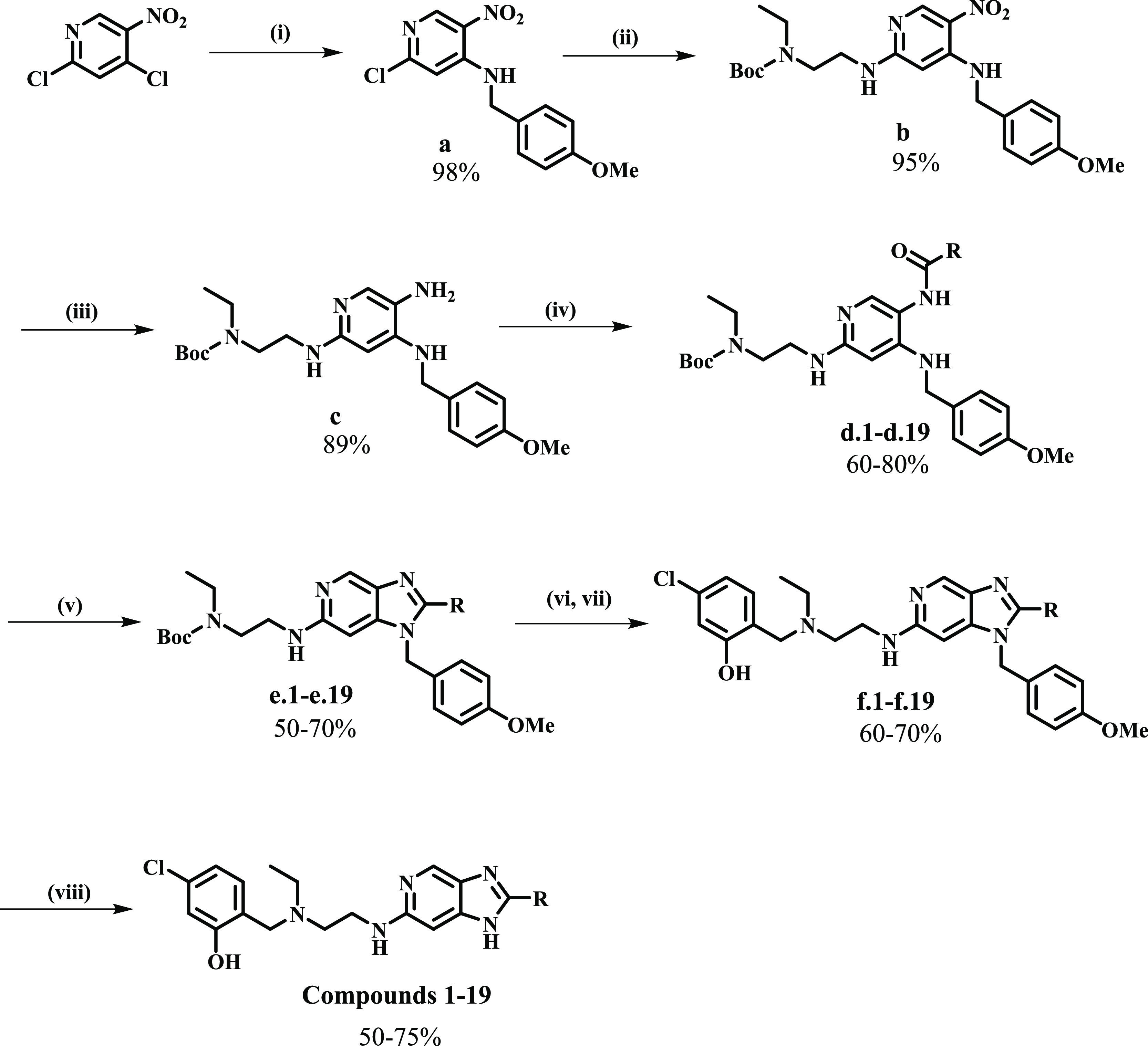
Synthetic Scheme for Imidazopyridine Analogues Reagents and conditions: (i) PMB-NH_2_ (1.8 eq), DIPEA (1.8 eq), THF, 0–25 °C, 30 min, 98%; (ii) *tert*-butyl (2-aminoethyl)(ethyl)carbamate (1.5 eq), Et_3_N (2 eq), DMF, μW 100 °C, 1 h, 60%; (iii) 10% Pd/C H_2_(g) 25 °C; (iv) R-COOH (1.3 eq) 85%, EDCI·HCl (1.5 eq), DMAP (0.1 eq), DCM, 25 °C, 12, 60–80%; (v) 2 M NaOH, EtOH, 80 °C, 12–72 h 50–70%; (vi, vii) 4 M HCl/dioxane 60–75%; (g) 4-chloro-2-hydroxybenzaldehyde, NaBH_4_, 25 °C, 24 h, 60–70%; (viii) TFA, 100 °C, 12 h, 50–75%.

Synthetic protocols reported in the literature were adapted to access the target compounds.^14^ The general protocol followed an eight-step synthetic route from commercially available 2,4-dichloro-5-nitropyridine (Scheme 1). A nucleophilic substitution was first performed to introduce a *para*-methoxybenzyl (PMB) amino-protecting group to form the 2-chloro-*N*-(4-methoxybenzyl)-5-nitropyridin-4-amine intermediate **a** in quantitative yield. This intermediate was subjected to a second nucleophilic substitution reaction under microwave irradiation with *tert*-butyl (2-aminoethyl)(ethyl)carbamate in the presence of triethylamine and *N*,*N*-dimethylformamide (DMF) to produce *tert*-butyl ethyl(2-((4-((4-methoxybenzyl)amino)-5-nitropyridin-2-yl)amino)ethyl)carbamate **b**.

The nitro group was then reduced using palladium on carbon (10% Pd/C) under H_2_ gas to deliver the corresponding aniline **c**. This intermediate was reacted with the appropriate carboxylic acids in the presence of 1-ethyl-3-(3-dimethylaminopropyl)carbodiimide, hydrochloride (EDCI·HCl), and a catalytic amount of 4-dimethylaminopyridine (DMAP) in dichloromethane (DCM) to produce the amide intermediates **d.1**–**d.19**. The imidazole ring was allowed to form at this stage by heating in 2 M aqueous NaOH and absolute ethanol at 80 °C to yield intermediates **e.1**–**e.19**. The appropriate cyclized intermediate was subjected to boc-deprotection followed by reductive amination with 4-chloro-2-hydroxybenzaldehyde in the presence of sodium borohydride to yield the penultimate intermediates **f.1**–**f.19**. Finally, the PMB group was removed using neat trifluoroacetic acid (TFA) to afford the desired imidazopyridine analogues **1–19** in moderate yields (50–75%).

**Scheme 2:**
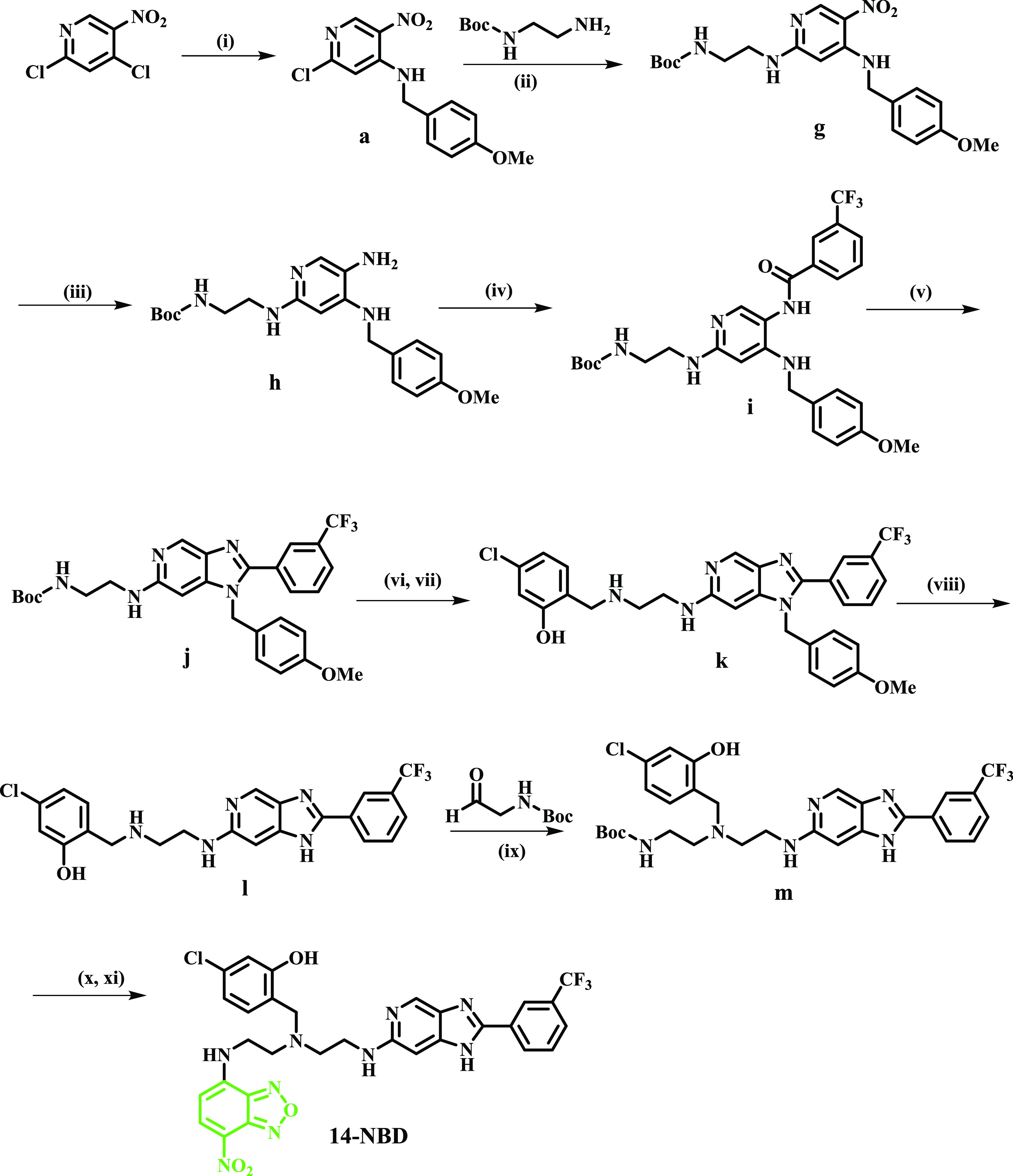
Synthetic Scheme for NBD-Labeled Compound **14** Reagents and conditions: (i) PMB-NH_2_ (1.8 eq), DIPEA (1.8 eq), THF, 0 °C to 25 °C, 30 min, quantitative; (ii) *N*-Boc ethylenediamine (1.5 eq), Et_3_N (2 eq), DMF, μW 90 °C, 1 h, 75%; (iii) Zn/AcOH, DCM, 25 °C, 30 min (iv) 3-trifluoromethyl benzoic acid (1.3 eq), EDCI·HCl (1.5 eq), DMAP (0.1 eq), DCM, 25 °C, 12 h; (v) 2 M NaOH, EtOH, 80 °C, 16 h, 65%; (vi) 4 N HCl/dioxane; (vii) 4-chloro-2-hydroxybenzaldehyde, NaBH_4_, 25 °C, 6 h, 65%; (viii) TFA, 100 °C, 12 h, 75%; (ix) NaBH_3_CN, cat. AcOH, MeOH, 80 °C, 16 h, 70%; (x) 4 N HCl/dioxane, 25 °C; (xi) NBD-chloride, NaHCO_3_, EtOAc, 60 °C, 24 h, 50%.

To probe the subcellular localization of the target compounds within the parasite, a representative fluorescent probe of one of the compounds was designed and synthesized by attaching 7-nitrobenz-2-oxa-1,3-diazole (NBD) on the ethylenediamine side chain through an ethyl linker, as illustrated in Scheme 2. Compound **14-NBD** was synthesized through an 11-step synthetic route from commercially available 2,4-dichloro-5-nitropyridine. A nucleophilic substitution reaction on the *para*-methoxybenzyl (PMB) amino-protecting group to form the 2-chloro-*N*-(4-methoxybenzyl)-5-nitropyridin-4-amine intermediate **a**, followed by a second nucleophilic substitution reaction involving the 2-chloro-*N*-(4-methoxybenzyl)-5-nitropyridin-4-amine intermediate with *N*-boc ethylenediamine in the presence of triethylamine and *N,N*-dimethylformamide (DMF), produced intermediate **g**. The nitro group was then reduced with Zn in acetic acid to deliver the corresponding aniline intermediate **h**. This intermediate was reacted with 3-trifluoromethyl benzoic acid in the presence of 1-ethyl-3-(3-dimethylaminopropyl)carbodiimide, hydrochloride (EDCI·HCl), and a catalytic amount of 4-dimethylaminopyridine (DMAP) in dichloromethane (DCM) to produce the amide intermediate **i**. At this stage, the imidazole ring was allowed to form by heating in 2 M aqueous NaOH and absolute ethanol at 80 °C to yield intermediate **j**. The cyclized intermediate was subjected to boc-deprotection followed by reductive amination with 4-chloro-2-hydroxybenzaldehyde in the presence of sodium borohydride to deliver the intermediate **k**. The PMB group was removed using neat trifluoroacetic acid (TFA), resulting in an intermediate **l**. Reductive amination of *N*-boc glycinal was then carried out in the presence of sodium cyanoborohydride to deliver the penultimate intermediate **m**. Finally, removal of the boc group, followed by a nucleophilic substitution reaction with NBD-chloride, yielded the target fluorescent probe **14-NBD**.

## Results and Discussion

### In Vitro Asexual Blood-Stage Antiplasmodium Activity and Cytotoxicity

All the compounds were evaluated for in vitro antiplasmodium activity against both the drug-sensitive NF54 and multidrug-resistant K1 strains of *P. falciparum*, and the SAR is discussed with respect to IC_50_ values on the NF54 strain. Aromatic groups bearing small non-polar *meta*- or *para*- electron-withdrawing substituents displayed better antiplasmodium potency, with compound **14** (IC_50_ = 0.08 μM) having the highest potency. Incorporation of heteroatoms in the saturated cyclic substituents as exemplified in compounds **2** (IC_50_ = 1.67 μM), **3** (IC_50_ = 2.37 μM) and **13** (IC_50_ = 1.03 μM) was detrimental to antiplasmodium activity. Further, electron-withdrawing substituents such as a fluoro (-F) or a trifluoromethyl (-CF_3_) on the cyclohexane ring led to comparable antiplasmodium activity in comparison to their unsubstituted congeners as shown in the matched pairs **9** (IC_50_ = 0.67 μM), **10** (IC_50_ = 0.86 μM) and **11** (IC_50_ = 0.69 μM), **12** (IC_50_ = 0.93 μM). However, the presence of the electron drawing -CF_3_ group on the cyclopropane was detrimental to activity while the -CH_3_ group was more tolerated compared to the unsubstituted analogue. Finally, changes in ring size did not have any significant effect on antiplasmodium activity as exemplified in compounds **5** (IC_50_ = 0.39 μM), **9** (IC_50_ = 0.67 μM), **1** (IC_50_ = 0.34 μM), **11** (IC_50_ = 0.69 μM), and **15** (IC_50_ = 0.67 μM).

The cytotoxicity of these analogues was determined against the Chinese hamster ovarian (CHO) cell line using the 3-(4,5-dimethylthiazol-2-yl)-2,5-diphenyltetrazolium bromide (MTT) assay. All the compounds showed a favorable cytotoxicity profile (Table 1) on account of their selectivity indices (SI > 10), with compound **14** (SI = 466.25) exhibiting the highest SI.

**Table 1:**
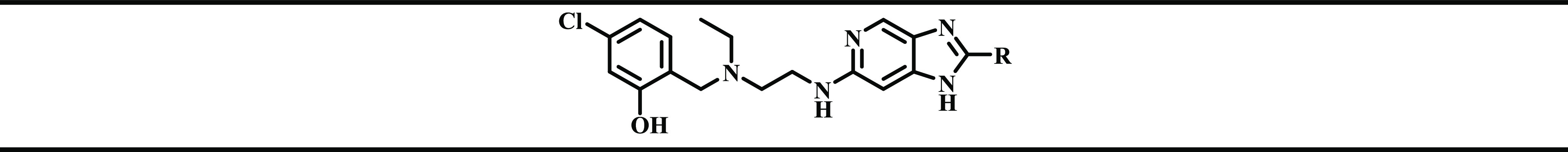

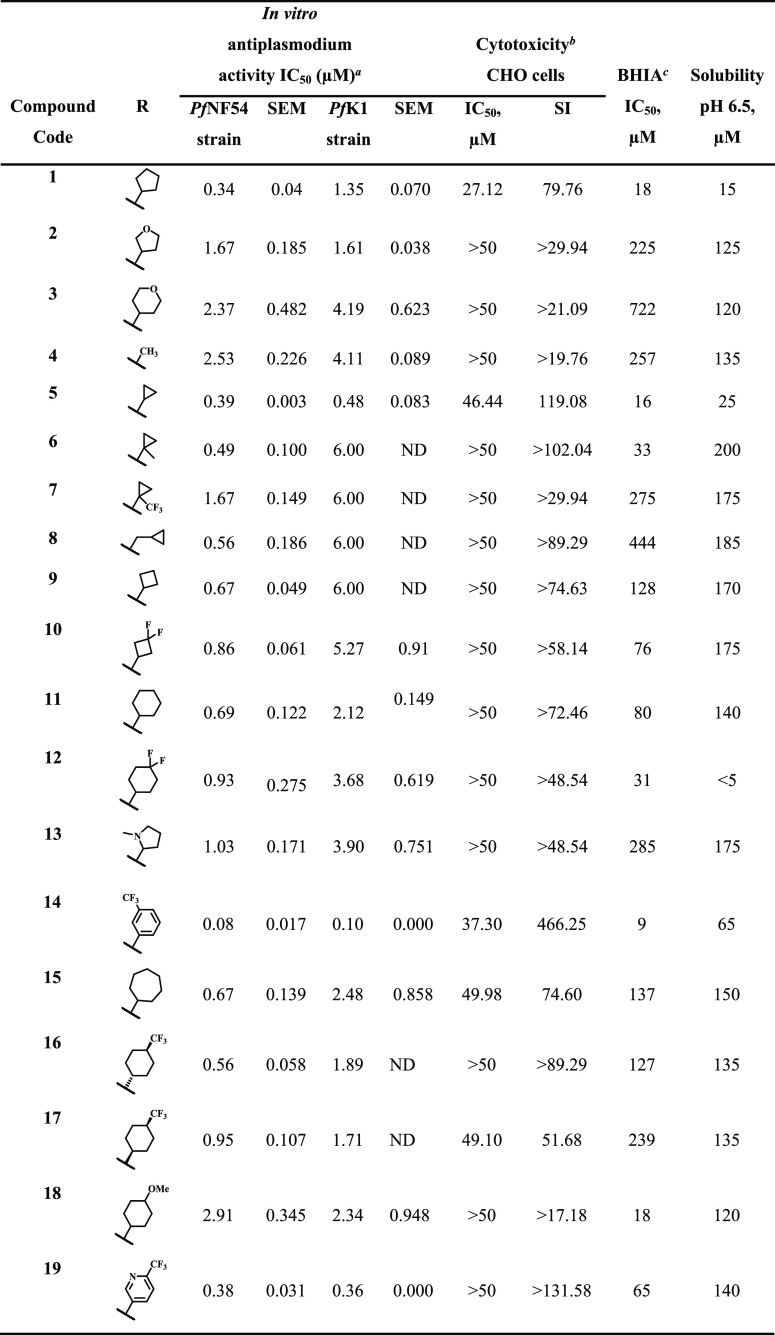
In Vitro Antiplasmodium Activity, Cytotoxicity, BHIA, and Solubility

a Determined from *n* ≥ 3 independent pLDH experiments with multidrug-resistant (K1) and chloroquine-sensitive (NF54) strains of *P. falciparum*. Controls: chloroquine (IC_50_ = 0.012 μM) and artesunate (IC_50_ = 0.007 μM).

b SI (selectivity index) = IC_50_ CHO/IC_50_*Pf*NF54.

c Mean from *n* = 3 independent experiments; controls: chloroquine (IC_50_ = 32 μM) and amodiaquine (IC_50_ = 5 μM).

### Metabolic Stability Studies of Selected Analogues

Selected imidazopyridine analogues exhibiting sub-micromolar in vitro asexual blood stage antiplasmodium activity (IC_50_ < 1 μM), suitable solubility (> 50 μM), and an acceptable selectivity profile relative to the mammalian Chinese hamster ovarian cell line (SI > 10) were evaluated for metabolic stability in mouse, rat, and human liver microsomes (Table 2). These compounds were generally not stable across all three species of microsomes, although **1** and **5** were more stable in human liver microsomes than in rodent microsomes. The metabolic stability of these analogues were assessed by the hepatic ratio (*E*_H_).

**Table 2:**
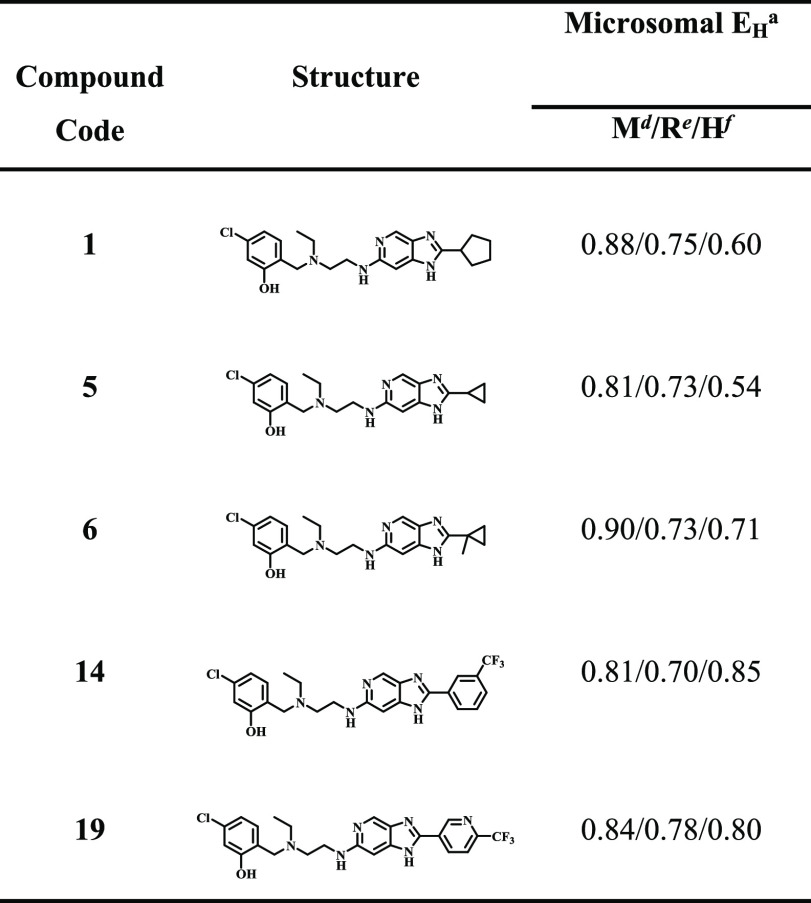
Microsomal Metabolic Stability of Selected Imidazopyridine Analogues

a *E*_H_, hepatic extraction ratio.

b M, mouse.

c R, rat.

d H, human.

### Metabolite Identification Studies for Compound **14**

**Figure 3:**
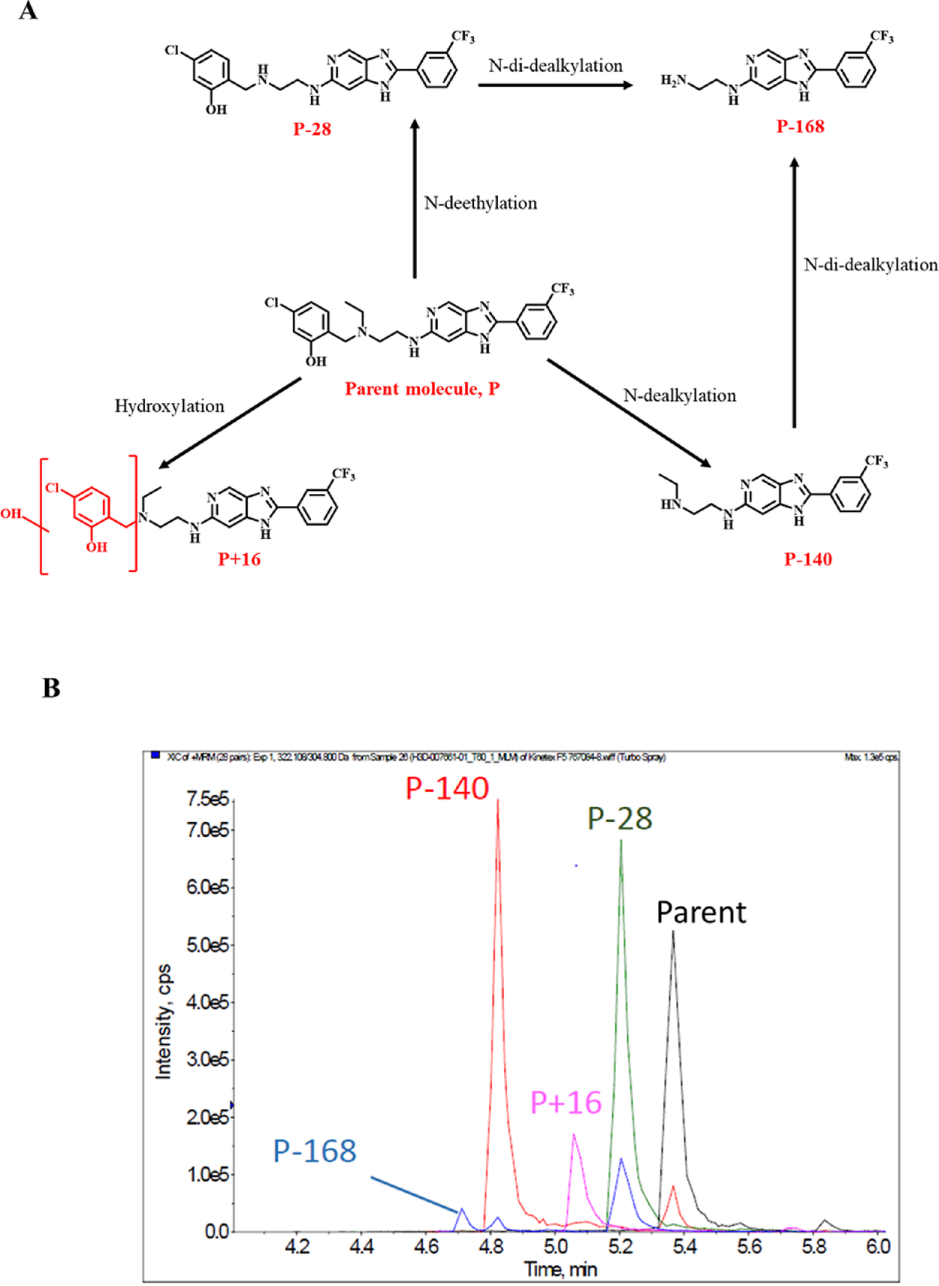
(A) Identified metabolites from the metabolism of compound **14** MLM; (B) XIC plot showing the relative abundance of the identified metabolites from compound **14**.

Considering the generally poor microsomal metabolic stability displayed by selected compounds, metabolite identification studies in mouse liver microsomes were undertaken on one of them, compound **14**. Four (4) metabolites were identified from the metabolism of **14** in mouse liver microsomes, with the primary metabolites (P-28 and P-140) arising from the dealkylation of the side chain *N*-alkyl groups (Figure 3) although the exact structure is yet to be confirmed. Notably, the formation of the metabolites required NADPH suggesting the presence of microsomes and the involvement of CYP450 enzymes, indicating that they were products of metabolism and not chemical degradation.

## Mechanistic Studies

### β-Hematin Inhibition Assay and Docking

Previously synthesized antimalarial imidazopyridines were shown to inhibit the formation of β-hematin in a cell-free assay and were subsequently confirmed as bonafide inhibitors of hemozoin formation in a cell-fractionation assay.^14,16^ Based on this precedence, the potential of our imidazopyridine series to inhibit hemozoin formation was assessed using the β-hematin inhibition Assay (BHIA) (Table 1). Using the discriminatory cut-off of <100 μM, only nine compounds: **1** (18 μM), **5** (16 μM), **6** (33 μM), **10** (76 μM), **11** (80 μM), **12** (31 μM), **14** (9 μM), **18** (18 μM), and **19** (65 μM) exhibited β-hematin inhibition activity in the preferred range, with compound **14** showing the highest potency.

**Figure 4:**
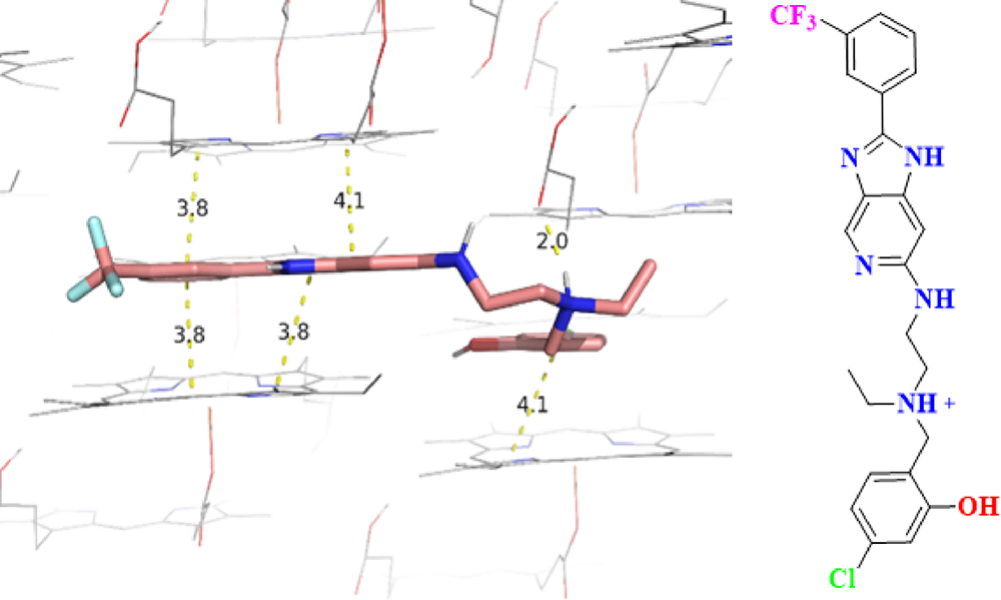
Predicted binding mode with the 001 face of β-hematin showing hydrogen-bonding interactions between the protonated tertiary amine and the propionate group of β-hematin at pH 4.5 (2.0 Å) and π–π-stacking interactions shown between the porphyrin ring of the heme and the 3-chlorophenol moiety (4.1 Å), 3-trifluoromethylphenyl moiety (3.8 Å), and imidazopyridine core (4.1 Å).

The frontrunner compound **14**, which exhibited sub-micromolar in vitro asexual blood-stage antiplasmodium activity, acceptable cytotoxicity profile against the mammalian CHO cell line, good solubility, and potent β-hematin inhibition activity, also showed specific intermolecular interactions with the previously published crystal surface of β-hematin.^17^ The imidazopyridine core, the 3-trifluoromethylphenyl, and the 3-chlorophenol moieties of the compound interact through π–π stacking with the porphyrin ring of β-hematin. On the other hand, the basic nitrogen of the tertiary amine on the side chain of the compound forms a hydrogen bond with the propionate group of β-hematin when protonated at pH 4.5 (Figure 4), further supporting the inhibition of β-hematin as a possible contributing mode of action.

### Fluorescence Drug Localization Studies

**Figure 5:**
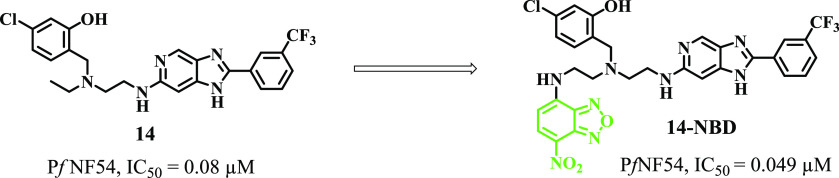
Depiction of a novel two-chain fluorescent derivative of compound **14**.

Fluorescence drug-localization studies were employed as the starting point to probe the subcellular localization of the target compounds within the parasite. The representative compound, **14** (IC_50_*Pf*NF54 = 0.08 μM), was first assessed for its inherent fluorescence for imaging in *P. falciparum* using a fluorimeter. Excitation between 200 and 600 nm yielded no significant emission with reference to the blank solvent. This underscored the need to attach an external fluorophore with suitable photophysical properties and comparable in vitro antiplasmodium activity to the parent compound. 7-Nitrobenz-2-oxa-1,3-diazole (NBD) was selected as an appropriate extrinsic fluorophore based on its small size, commercial availability, and stability over a biologically relevant pH range.^18^ The point of attachment of the fluorophore was guided by the earlier SAR studies on the scaffold. The NBD-labeled probe retained nanomolar in vitro activity against *P. falciparum* (**14-NBD***Pf*NF54 IC_50_ = 0.049 μM; Figure 5). It also possesses photophysical properties that are suitable for live-cell imaging.

Subcellular accumulation of *Pf*-infected red blood cells was assessed through confocal microscopy. Commercially available organelle trackers such as the LysoTracker Red, MitoTracker Deep Red, ER-Tacker Red, DRAQ5, and Nile Red aided the colocalization studies of **14-NBD**. These dyes illuminate the acidic organelles such as the parasite’s digestive vacuole, mitochondrion, endoplasmic reticulum, nucleus, and lipids, respectively.^19−22^

**Figure 6:**
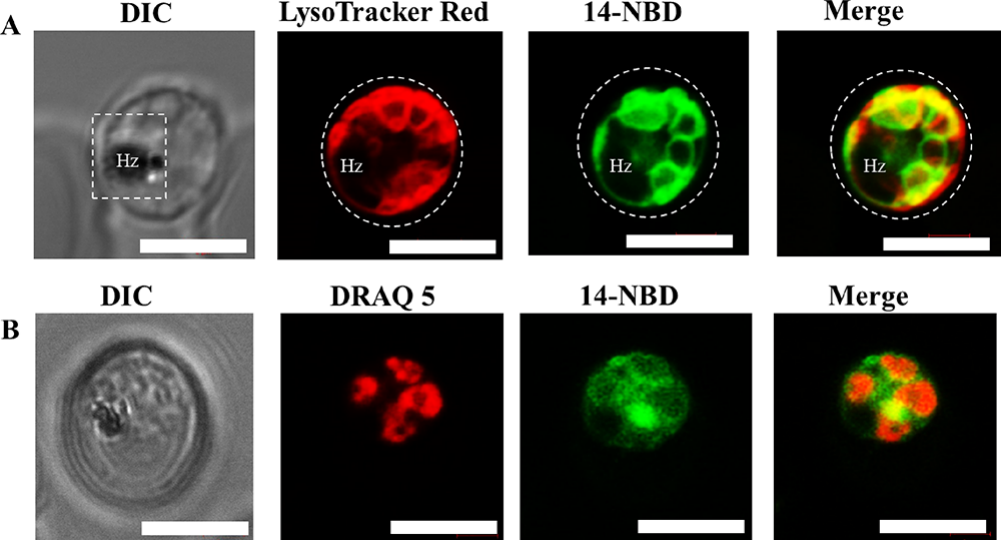
Live-cell confocal microscopy of a *P. falciparum*-infected erythrocyte treated with **14-NBD** with LysoTracker Red (red, panel A) and nuclear marker DRAQ5 (red, panel B). Regions of complete overlap are shown in the merged image (yellow). Scale bars: 5 micrometers (μm). Hz: hemozoin; DIC: differential interference contrast.

The results from the live-cell confocal microscopy showed a partial accumulation between **14-NBD** and LysoTracker Red, with regions of intense localization observed around the parasite’s membrane structures. No significant accumulation was seen in the areas around the hemozoin crystals (Hz), suggesting that **14-NBD** does not localize in the parasite’s digestive vacuoles (Figure 6A). It is noteworthy that while **14-NBD** retained antiplasmodium activities compared to the parent compound, the presence of the NBD fluorophore can influence the accumulation of the compound in the parasite. Similarly, no colocalization was observed between the nuclear marker, DRAQ5, and **14-NBD**, thereby eliminating the parasite’s nucleus as a site of action of the compound.

**Figure 7:**
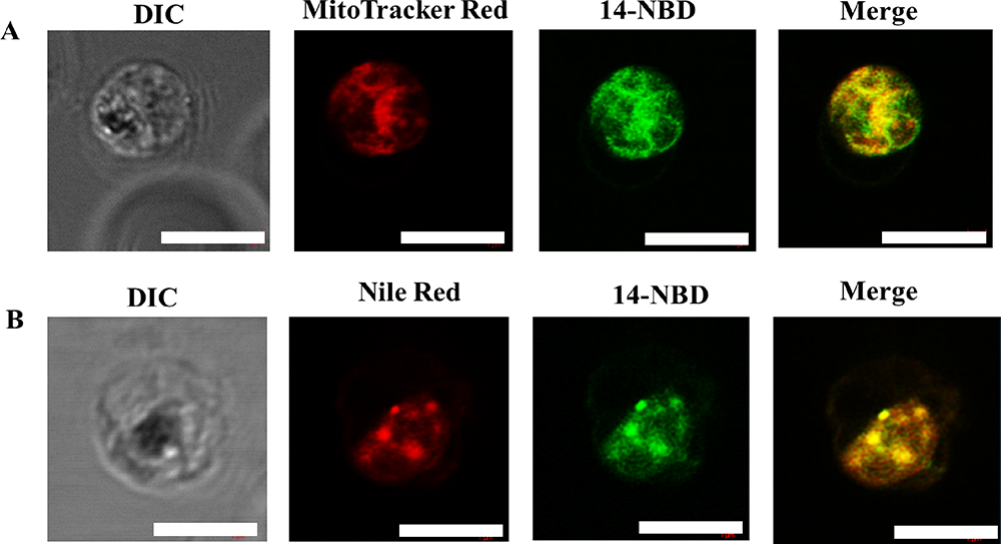
Live-cell confocal microscopy of *P. falciparum*-infected erythrocyte treated with **14-NBD** with MitoTracker Deep Red (red, panel A) and Nile Red (red, panel B). Regions of complete overlap are shown in the merged image (yellow). Scale bars: 5 micrometers (μm). Hz: hemozoin; DIC: differential interference contrast.

Although the biochemistry of hemozoin formation has not been fully elucidated, with many hypotheses in the literature regarding its formation,^23−26^ one hypothesis that has gained popularity is that it is lipid-catalyzed. Neutral lipids, in particular, have been associated with hemozoin formation.^27^ Consequently, Nile Red was co-incubated with **14-NBD** to identify and assess the interaction of **14-NBD** with neutral lipids. Punctuate structures believed to be neutral lipid droplets were observed close to the hemozoin crystals. They are formed from the parasite’s cytosol and transported into its food vacuole, where they aid in the conversion of heme to hemozoin. **14-NBD** colocalized with Nile Red, indicating the compound’s association with neutral lipid droplets (Figure 7B). Furthermore, **14-NBD** interacted significantly with the parasite’s mitochondrion. This is shown by the colocalization between **14-NBD** and MitoTracker Deep Red (Figure 7A).

### Heme Speciation Assay

**Figure 8:**
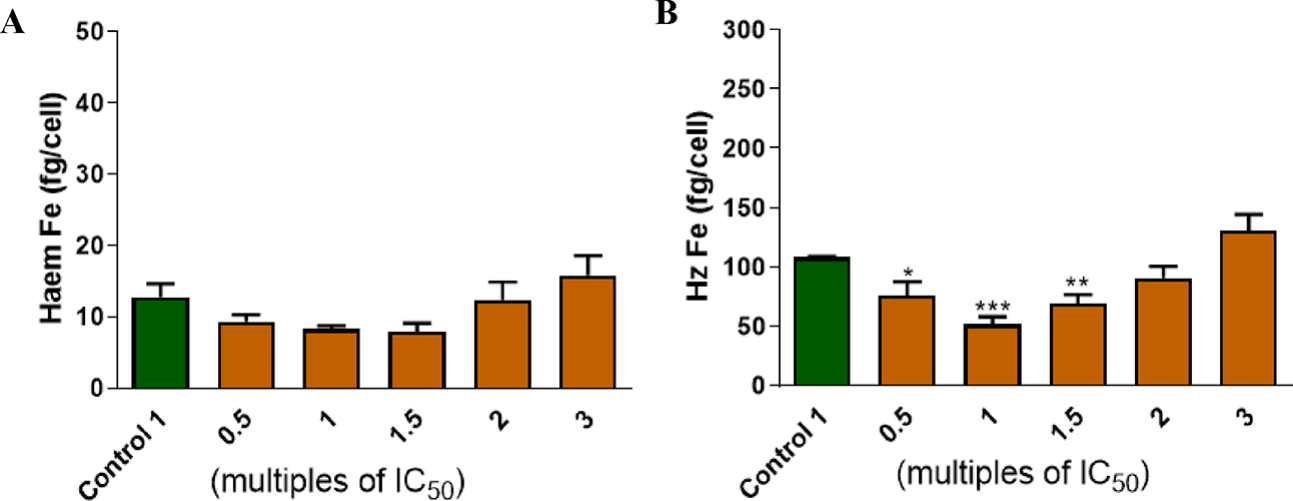
Dose-dependent heme fractionation profiles of compound **14** and amount of “free” heme Fe (A) and hemozoin (Hz) Fe (B) at increasing concentrations of the compounds.

To augment the findings from live-cell confocal microscopy, β-hematin inhibition assay, and docking studies that suggest hemozoin inhibition as a possible mode of action of this class of compounds, the frontrunner of the series, **14** was tested in a cellular heme fractionation assay to evaluate the dose-dependent effect of the compound on various iron species in the parasite and to confirm the compound’s ability to inhibit intracellular Hz formation in *P. falciparum* parasites according to methods previously described by Combrinck and co-workers.^28^ However, at increasing concentrations of **14**, no statistically significant change was observed in the levels of heme. Conversely, a significant decrease in the levels of hemozoin was observed between 0.5–2× IC_50_ of **14** (Figure 8), suggesting that although compound **14** does not directly interfere with the conversion of heme to hemozoin, it could be targeting other processes in the parasite’s digestive vacuole. At this juncture, it is noteworthy that a true hemozoin inhibitor causes a dose-dependent increase in “free” heme and a corresponding decrease in hemozoin.

## Conclusions

With the goal of incorporating an intramolecular hydrogen bonding motif in known antimalarial chemotypes, we identified a set of potent antimalarial imidazopyridine analogues. The medicinal chemistry of the chemical series with respect to antiplasmodium SAR profiles around an earlier-identified benzimidazole core was explored, leading to the identification of the series’ frontrunner **14,** which displayed the highest potency within the series. Furthermore, all compounds from this series showed a favorable cytotoxicity profile against the CHO cell line. Nonetheless, these compounds were metabolically labile and could not be progressed to in vivo efficacy studies. However, metabolite identification studies provided insight into the metabolic hotspots, which can be used to synthesize analogues that would address this liability in future studies. Although **14** interacted favorably with the β-hematin surface through docking and showed potent β-hematin inhibition, no statistically significant effect on the levels of heme was observed after a dose-dependent treatment of *P. falciparum* cells with **14**. Conversely, hemozoin levels decreased with increasing concentrations of **14**. Hence, we hypothesized that while **14** does not directly affect the conversion of heme to hemozoin, it may target different digestive vacuole processes. The interaction of **14-NBD** with other organelles aside from the parasite’s digestive vacuole may suggest the potential involvement of a novel target.

## Methods

All commercially available chemicals were purchased from either Sigma-Aldrich (Germany) or Combi-Blocks (United States). ^1^H NMR (all intermediates and final compounds) and ^13^C NMR (for target compounds only) spectra were recorded on a Bruker Spectrometer at 300, 400, or 600 megahertz (MHz). Melting points for all target compounds were determined using a Reichert-Jung Thermovar hot-stage microscope coupled to a Reichert-Jung Thermovar digital thermometer (20–350 °C range). Reaction monitoring using analytical thin-layer chromatography (TLC) was performed on aluminum-backed silica-gel 60 F_254_ (70–230 mesh) plates with detection and visualization done using (a) UV lamp (254/366 nm), (b) iodine vapors, or (c) ninhydrin spray reagent. Column chromatography was performed with Merck silica-gel 60 (70–230 mesh). Chemical shifts (δ) are reported in ppm downfield from trimethlysilane (TMS) as the internal standard. Coupling constants (*J*) were recorded in Hertz (Hz). Purity of compounds was determined by an Agilent 1260 Infinity binary pump, Agilent 1260 Infinity diode array detector, Agilent 1290 Infinity column compartment, Agilent 1260 Infinity standard autosampler, and Agilent 6120 quadrupole (single) mass spectrometer, equipped with APCI and ESI multi-mode ionization source. All compounds tested for biological activity were confirmed to have ≥95% purity by HPLC. Solubility, biological assays, and any experimental data not shown below (i.e., NMR of compound intermediates) are fully supplied and detailed in the Supporting information.

### Preparation of 2-Chloro-*N*-(4-methoxybenzyl)-5-nitropyridin-4-amine (**a**)

A mixture of *p*-methoxybenzyl amine (2.56 g, 18.65 mmol) and *N*,*N*-diisopropylethylamine (DIPEA) in tetrahydrofuran (THF) was added dropwise to a 0 °C solution of 2,4-dichloro-5-nitropyridine (2.00 g, 10.36 mmol) in THF. The solution was then warmed up to 25 °C and stirred for an additional 30 min. Water was then added, and the resulting mixture was extracted with ethyl acetate. The combined organic layer was dried over anhydrous Na_2_SO_4_ and concentrated under reduced pressure to produce the desired intermediate as a yellow solid in compound in 98% yield. ^1^H-NMR (600 MHz, DMSO-*d*_6_): δ 8.96 (t, *J* = 6.1 Hz, 1H), 8.85 (s, 1H), 7.29 (d, *J* = 8.7 Hz, 2H), 6.94 (s, 1H), 6.89 (d, *J* = 8.7 Hz, 2H), 4.57 (d, *J* = 6.1 Hz, 2H), 3.71 (s, 3H). ^13^C-NMR (151 MHz, DMSO): δ 159.91, 155.92, 150.57, 150.08, 130.29, 129.76 (2C), 115.42 (2C), 108.83, 56.41, 46.26. HPLC-MS (ESI): purity = 98%, *t*_R_ = 2.457 min, *m/z* [M-H]^+^ = 294.0.

### Preparation of *tert*-Butylethyl(2-((4-((4-methoxybenzyl)amino)-5-itropyridin-2-yl)amino)ethyl)carbamate (**b**)

A mixture of 2-chloro-*N*-(4-methoxybenzyl)-5-nitropyridin-4-amine (**1a**) (5.00 g, 17.02 mmol), *tert*-butyl (2-aminoethyl)(ethyl)carbamate (4.81 g, 25.53 mmol), and triethylamine was made in *N*,*N*-dimethylformamide (DMF). The mixture was heated under microwave radiation at 100 °C for 1 h. When the reaction had completed, water was added, and the mixture was extracted with ethyl acetate (4 × 30 mL). The combined organic layer was dried over anhydrous Na_2_SO_4_, concentrated under reduced pressure, and purified via column chromatography. A yellow solid was obtained as the product. ^1^H-NMR (600 MHz, chloroform-*d*): δ 8.90 (s, 1H), 8.36 (s, 1H) 7.22 (d, *J* = 8.7 Hz, 2H), 6.85 (d, *J* = 8.7 Hz, 2H), 4.37 (s, 2H), 3.76 (s, 3H), 3.37–3.34 (m, 4H), 3.17 (q, *J* = 7.1 Hz, 2H), 1.41 (s, 9H), 1.05 (t, *J* = 7.1 Hz, 3H). ^13^C-NMR (151 MHz, CDCl_3_): δ 161.22, 159.20, 156.45, 150.82, 149.91, 128.71, 128.49 (2C), 124.63, 114.31(2C), 83.65, 79.91, 55.26, 46.20, 45.70, 43.02, 41.73, 28.37 (3C), 13.87. HPLC-MS (ESI): purity = 99%, *t*_R_ = 2.641 min, *m/z* [M + H]^+^ = 446.2.

### Preparation of *tert*-Butylethyl(2-((4-((4-methoxybenzyl)amino)-5-nitropyridin-2-yl)amino)ethyl)carbamate (**c**)

A mixture of *tert*-butyl ethyl(2-((4-((4-methoxybenzyl)amino)-5-nitropyridin-2 yl)amino)ethyl)carbamate (**b**) (6.00 g, 13.47 mmol) and 10% Pd/C in methanol was stirred for 16 h at 25 °C under hydrogen gas. After the reaction had been completed, the mixture was filtered through a pad of Celite and concentrated in vacuo to obtain the product, which was used in the next reaction without any further purification. ^1^H-NMR (600 MHz, chloroform-*d*): δ 7.42 (s, 1H), 7.24 (d, *J* = 8.7 Hz, 2H), 6.84 (d, *J* = 8.7 Hz, 2H), 4.96 (s, 1H), 4.23 (s, 2H), 3.77 (s, 3H ), 3.32–3.32 (m, 4H), 3.18 (q, *J* = 7.1 Hz, 2H), 1.41 (s, 9H), 1.04 (t, *J* = 7.0 Hz, 3H). ^13^C-NMR (151 MHz, CDCl_3_): δ 158.99, 155.78, 149.34, 130.21, 129.99, 128.75 (2C), 119.97, 114.10 (2C), 87.26, 79.42, 55.29, 55.23, 46.61, 46.21, 41.54, 29.65, 28.42 (3C), 13.86. HPLC-MS (ESI): purity = 97%, *t*_R_ = 2.334 min, *m/z* [M + H]^+^ = 416.2.

### General Procedure for the Synthesis of Intermediates **d** and **e**

#### Amide Coupling (Intermediate **d**)

Intermediate **c** (1 eq) was dissolved in DCM with the appropriate carboxylic acid (1.3 eq) and 4-dimethylaminopyridine (DMAP, 0.1 eq). 1-Ethyl-3-(3-dimethylaminopropyl)carbodiimide hydrochloride (EDCI·HCl, 1.5 eq) was then added, and the reaction mixture was stirred at 25 °C for 16 h. Water was added, and the solution was extracted with ethyl acetate, dried over anhydrous Na_2_SO_4_, and concentrated under reduced pressure. The residue was used in the subsequent reaction without any further purification.

#### Cyclization (Intermediates **e.1**–**e.19**)

The corresponding amide intermediate **d** was dissolved in ethanol (10 mL), and 2 M NaOH solution (10 mL) was added. The resulting mixture was heated at 80 °C for 24–72 h depending on the amide intermediate. When the reaction had gone to completion, the solvent was removed in vacuo, and saturated citric acid was added to the residue. Extraction was done with DCM (2 × 20 mL), and the combined organic extract was dried over anhydrous Na_2_SO_4_, filtered, and concentrated in vacuo. The residue was purified via column chromatography (DCM/MeOH) to obtain the corresponding product.

##### *tert*-Butyl(2-((2-cyclopentyl-1-(4-methoxybenzyl)-1*H*-imidazo[4,5-*c*]pyridin-6-yl)amino)ethyl)(ethyl)carbamate (**e.1**)

Obtained from intermediate **c** (500 mg, 1.20 mmol) and cyclopentane carboxylic acid (0.16 mL, 1.56 mmol) as a wine-colored sticky solid (46%, 270.4 mg); Rf (DCM: MeOH, 9:1) 0.64; ^1^H-NMR (600 MHz, chloroform-*d*): δ 8.37 (s, 1H), 6.97 (d, *J* = 8.7 Hz, 2H), 6.80 (d, *J* = 8.7 Hz, 2H), 6.47 (s, 1H), 5.18 (s, 2H), 3.74 (s, 3H), 3.36–3.10 (m, 6H), 2.71 (p, *J* = 8.1 Hz, 1H), 2.05–1.92 (m, 2H), 1.89–1.78 (m, 3H), 1.71–1.51 (m, 3H), 1.39 (s, 9H), 1.03 (t, *J* = 7.1 Hz, 3H). ^13^C-NMR (151 MHz, CDCl_3_): δ 181.48, 159.28, 154.15, 127.56, 127.42 (2C), 114.35 (2C), 85.09, 79.49, 55.24, 46.30, 45.90, 44.64, 43.14, 41.82, 37.16, 32.08 (2C), 30.17, 28.39 (3C), 25.86 (2C), 25.76 (2C), 13.93. HPLC-MS (ESI): purity = 98%, *t*_R_ = 2.598 min, *m/z* [M + H]^+^ = 494.3.

##### *tert*-Butylethyl(2-((1-(4-methoxybenzyl)-2-(tetrahydrofuran-3-yl)-1*H*-imidazo[4,5-*c*]pyridin-6-yl)amino)ethyl)carbamate (**e.2**)

Obtained from intermediate **c** (500 mg, 1.20 mmol) and tetrahydrofuran-3-carboxylic acid (0.14 mL, 1.56 mmol) as a wine-colored sticky solid (68%, 401.7 mg); Rf (DCM: MeOH, 9:1) 0.57; ^1^H-NMR (600 MHz, chloroform-*d*): δ 8.45 (s, 1H), 6.96 (d, *J* = 8.7 Hz, 2H), 6.81 (d, *J* = 8.7 Hz, 2H), 6.45 (s, 1H), 5.17 (s, 2H), 4.04–3.98 (m, 2H), 3.93–3.86 (m, 2H), 3.74 (s, 3H), 3.50–3.44 (m, 1H), 3.39–3.33 (m, 4H), 3.20 (q, *J* = 7.2 Hz, 2H), 2.37–2.30 (m, 1H), 2.24–2.16 (m, 1H), 1.40 (s, 9H), 1.05 (t, *J* = 7.1 Hz, 3H). ^13^C-NMR (151 MHz, CDCl_3_): δ 159.43, 156.14, 154.68, 137.64, 133.55, 127.48, 127.27 (2C), 114.48 (2C), 84.86, 79.53, 71.93, 70.92, 68.33, 55.27, 46.34, 45.97, 43.07, 41.98, 37.14, 31.86, 29.65, 28.40 (3C), 13.89. HPLC-MS (ESI): purity = 98%, *t*_R_ = 2.448 min, *m/z* [M + H]^+^ = 496.3.

##### *tert*-Butylethyl(2-((1-(4-methoxybenzyl)-2-(tetrahydro-2*H*-pyran-4-yl)-1*H*-imidazo[4,5-*c*]pyridin-6-yl)amino)ethyl)carbamate (**e.3**)

Obtained from intermediate **c** (500 mg, 1.20 mmol) and tetrahydro-2*H*-pyran-4-carboxylic acid (202.96 mg, 1.56 mmol) as a wine-colored sticky solid (98%, 599.12 mg); Rf (DCM: MeOH, 9:1) 0.56; ^1^H-NMR (600 MHz, chloroform-*d*): δ 8.44 (s, 1H), 6.93 (d, *J* = 8.7 Hz, 2H), 6.78 (d, *J* = 8.7 Hz, 2H), 6.34 (s, 1H, ), 5.15 (s, 2H), 4.02–3.96 (m, 2H), 3.71 (s, 3H), 3.42–3.36 (m, 2H), 3.34–3.30 (m, 4H), 3.16 (q, *J* = 7.3 Hz, 2H), 2.93 (tt, *J* = 11.5, 3.7 Hz, 1H), 2.08–1.99 (m, 2H), 1.68–1.63 (m, 2H), 1.37 (s, 9H), 1.01 (t, *J* = 7.1 Hz, 3H). ^13^C-NMR (151 MHz, CDCl_3_): δ 159.34, 157.79, 154.73, 127.39 (2C), 114.43 (2C), 84.91, 79.47, 67.50, 67.45 (2C), 55.24, 46.23, 45.96, 42.99, 41.99, 33.78, 31.26, 31.21 (2C), 29.62, 29.26, 28.47, 28.38 (3C), 13.86. HPLC-MS (ESI): purity = 97%, *t*_R_ = 2.457 min, *m/z* [M + H]^+^ = 510.2.

### General Procedure for the Synthesis of Intermediates **f.1**–**f.19**

#### Boc-Deprotection

The appropriate intermediate **e.1**–**e.19** was dissolved in 4 M HCl/dioxane, and the mixture was stirred at 25 °C for 2 h. When the reaction was complete, the solvent was removed in vacuo, and the residue was neutralized with Amberlyst A21 in a mixture of DCM and methanol. Amberlyst was filtered off, the solvent was removed in vacuo, and the residue was used in the next reaction without further purification.

#### Reductive Amination

The crude product from step (a) above and 4-chloro-2-hydroxybenzaldehyde in methanol was stirred at 25 °C for 6 h. The mixture was cooled at 0 °C, and sodium borohydride (NaBH_4_) was added portion-wise. After the addition, the reaction was allowed to warm to room temperature (25 °C) for 2 h. The solvent was removed in vacuo, and the residue was diluted with deionized water. The compound was extracted with DCM and dried over anhydrous sodium sulfate. The solvent was removed in vacuo, and the residue was purified via column chromatography to obtain the desired product.

##### 5-Chloro-2-(((2-((2-cyclopentyl-1-(4-methoxybenzyl)-1*H*-imidazo[4,5-*c*]pyridin-6-yl)amino)ethyl)(ethyl)amino)methyl)phenol (**f.1**)

Obtained from intermediate **e.1** (160 mg, 0.41 mmol) and 4-chloro-2-hydroxybenzaldehyde (76 mg, 0.49 mmol) as a pale yellow sticky solid (53%, 116 mg); Rf (DCM:MeOH, 9:1) 0.50; ^1^H-NMR (600 MHz, chloroform-*d*): δ 8.44 (d, *J* = 1.0 Hz, 1H), 6.93 (d, *J* = 8.7 Hz, 2H), 6.82–6.80 (m, 3H), 6.73 (d, *J* = 2.1 Hz, 1H), 6.67 (dd, *J* = 8.0, 2.1 Hz, 1H), 6.03 (d, *J* = 1.0 Hz, 1H), 5.13 (s, 2H), 3.74 (s, 3H), 3.72 (s, 2H), 3.38 (t, *J* = 6.5 Hz, 2H), 3.14–3.08 (m, 1H), 2.72 (t, *J* = 6.5 Hz, 2H), 2.61 (q, *J* = 7.2 Hz, 2H), 1.98–1.92 (m, 4H, 1.86–1.79 (m, 2H), 1.64–1.57 (m, 2H), 1.03 (t, *J* = 7.2 Hz, 3H). ^13^C-NMR (151 MHz, CDCl_3_): δ 159.80, 159.29, 158.80, 154.09, 143.91, 138.46, 134.01, 133.98, 129.25, 128.58, 127.48, 127.39 (2C), 119.13, 116.42, 114.40 (2C), 85.32, 57.12, 55.27, 52.13, 47.60, 46.25, 40.48, 37.14, 32.10 (2C), 25.75 (2C), 10.91. HPLC-MS (ESI): purity = 98%, *t*_R_ = 2.450 min, *m/z* [M + H]^+^ = 534.2.

##### 5-Chloro-2-((ethyl(2-((1-(4-methoxybenzyl)-2-(tetrahydrofuran-3-yl)-1*H*-imidazo[4,5-*c*]pyridin-6-yl)amino)ethyl)amino)methyl)phenol (**f.2**)

Obtained from intermediate **e.2** (140 mg, 0.35 mmol) and 4-chloro-2-hydroxybenzaldehyde (66 mg, 0.42 mmol) as an orange-colored sticky solid (60%, 113 mg); Rf (DCM:MeOH, 9:1) 0.53; ^1^H-NMR (600 MHz, chloroform-*d*): δ 8.48 (d, *J* = 1.0 Hz, 1H), 6.93 (d, *J* = 8.7 Hz, 2H), 6.82–6.80 (m, 3H), 6.72 (d, *J* = 2.1 Hz, 1H), 6.66 (dd, *J* = 8.0, 2.1 Hz, 1H), 6.08 (d, *J* = 1.0 Hz, 1H), 5.13 (s, 2H), 4.02–3.95 (m, 3H), 3.93–3.84 (m, 2H), 3.74 (s, 3H), 3.74 (s, 2H), 3.42 (t, *J* = 6.5 Hz, 2H), 2.75 (t, *J* = 6.5 Hz, 2H), 2.64 (q, *J* = 7.2 Hz, 2H), 2.35–2.26 (m, 1H), 2.23–2.13 (m, 1H), 1.05 (t, *J* = 7.1 Hz, 3H). ^13^C-NMR (151 MHz, CDCl_3_): δ 159.44, 158.78, 155.83, 154.36, 143.95, 139.07, 134.00, 129.31, 127.39 (2C), 127.28, 120.34, 119.14, 116.41, 114.52 (2C), 85.13, 71.92, 68.32, 57.08, 55.28, 52.16, 47.66, 46.28, 40.41, 37.12, 31.87, 29.65, 10.91. HPLC-MS (ESI): purity = 98%, *t*_R_ = 2.542 min, *m/z* [M + H]^+^ = 536.2.

##### 5-Chloro-2-((ethyl(2-((1-(4-methoxybenzyl)-2-(tetrahydro-2*H*-pyran-4-yl)-1*H*-imidazo[4,5-*c*]pyridin-6-yl)amino)ethyl)amino)methyl)phenol (**f.3**)

Obtained from intermediate **e.3** (90 mg, 0.22 mmol) and 4-chloro-2-hydroxybenzaldehyde (41 mg, 0.26 mmol) as an orange-colored sticky solid (54%, 66 mg); Rf (DCM: MeOH, 9:1) 0.44; ^1^H-NMR (600 MHz, chloroform-*d*): δ 8.49 (d, *J* = 1.0 Hz, 1H), 6.92 (d, *J* = 8.7 Hz, 2H), 6.84–6.79 (m, 3H), 6.72 (d, *J* = 2.1 Hz, 1H), 6.66 (dd, *J* = 8.0, 2.1 Hz, 1H), 6.05 (d, *J* = 1.0 Hz, 1H), 5.13 (s, 2H), 4.04–3.97 (m, 2H), 3.74 (s, 3H), 3.73 (s, 2H), 3.43–3.38 (m, 4H), 2.93 (tt, *J* = 11.5, 3.7 Hz, 1H), 2.74 (t, *J* = 6.4 Hz, 2H), 2.63 (q, *J* = 7.1 Hz, 2H), 2.09–2.00 (m, 2H), 1.70–1.63 (m, 2H), 1.04 (t, *J* = 7.1 Hz, 3H). ^13^C-NMR (151 MHz, CDCl_3_): δ 159.39, 158.79, 157.69, 154.31, 143.61, 139.03, 133.98, 129.32, 127.38, 127.33 (2C), 125.25, 120.35, 119.12, 116.39, 114.49 (2C), 85.35, 67.48 (2C), 57.04, 55.27, 52.15, 47.64, 46.22, 40.40, 33.80, 31.24 (2C), 10.90. HPLC-MS (ESI): purity = 98%, *t*_R_ = 2.531 min, *m/z* [M + H]^+^ = 550.2.

### General Procedure for the Synthesis of Target Compounds **1**–**19**

The appropriate intermediate **f.1**–**f.19** was stirred in neat TFA (10 mL) at 100 °C for 16 h. Once the reaction was complete, TFA was removed under reduced pressure. The residue was dissolved in DCM/MeOH (9:1) and stirred with Amberlyst A21 for 1 h. The resin was filtered off, and the filtrate was concentrated under reduced pressure. The residue was purified via column chromatography to obtain the final product.

#### 5-Chloro-2-(((2-((2-cyclopentyl-1*H*-imidazo[4,5-*c*]pyridin-6-yl)amino)ethyl)(ethyl)amino)methyl)phenol (**1**)

Obtained from intermediate **f.1** (116 mg, 0.22 mmol) as an off-white sticky solid (68%, 61 mg); Rf (DCM: MeOH, 9:1) 0.38; ^1^H-NMR (600 MHz, DMSO-*d*_6_): δ 8.18 (d, *J* = 1.0 Hz, 1H), 7.25 (d, *J* = 8.1 Hz, 1H), 6.81 (d, *J* = 2.1 Hz, 1H), 6.79 (dd, *J* = 8.1, 2.1 Hz, 1H), 6.44 (d, *J* = 1.0 Hz, 1H), 4.03 (s, 2H), 3.40 (t, *J* = 5.9 Hz, 2H), 3.20–3.13 (m, 1H), 2.98 (t, *J* = 5.8 Hz, 2H), 2.93 (q, *J* = 7.2 Hz, 2H), 2.02–1.95 (m, 2H), 1.84–1.77 (m, 2H), 1.73–1.66 (m, 2H), 1.63–1.56 (m, 2H), 1.13 (t, *J* = 7.2 Hz, 3H). ^13^C-NMR (151 MHz, DMSO): δ 158.76, 154.94, 137.73, 132.39, 130.71, 130.00, 129.65, 128.84, 122.94, 118.83, 115.55, 86.67, 54.92, 52.22, 49.03, 47.10, 39.29, 31.92 (2C), 25.54 (2C), 11.31. HPLC-MS (ESI): purity = 98%, *t*_R_ = 2.203 min, *m/z* [M + H]^+^ = 414.1.

#### 5-Chloro-2-((ethyl(2-((2-(tetrahydrofuran-3-yl)-1*H*-imidazo[4,5-*c*]pyridin-6-yl)amino)ethyl)amino)methyl)phenol (**2**)

Obtained from intermediate **f.2** (113 mg, 0.21 mmol) as an off-white sticky solid (74%, 65 mg); Rf (DCM: MeOH, 9:1) 0.34; ^1^H-NMR (600 MHz, DMSO-*d*_6_): δ 8.21 (d, *J* = 1.0 Hz, 1H), 7.08 (d, *J* = 8.0 Hz, 1H), 6.73 (d, *J* = 2.1 Hz, 1H), 6.71 (dd, *J* = 8.0, 2.1 Hz, 1H), 6.32 (d, *J* = 1.0 Hz, 1H), 4.02–3.99 (m, 1H), 3.83 (t, *J* = 6.7 Hz, 2H), 3.78–3.73 (m, 1H), 3.71 (s, 2H), 3.56–3.49 (m, 1H), 3.35–3.31 (m, 2H), 2.65 (t, *J* = 6.7 Hz, 2H), 2.56 (q, *J* = 7.1 Hz, 2H), 2.28–2.17 (m, 2H), 0.98 (t, *J* = 7.1 Hz, 3H). ^13^C-NMR (151 MHz, DMSO): δ 158.75, 155.54, 155.12, 142.76, 138.05, 134.83, 132.38, 130.70, 130.00, 122.96, 118.83, 115.55, 86.69, 71.63, 67.89, 54.93, 52.19, 49.03, 47.10, 31.48, 11.32. HPLC-MS (ESI): purity = 98%, *t*_R_ = 0.298 min, *m/z* [M + H]^+^ = 416.2.

#### 5-Chloro-2-((ethyl(2-((2-(tetrahydro-2*H*-pyran-4-yl)-1*H*-imidazo[4,5-*c*]pyridin-6-yl)amino)ethyl)amino)methyl)phenol (**3**)

Obtained from intermediate **f.3** (66 mg, 0.12 mmol) as an off-white sticky solid (80%, 41 mg); Rf (DCM: MeOH, 9:1) 0.29; ^1^H-NMR (600 MHz, DMSO-*d*_6_): δ 8.21 (d, *J* = 1.0 Hz, 1H), 7.15 (d, *J* = 7.9 Hz, 1H), 6.76 (d, *J* = 2.1 Hz, 1H), 6.74 (dd, *J* = 7.9, 2.1 Hz, 1H), 6.38 (d, *J* = 1.0 Hz, 1H), 3.91–3.86 (m, 2H), 3.83 (s, 2H), 3.44–3.39 (m, 2H), 3.36 (t, *J* = 6.4 Hz, 2H), 3.03–2.96 (m, 1H), 2.78 (t, *J* = 6.4 Hz, 2H), 2.71 (q, *J* = 7.2 Hz, 2H), 1.90–1.85 (m, 2H), 1.79–1.72 (m, 2H), 1.04 (t, *J* = 7.1 Hz, 3H). ^13^C-NMR (151 MHz, DMSO): δ 158.55, 154.84, 142.80, 137.40, 133.97, 133.06, 131.58, 130.00, 129.68, 128.96, 119.01, 115.57, 66.96 (2C), 54.16, 52.89, 49.03, 47.44, 35.17, 31.12 (2C), 10.84. HPLC-MS (ESI): purity = 97%, *t*_R_ = 0.430 min, *m/z* [M + H]^+^ = 430.2.

#### 5-Chloro-2-((ethyl(2-((2-methyl-1*H*-imidazo[4,5-*c*]pyridin-6-yl)amino)ethyl)amino)methyl)phenol (**4**)

Obtained from intermediate **f.4** (66 mg, 0.14 mmol) as an off-white sticky solid (71%, 36 mg); Rf (DCM: MeOH, 9:1) 0.26; ^1^H-NMR (600 MHz, DMSO-*d*_6_): δ 8.15 (d, *J* = 1.0 Hz, 1H), 7.08 (d, *J* = 8.0 Hz, 1H), 6.73 (d, *J* = 2.1 Hz, 1H), 6.71 (dd, *J* = 8.0, 2.1 Hz, 1H), 6.29 (d, *J* = 1.0 Hz, 1H), 3.70 (s, 2H), 3.32 (t, *J* = 6.4 Hz, 2H), 2.64 (t, *J* = 6.4 Hz, 2H), 2.55 (q, *J* = 7.2 Hz, 2H), 2.35 (s, 3H), 0.98 (t, *J* = 7.1 Hz, 3H). ^13^C-NMR (151 MHz, DMSO): δ 158.75, 154.96, 151.25, 137.45, 132.35, 130.69, 130.01, 123.02, 118.83, 115.55, 86.62, 63.28, 54.92, 52.20, 49.04, 47.10, 15.01, 11.35. HPLC-MS (ESI): purity = 98%, *t*_R_ = 0.219 min, *m/z* [M + H]^+^ = 360.1.

#### 5-Chloro-2-(((2-((2-cyclopropyl-1*H*-imidazo[4,5-*c*]pyridin-6-yl)amino)ethyl)(ethyl)amino)methyl)phenol (**5**)

Obtained from intermediate **f.5** (351 mg, 0.69 mmol) as an off-white sticky solid (68%, 181 mg); Rf (DCM: MeOH, 9:1) 0.30; ^1^H-NMR (600 MHz, DMSO-*d*_6_): δ 8.11 (d, *J* = 1.0 Hz, 1H), 7.10 (d, *J* = 8.0 Hz, 1H), 6.74 (d, *J* = 2.2 Hz, 1H), 6.72 (dd, *J* = 8.0, 2.1 Hz, 1H), 6.31 (d, *J* = 1.0 Hz, 1H), 3.74 (s, 2H), 3.32 (t, *J* = 6.7 Hz, 2H), 2.68 (t, *J* = 6.7 Hz, 2H), 2.59 (q, *J* = 7.2 Hz, 2H), 2.01–1.96 (m, 1H), 0.99 (t, *J* = 7.1 Hz, 3H), 0.95–0.93 (m, 4H). ^13^C-NMR (151 MHz, DMSO): δ 158.69, 157.15, 154.78, 142.64, 137.07, 132.58, 130.97, 130.00, 122.52, 118.88, 115.56, 86.76, 54.69, 52.44, 49.03, 47.20, 11.18, 9.83 (2C), 8.93. HPLC-MS (ESI): purity = 98%, *t*_R_ = 2.211 min, *m/z* [M + H]^+^ = 386.1.

#### 5-Chloro-2-((ethyl(2-((2-(1-methylcyclopropyl)-1*H*-imidazo[4,5-*c*]pyridin-6-yl)amino)ethyl)amino)methyl)phenol (**6**)

Obtained from intermediate **f.6** (381 mg, 0.73 mmol) as an off-white sticky solid (86%, 250 mg); Rf (DCM: MeOH, 9:1) 0.29; ^1^H-NMR (400 MHz, DMSO-*d*_6_): δ 8.37 (d, *J* = 1.0 Hz, 1H), 7.40 (d, *J* = 8.2 Hz, 1H), 6.92 (d, *J* = 2.1 Hz, 1H), 6.87 (dd, *J* = 8.2, 2.1 Hz, 1H), 6.73 (d, *J* = 1.0 Hz, 1H), 4.30 (s, 2H), 3.66 (t, *J* = 6.1 Hz, 2H), 3.28 (t, *J* = 6.0 Hz, 2H), 3.22 (q, *J* = 7.2 Hz, 1H), 1.53 (s, 3H), 1.31–1.26 (m, 5H), 1.04–1.01 (m, 2H). ^13^C-NMR (101 MHz, DMSO): δ 158.06, 152.70, 149.17, 143.15, 140.46, 135.35, 134.45, 131.71, 127.66, 116.35, 115.81, 108.67, 90.14, 51.45, 48.64, 38.07, 20.88, 17.89, 15.68 (2C), 9.19. HPLC-MS (ESI): purity = 98%, *t*_R_ = 0.755 min, *m/z* [M + H]^+^ = 400.2.

#### 5-Chloro-2-((ethyl(2-((2-(1-(trifluoromethyl)cyclopropyl)-1*H*-imidazo[4,5-*c*]pyridin-6-yl)amino)ethyl)amino)methyl)phenol (**7**)

Obtained from intermediate **f.7** (59 mg, 0.10 mmol) as an off-white sticky solid (77%, 35 mg); Rf (DCM: MeOH, 9:1) 0.30; ^1^H-NMR (400 MHz, DMSO-*d*_6_): δ 8.50 (d, *J* = 1.0 Hz, 1H), 7.41 (d, *J* = 8.2 Hz, 1H), 6.93 (d, *J* = 2.1 Hz, 1H), 6.88 (dd, *J* = 8.1, 2.1 Hz, 1H), 6.74 (d, *J* = 1.0 Hz, 1H), 4.32 (s, 2H), 3.65 (t, *J* = 5.9 Hz, 2H), 3.30 (t, *J* = 5.9 Hz, 2H), 3.24 (q, *J* = 7.1 Hz, 2H), 1.60–1.55 (m, 4H), 1.29 (t, *J* = 7.2 Hz, 3H). ^13^C-NMR (101 MHz, DMSO): δ 158.05, 152.50, 149.26, 146.22, 141.57, 135.40, 134.48, 129.93, 124.43, 119.59, 116.31, 115.81, 111.78, 51.57, 48.70, 38.22, 31.43, 23.37, 12.03 (2C), 9.18. HPLC-MS (ESI): purity = 98%, *t*_R_ = 2.194 min, *m/z* [M + H]^+^ = 454.1.

#### 5-Chloro-2-(((2-((2-(cyclopropylmethyl)-1*H*-imidazo[4,5-*c*]pyridin-6-yl)amino)ethyl)(ethyl)amino)methyl)phenol (**8**)

Obtained from intermediate **f.8** (239 mg, 0.46 mmol) as an off-white sticky solid (88%, 162 mg); Rf (DCM: MeOH, 9:1) 0.35; ^1^H-NMR (400 MHz, DMSO-*d*_6_): δ 8.46 (d, *J* = 1.0 Hz, 1H), 7.41 (d, *J* = 8.2 Hz, 1H), 6.94 (d, *J* = 2.1 Hz, 1H), 6.87 (dd, *J* = 8.1, 2.1 Hz, 1H), 6.79 (d, *J* = 1.0 Hz, 1H), 4.31 (s, 2H), 3.68 (t, *J* = 6.0 Hz, 2H), 3.30 (t, *J* = 6.0 Hz, 2H), 3.24 (q, *J* = 7.2 Hz, 2H), 2.80 (d, *J* = 7.0 Hz, 2H), 1.30 (t, *J* = 7.2 Hz, 3H), 1.20–1.15 (m, 1H), 0.57–0.52 (m, 2H), 0.34–0.29 (m, 2H). ^13^C-NMR (101 MHz, DMSO): δ 159.52, 158.11, 152.35, 146.06, 135.35, 134.43, 131.03, 125.99, 119.54, 116.36, 115.84, 89.98, 56.56, 52.04, 51.47, 48.64, 38.03, 33.06, 9.19, 4.94 (2C). HPLC-MS (ESI): purity = 98%, *t*_R_ = 0.588 min, *m/z* [M + H]^+^ = 400.2.

#### 5-Chloro-2-(((2-((2-cyclobutyl-1*H*-imidazo[4,5-*c*]pyridin-6-yl)amino)ethyl)(ethyl)amino)methyl)phenol (**9**)

Obtained from intermediate **f.9** (311 mg, 0.60 mmol) as an off-white sticky solid (79%, 191 mg); Rf (DCM: MeOH, 9:1) 0.31; ^1^H-NMR (400 MHz, DMSO-*d*_6_): δ 8.45 (d, *J* = 1.0 Hz, 1H), 7.41 (d, *J* = 8.2 Hz, 1H), 6.94 (d, *J* = 2.1 Hz, 1H), 6.85 (dd, *J* = 8.2, 2.1 Hz, 1H), 6.78 (d, *J* = 1.0 Hz, 1H), 4.31 (s, 2H), 3.83–3.72 (m, 1H), 3.69 (t, *J* = 6.1 Hz, 2H), 3.29 (t, *J* = 6.0 Hz, 2H), 3.23 (q, *J* = 7.2 Hz, 2H), 2.46–2.34 (m, 4H), 2.15–2.02 (m, 1H), 2.00–1.89 (m, 1H), 1.30 (t, *J* = 7.1 Hz, 3H). ^13^C-NMR (101 MHz, DMSO) δ 163.14, 159.51, 158.11, 151.94, 135.38, 134.37, 119.50, 116.27, 115.86, 89.93, 79.09, 51.89, 51.45, 48.65, 40.46, 38.02, 33.40, 27.37 (2C), 18.60, 9.19. HPLC-MS (ESI): purity = 98%, *t*_R_ = 0.921 min, *m/z* [M + H]^+^ = 400.2.

#### 5-Chloro-2-(((2-((2-(3,3-difluorocyclobutyl)-1*H*-imidazo[4,5-*c*]pyridin-6-yl)amino)ethyl)(ethyl)amino)methyl)phenol (**10**)

Obtained from intermediate **f.10** (274 mg, 0.49 mmol) as an off-white sticky solid (84%, 179 mg); Rf (DCM: MeOH, 9:1) 0.29; ^1^H-NMR (600 MHz, DMSO-*d*_6_): δ 8.51 (d, *J* = 1.0 Hz, 1H), 7.37 (d, *J* = 8.2 Hz, 1H), 6.88 (d, *J* = 2.1 Hz, 1H), 6.84 (dd, *J* = 8.2, 2.1 Hz, 1H), 6.80 (d, *J* = 1.0 Hz, 1H), 4.28 (s, 2H), 3.66 (t, *J* = 6.3 Hz, 2H), 3.64–3.60 (m, 1H), 3.27 (t, *J* = 6.2 Hz, 2H), 3.20 (q, *J* = 7.2 Hz, 2H), 3.10–2.94 (m, 4H), 1.26 (t, *J* = 7.2 Hz, 3H). ^13^C-NMR (151 MHz, DMSO): δ 159.36, 158.02, 150.81, 147.81, 135.40, 134.50, 133.25, 122.12, 120.25, 119.58, 118.45, 116.14, 115.75, 89.93, 63.24, 51.32, 48.61, 37.86, 21.96 (2C), 9.11. HPLC-MS (ESI): purity = 99%, *t*_R_ = 1.808 min, *m/z* [M + H]^+^ = 436.1.

#### 5-Chloro-2-(((2-((2-cyclohexyl-1*H*-imidazo[4,5-*c*]pyridin-6-yl)amino)ethyl)(ethyl)amino)methyl)phenol (**11**)

Obtained from intermediate **f.11** (274 mg, 0.50 mmol) as an off-white sticky solid (71%, 152 mg); Rf (DCM: MeOH, 9:1) 0.32; ^1^H-NMR (600 MHz, DMSO-*d*_6_): δ 8.44 (d, *J* = 1.0 Hz, 1H), 7.36 (d, *J* = 8.2 Hz, 1H), 6.88 (d, *J* = 2.1 Hz, 1H), 6.82 (dd, *J* = 8.2, 2.1 Hz, 1H), 6.76 (d, *J* = 1.0 Hz, 1H), 4.26 (s, 2H), 3.65 (t, *J* = 6.2 Hz, 2H), 3.25 (t, *J* = 6.2 Hz, 2H), 3.19 (q, *J* = 7.2 Hz, 2H), 2.89 (tt, *J* = 11.7, 3.7 Hz, 1H), 2.01–1.96 (m, 2H), 1.77–1.73 (m, 2H), 1.67–1.63 (m, 1H), 1.59–1.52 (m, 2H), 1.39–1.30 (m, 2H), 1.25 (t, *J* = 7.2 Hz, 3H), 1.23–1.17 (m, 1H). ^13^C-NMR (151 MHz, DMSO): δ 164.77, 159.42, 158.04, 151.60, 146.44, 135.37, 134.44, 130.11, 126.08, 119.51, 116.14, 115.75, 110.92, 89.86, 51.33, 48.63, 37.63, 30.78 (2C), 25.69, 25.57 (2C), 9.09. HPLC-MS (ESI): purity = 97%, *t*_R_ = 2.238 min, *m/z* [M + H]^+^ = 428.2.

#### 5-Chloro-2-(((2-((2-(4,4-difluorocyclohexyl)-1*H*-imidazo[4,5-*c*]pyridin-6-yl)amino)ethyl)(ethyl)amino)methyl)phenol (**12**)

Obtained from intermediate **f.12** (381 mg, 0.65 mmol) as an off-white sticky solid (83%, 250 mg); Rf (DCM: MeOH, 9:1) 0.37; ^1^H-NMR (600 MHz, DMSO-*d*_6_): δ 8.46 (d, *J* = 1.0 Hz, 1H), 7.36 (d, *J* = 8.2 Hz, 1H), 6.89 (d, *J* = 2.1 Hz, 1H), 6.81 (dd, *J* = 8.2, 2.1 Hz, 1H), 6.80 (d, *J* = 1.0 Hz, 1H), 4.27 (s, 2H), 3.76–3.70 (m, 1H), 3.67 (t, *J* = 6.3 Hz, 2H), 3.27 (t, *J* = 6.2 Hz, 2H), 3.19 (q, *J* = 7.1 Hz, 2H), 3.12–3.06 (m, 1H), 2.11–2.07 (m, 4H), 2.01–1.89 (m, 1H), 1.88–1.80 (m, 2H), 1.25 (t, *J* = 7.2 Hz, 3H). ^13^C-NMR (151 MHz, DMSO): δ 158.09, 150.96, 135.35, 134.42, 129.70, 125.56, 123.97, 122.38, 119.46, 116.12, 115.78, 89.88, 62.47, 51.26, 48.62, 37.80, 35.06, 32.56, 27.12 (2C), 25.84 (2C), 9.08. HPLC-MS (ESI): purity = 98%, *t*_R_ = 2.150 min, *m/z* [M + H]^+^ = 464.2.

#### 5-Chloro-2-((ethyl(2-((2-(1-methylpyrrolidin-2-yl)-1*H*-imidazo[4,5-*c*]pyridin-6-yl)amino)ethyl)amino)methyl)phenol (**13**)

Obtained from intermediate **f.13** (51 mg, 0.10 mmol) as a yellow sticky solid (71%, 31 mg); Rf (DCM: MeOH, 9:1) 0.20; ^1^H-NMR (600 MHz, DMSO-*d*_6_): δ 8.44 (d, *J* = 1.0 Hz, 1H), 7.38 (d, *J* = 8.2 Hz, 1H), 6.92 (d, *J* = 2.1 Hz, 1H), 6.83 (dd, *J* = 8.2, 2.1 Hz, 1H), 6.66 (d, *J* = 1.0 Hz, 1H), 4.77 (dd, *J* = 8.1, 4.3 Hz, 1H), 4.27 (s, 2H), 3.58 (t, *J* = 5.9 Hz, 2H), 3.24 (t, *J* = 5.9 Hz, 2H), 3.20 (q, *J* = 7.2 Hz, 2H), 2.94–2.86 (m, 2H), 2.60–2.53 (m, 2H), 2.27–1.98 (m, 2H), 1.72 (s, 3H), 1.24 (t, *J* = 7.2 Hz, 3H). ^13^C-NMR (151 MHz, DMSO): δ 172.03, 159.23, 158.81, 158.11, 153.85, 135.31, 134.45, 120.46, 119.47, 118.48, 116.50, 116.33, 115.80, 114.52, 52.89, 51.50, 48.58, 38.13, 29.75, 22.88, 22.29, 9.14. HPLC-MS (ESI): purity = 97%, *t*_R_ = 0.141 min, *m/z* [M + H]^+^ = 429.2.

#### 5-Chloro-2-((ethyl(2-((2-(3-(trifluoromethyl)phenyl)-1*H*-imidazo[4,5-*c*]pyridin-6-yl)amino)ethyl)amino)methyl)phenol (**14**)

Obtained from intermediate **f.14** (231 mg, 0.38 mmol) as an off-white sticky solid (81%, 151 mg); Rf (DCM: MeOH, 9:1) 0.30; ^1^H-NMR (600 MHz, DMSO-*d*_6_): δ 8.53 (d, *J* = 1.0 Hz, 1H), 8.47 (dd, *J* = 2.1, 1.5 Hz, 1H), 8.44 (ddd, *J* = 7.8, 1.6, 1.5 Hz, 1H), 7.88 (ddd, *J* = 7.8, 2.1, 1.6 Hz, 1H), 7.79 (t, *J* = 7.8 Hz, 1H), 7.41 (d, *J* = 8.2 Hz, 1H), 6.90 (d, *J* = 2.1 Hz, 1H), 6.85 (dd, *J* = 8.2, 2.1 Hz, 1H), 6.75 (d, *J* = 1.0 Hz, 1H), 4.28 (s, 2H), 3.64 (t, *J* = 6.0 Hz, 2H), 3.26 (t, *J* = 5.9 Hz, 2H), 3.21 (q, *J* = 7.2 Hz, 2H), 1.27 (t, *J* = 7.2 Hz, 3H). ^13^C-NMR (151 MHz, DMSO): δ 158.96, 158.75, 158.54, 158.32, 158.04, 135.32, 134.55, 131.14, 130.85, 130.47, 127.62, 125.27, 123.74, 120.50, 120.36, 119.56, 118.39, 116.28, 115.75, 51.39, 48.58, 38.16, 22.91, 9.16. HPLC-MS (ESI): purity = 98%, *t*_R_ = 2.422 min, *m/z* [M + H]^+^ = 490.1.

#### 5-Chloro-2-(((2-((2-cycloheptyl-1*H*-imidazo[4,5-*c*]pyridin-6-yl)amino)ethyl)(ethyl)amino)methyl)phenol (**15**)

Obtained from intermediate **f.15** (76 mg, 0.14 mmol) as an off-white sticky solid (92%, 57 mg); Rf (DCM: MeOH, 9:1) 0.38; ^1^H-NMR (600 MHz, DMSO-*d*_6_): δ 8.41 (d, *J* = 1.0 Hz, 1H), 7.37 (d, *J* = 8.2 Hz, 1H), 6.89 (d, *J* = 2.2 Hz, 1H), 6.82 (dd, *J* = 8.2, 2.1 Hz, 1H), 6.74 (d, *J* = 1.0 Hz, 1H), 4.26 (s, 2H), 3.64 (t, *J* = 6.2 Hz, 2H), 3.25 (t, *J* = 6.2 Hz, 2H), 3.19 (q, *J* = 7.2 Hz, 2H), 3.07 (tt, *J* = 9.4, 4.4 Hz, 1H), 2.03–1.96 (m, 2H), 1.84–1.76 (m, 2H), 1.62–1.56 (m, 4H), 1.55–1.48 (m, 4H), 1.25 (t, *J* = 7.1 Hz, 3H). ^13^C-NMR (151 MHz, DMSO): δ 172.01, 159.28, 159.06, 158.85, 158.64, 158.06, 135.31, 134.44, 119.49, 118.40, 116.22, 115.76, 89.77, 51.31, 48.60, 37.84, 32.67 (2C), 28.14 (2C), 26.17 (2C), 22.88, 9.12. HPLC-MS (ESI): purity = 99%, *t*_R_ = 2.413 min, *m/z* [M + H]^+^ = 442.2.

#### 5-Chloro-2-((ethyl(2-((2-((1r,4r)-4-(trifluoromethyl)cyclohexyl)-1*H*-imidazo[4,5-*c*]pyridin-6-yl)amino)ethyl)amino)methyl)phenol (**16**)

Obtained from intermediate **f.16** (81 mg, 0.13 mmol) as an off-white sticky solid (88%, 57 mg); Rf (DCM: MeOH, 9:1) 0.38; ^1^H-NMR (600 MHz, DMSO-*d*_6_): δ 8.42 (d, *J* = 1.0 Hz, 1H), 7.37 (d, *J* = 8.2 Hz, 1H), 6.89 (d, *J* = 2.1 Hz, 1H), 6.82 (dd, *J* = 8.2, 2.1 Hz, 1H), 6.76 (d, *J* = 1.0 Hz, 1H), 4.26 (s, 2H), 3.64 (t, *J* = 6.2 Hz, 2H), 3.25 (t, *J* = 6.2 Hz, 2H), 3.18 (q, *J* = 7.2 Hz, 2H), 2.89 (tt, *J* = 12.1, 3.6 Hz, 1H), 2.36–2.27 (m, 1H), 2.16–2.12 (m, 2H), 1.98–1.94 (m, 2H), 1.66–1.58 (m, 2H), 1.45–1.35 (m, 2H), 1.25 (t, *J* = 7.2 Hz, 3H). ^13^C-NMR (151 MHz, CDCl_3_): δ 159.44, 158.78, 154.20, 143.67, 138.51, 134.04, 134.02, 129.32, 127.19, 120.34, 119.14, 116.42, 85.28, 57.08, 55.29, 52.10, 47.69, 46.36, 46.24, 41.06, 35.44, 29.99 (2C), 10.89. HPLC-MS (ESI): purity = 98%, *t*_R_ = 2.413 min, *m/z* [M + H]^+^ = 496.2.

#### 5-Chloro-2-((ethyl(2-((2-((1r,4r)-4-(trifluoromethyl)cyclohexyl)-1*H*-imidazo[4,5-*c*]pyridin-6-yl)Amino)ethyl)amino)methyl)phenol (**17**)

Obtained from intermediate **f.17** (77 mg, 0.12 mmol) as an off-white sticky solid (78%, 47 mg); Rf (DCM: MeOH, 9:1) 0.36; ^1^H-NMR (600 MHz, DMSO): δ 8.40 (d, *J* = 1.0 Hz, 1H), 7.36 (d, *J* = 8.2 Hz, 1H), 6.87 (d, *J* = 2.2 Hz, 1H), 6.83 (dd, *J* = 8.2, 2.1 Hz, 1H), 6.71 (d, *J* = 1.0 Hz, 1H), 4.26 (s, 2H), 3.25–3.22 (m, 4H), 3.18 (q, *J* = 7.2 Hz, 2H), 2.88 (tt, *J* = 12.3, 3.7 Hz, 1H), 2.37–2.28 (m, 1H), 2.17–2.12 (m, 2H), 1.99–1.94 (m, 2H), 1.66–1.58 (m, 2H), 1.45–1.36 (m, 2H), 1.24 (t, *J* = 7.2 Hz, 3H). ^13^C-NMR (151 MHz, CDCl_3_): δ 159.44, 158.78, 154.20, 143.67, 138.51, 134.04, 134.02, 129.32, 127.19, 120.34, 119.14, 116.42, 85.28, 57.08, 55.29, 52.10, 47.69, 46.36, 46.24, 41.06, 35.44, 29.99 (2C), 10.89. HPLC-MS (ESI): purity = 97%, *t*_R_ = 2.343 min, *m/z* [M + H]^+^ = 496.2.

#### 5-Chloro-2-((ethyl(2-((2-(4-methoxycyclohexyl)-1*H*-imidazo[4,5-*c*]pyridin-6-yl)amino)ethyl)amino)methyl)phenol (**18**)

Obtained from intermediate **f.18** (73 mg, 0.13 mmol) as an off-white sticky solid (77%, 46 mg); Rf (DCM: MeOH, 9:1) 0.50; ^1^H-NMR (600 MHz, DMSO): δ 8.37 (d, *J* = 1.0 Hz, 1H), 8.13 (d, *J* = 8.1 Hz, 1H), 6.89 (d, *J* = 2.1 Hz, 1H), 6.83 (dd, *J* = 8.2, 2.1 Hz, 1H), 6.62 (d, *J* = 1.0 Hz, 1H), 4.25 (s, 2H), 3.64–3.53 (m, 1H), 3.22 (s, 3H), 3.12 (tt, *J* = 10.88, 4.11 Hz, 1H), 2.92 (t, *J* = 6.2 Hz, 2H), 2.81 (t, *J* = 6.2 Hz, 2H), 2.58 (q, *J* = 7.2 Hz, 2H), 2.08–2.02 (m, 2H), 1.88–1.77 (m, 2H), 1.75–1.71 (m, 2H), 1.63–1.47 (m, 2H), 1.23 (t, *J* = 7.2 Hz, 3H). ^13^C-NMR (151 MHz, CDCl_3_): δ 159.47, 153.50, 144.28, 136.87, 134.14, 129.50, 127.40, 119.22, 116.44, 85.54, 78.32, 73.57, 56.94, 55.77, 55.30, 52.01, 47.89, 40.43, 35.67, 31.57 (2C), 29.79 (2C), 10.87. HPLC-MS (ESI): purity = 97%, *t*_R_ = 2.124 min, *m/z* [M + H]^+^ = 458.2.

#### 5-Chloro-2-((ethyl(2-((2-(6-(trifluoromethyl)pyridin-3-yl)-1*H*-imidazo[4,5-*c*]pyridin-6-yl)amino)ethyl)amino)methyl)phenol (**19**)

Obtained from intermediate **f.19** (55 mg, 0.09 mmol) as an off-white solid (81%, 36 mg); Rf (DCM: MeOH, 9:1) 0.38; ^1^H-NMR (600 MHz, DMSO-*d*_6_): δ 9.44 (d, *J* = 2.1 Hz, 1H), 8.70 (dd, *J* = 8.2, 2.1 Hz, 1H), 8.52 (d, *J* = 1.0 Hz, 1H, ), 8.09 (d, *J* = 8.2 Hz, 1H), 7.38 (d, *J* = 8.2 Hz, 1H), 6.89 (d, *J* = 2.1 Hz, 1H), 6.85 (dd, *J* = 8.2, 2.1 Hz, 1H), 6.67 (d, *J* = 1.0 Hz, 1H), 4.28 (s, 2H), 3.59 (t, *J* = 5.8 Hz, 2H), 3.25 (t, *J* = 5.9 Hz, 2H), 3.21 (q, *J* = 7.2 Hz, 2H), 1.27 (t, *J* = 7.2 Hz, 3H).C-NMR (151 MHz, CDCl_3_): δ 159.54, 158.60, 155.00, 149.46, 144.49, 140.74, 137.88, 134.69, 134.25, 129.57, 128.82, 126.84, 120.39, 119.88, 119.26, 114.79, 85.39, 56.84, 55.31, 52.20, 47.84, 41.84, 10.77. HPLC-MS (ESI): purity = 98%, *t*_R_ = 2.352 min, *m/z* [M + H]^+^ = 491.1.

#### 5-Chloro-2-(((2-((7-nitrobenzo[c][1,2,5]oxadiazol-4-yl)amino)ethyl)(2-((2-(3-(trifluoromethyl)phenyl)-1*H*-imidazo[4,5-*c*]pyridin-6-yl)amino)ethyl)amino)methyl)phenol (**14-NBD**)

Obtained from intermediate **m** (339 mg, 0.56 mmols) and NBD-chloride (254 mg, 1.27 mmols) as a brick-red solid (50%, 187 mg); R_f_ (DCM: MeOH, 9:1) 0.56; ^1^H NMR (600 MHz, DMSO-*d*_6_): δ 8.39 (s, 1H), 8.37 (d, *J* = 8.8 Hz, 1H), 8.35 (dd, *J* = 2.8, 2.2 Hz, 1H), 8.31 (d, *J* = 8.8 Hz, 1H), 7.82 (dd, *J* = 7.8, 2.2 Hz, 1H), 7.74 (ddd, *J* = 7.8, 2.8, 2.3 Hz, 1H), 7.13 (dd, *J* = 8.4, 7.8 Hz, 1H), 6.85 (d, *J* = 7.8 Hz, 1H), 6.59 (ddd, *J* = 8.4, 2.3 Hz, 1H), 6.19 (d, *J* = 2.2 Hz, 1H), 6.04 (s, 1H), 3.70 (s, 2H), 3.53 (t, *J* = 6.3 Hz, 2H), 3.39 (t, *J* = 6.0 Hz, 2H), 2.82–2.76 (m, 4H). ^13^C NMR (151 MHz, DMSO): δ 159.13, 158.92, 157.57, 155.85, 154.95, 149.82, 147.41, 143.12, 139.53, 138.17, 137.90, 135.58, 132.31, 131.80, 131.35, 129.01, 128.78, 126.30, 123.09, 118.92, 115.35, 99.50, 92.26, 86.51, 55.54, 53.70, 51.38, 44.39, 41.56, 36.89. HPLC-MS (ESI): purity = 98%, *t*_R_ = 0.905 min, m/z [M + H]^+^ = 668.2.

### In Vitro *P. falciparum* Assay

Compounds were screened against multi-drug-resistant (K1) and sensitive (NF54) strains of *P. falciparum* in vitro using a parasite lactate dehydrogenase assay (pLDH) (the method is described fully in the Supporting Information).

### In Vitro Cytotoxicity Assay

In vitro cytotoxicity was performed on the Chinese Hamster Ovarian cell line by measuring cellular growth and survival calorimetrically through the MTT assay.^29,30^ The formation of tetrazolium salt was used as a measure of chemosensitivity and growth. (Details of this assay is described in the Supporting Information.)

### In Vitro Microsomal Stability Assay

The in vitro microsomal stability assay was performed in duplicate in a 96-well microtiter plate using a single-point experiment design.^31^ The test compounds (1 μM) were incubated individually in human (pool of 50, mixed-gender), rat (pool of 711, male Sprague Dawley) and mouse (pool of 1634, male CD1) liver microsomes (final protein concentration of 0.4 mg/mL; Xenotech, Kansas, USA), suspended in 0.1 M phosphate buffer (pH 7.4). (Details of this assay are described in the Supporting Information.)

## Supporting Information Available

Additional details on the characterization of all intermediates and target compounds as well as methods used for the in vitro antimalarial, ADME, and mode of action studies (PDF)

## Abbreviations Used

ADME: absorption, distribution, metabolism, and excretion
EDCI: 1-ethyl-3-(3-dimethylaminopropyl)carbodiimide
DMAP: 4-dimethylaminopyridine
DMF: *N*,*N*-dimethylformamide
DMAP: 4-dimethylaminopyridine
DCM: dichloromethane
DV: digestive vacuole
PMB: *para*-methoxybenzyl
TFA: trifluoroacetic acid anhydride
*P. falciparum*: *Plasmodium falciparum*
*P. berghei*: *Plasmodium berghei*
SI: selectivity index
CQ: chloroquine
AQ: amodiaquine
CHO: Chinese hamster ovarian
m.p.: melting point
*E*_H_: extraction ratio
IC_50_: concentration of a drug that is required for 50% inhibition in vitro
MTT: 3-(4,5-dimethylthiazol-2-yl)-2,5-diphenyltetrazolium bromide
