# Supplementary material for: Antimalarial Imidazopyridines Incorporating an Intramolecular Hydrogen Bonding Motif: Medicinal Chemistry and Mechanistic Studies
Source: ACS Infect Dis. 2023 Mar 22;9(4):928–42. doi: 10.1021/acsinfecdis.2c00584 (PMC10111423; doi:10.1021/acsinfecdis.2c00584)
Supplement: Supplementary file 1 — id2c00584_si_001.pdf [file id2c00584_si_001.pdf]

Supporting Information

Antimalarial Imidazopyridines incorporating an Intramolecular Hydrogen Bonding Motif: Medicinal Chemistry and Mechanistic Studies

Henrietta D. Attram,[†] Constance M. Korkor,[†] Dale Taylor,[†] Mathew Njoroge,[†] and Kelly Chibale,[†, §, Π, Ω,*]

[†] Department of Chemistry, University of Cape Town, Rondebosch 7701, South Africa

[§]*Drug Discovery and Development Centre (H3D), DMPK & Pharmacology, University of Cape Town, Observatory 7925, South Africa.*

[Π]*South African Medical Research Council Drug Discovery and Development Research Unit, University of Cape Town, Rondebosch 7701, South Africa.*

[Ω]*Institute of Infectious Disease and Molecular Medicine, University of Cape Town, Rondebosch 7701, South Africa*

Corresponding author

*(K.C.) E-mail: kelly.chibale@uct.ac.za. Phone: +27-21-6502553. Fax: +27-21-6505195.

Characterization data for compounds not shown in the main manuscript

***tert*-Butylethyl(2-((1-(4-methoxybenzyl)-2-methyl-1*H*-imidazo[4,5-c]pyridin-6-yl)amino)ethyl)carbamate (e.4).** Obtained from intermediat**e c** (500 mg, 1.20 mmol) and acetic acid (0.02 mg, 1.56 mmol) as a wine-colored sticky solid (43%, 228 mg); Rf (DCM:MeOH, 9:1) 0.53; ^{1}H-NMR (600 MHz, Chloroform-*d*) δ 8.37 (s, 1H), 6.97 (d, *J* = 8.7 Hz, 2H), 6.78 (d, *J* = 8.7 Hz, 2H), 6.36 (s, 1H), 5.07 (s, 2H), 3.71 (s, 3H), 3.37–3.32 (m, 4H), 3.18 (q, *J* = 7.3 Hz, 2H), 2.43 (s, 3H), 1.38 (s, 9H), 1.02 (t, *J* = 7.1 Hz, 3H). ^{13}C-NMR (151 MHz, $CDCl_3$) δ 159.33, 156.09, 154.66, 152.26, 144.07, 137.58, 133.73, 127.77, 127.29 (2C), 114.38 (2C), 84.77, 79.47, 55.23, 46.55, 46.00, 42.98, 41.98, 29.62, 28.39 (3C), 13.93. HPLC-MS (ESI): Purity = 97%, t_R = 2.422 min, *m/z* $[M+H]^+$ = 440.2.

***tert*-Butyl(2-((cyclopropyl-1-(4-methoxybenzyl)-1*H*-imidazo[4,5-c]pyridin-6-yl)amino)ethyl)(ethyl)carbamate (e.5).** Obtained from intermediat**e c** (500 mg, 1.20 mmol) and cyclopropane carboxylic acid (286 mg, 1.56 mmol) as a wine-colored sticky solid (51%, 281 mg); Rf (DCM:MeOH, 9:1) 0.58; ^{1}H-NMR (600 MHz, Chloroform-*d*) δ 8.14 (s, 1H), 7.13 (d, *J* = 8.7 Hz, 2H), 6.96 (s, 1H), 6.77 (d, *J* = 8.7 Hz, 2H), 5.25 (s, 2H), 3.69 (s, 3H), 3.30–3.26 (m, 4H), 3.17 (q, *J* = 7.1 Hz, 2H), 1.51 (tt, *J* = 8.0, 4.6 Hz, 1H), 1.38 (s, 9H), 1.01 (t, *J* = 7.0 Hz, 3H), 0.92–0.89 (m, 2H), 0.73–0.70 (m, 2H). ^{13}C-NMR (151 MHz, $CDCl_3$) δ 179.93, 177.01, 159.37, 155.81, 153.27, 146.39, 132.22, 128.26 (2C), 127.16, 114.33 (2C), 85.19, 79.42, 55.20, 46.48, 45.58, 43.33, 41.26, 28.36 (3C), 22.23, 13.53, 8.83, 7.94. HPLC-MS (ESI): Purity = 98%, t_R = 2.483 min, *m/z* $[M+H]^+$ = 466.2.

***tert*-Butylethyl(2-((1-(4-methoxybenzyl)-2-(1-methylcyclopropyl)-1*H*-imidazo[4,5-c]pyridin-6-yl)amino)ethyl)carbamate (e.6).** Obtained from intermediat**e c** (500 mg, 1.20 mmol) and 1-methylcyclopropane-1-carboxylic acid (0.13 mL, 1.56 mmol) as a wine-colored sticky solid (59%, 339 mg); Rf (DCM:MeOH, 9:1) 0.60; ^{1}H-NMR (400 MHz, Chloroform-*d*) δ 8.19 (s, 1H), 7.02 (d, *J* = 8.2 Hz, 2H), 6.87–6.83 (m, 3H), 5.45 (s, 2H), 3.77 (s, 3H), 3.46–3.17 (m, 6H), 1.42 (s, 9H), 1.24 (t, *J* = 7.1 Hz, 3H), 1.14 (s, 3H), 1.11–1.03 (m, 2H), 0.90–0.86 (m, 2H). ^{13}C-NMR (101 MHz, CDCl3) δ 159.55, 151.30, 132.47, 128.34, 127.62 (2C), 126.01, 114.55 (2C), 87.14, 79.77, 60.31, 55.27, 53.38, 47.41, 47.25, 45.41, 43.64, 41.26, 29.63, 28.36 (3C), 23.18, 14.38, 14.15, 13.63. HPLC-MS (ESI): Purity = 97%, t_R = 2.554 min, *m/z* $[M+H]^+$ = 480.3.

***tert*-Butylethyl(2-((1-(4-methoxybenzyl)-2-(1-(trifluoromethyl)cyclopropyl)-1*H*-imidazo[4,5-c]pyridin-6-yl)amino)ethyl)carbamate (e.7).** Obtained from intermediat**e c** (500 mg, 1.20 mmol) and 1-(trifluoromethyl)cyclopropane-1-carboxylic acid (240 mg, 1.56 mmol) as a wine-colored sticky solid (22%, 140 mg); Rf (DCM:MeOH, 9:1) 0.59; ^{1}H-NMR (400 MHz, Chloroform-*d*) δ 8.52 (s, 1H), 6.95 (d, *J* = 8.7 Hz, 2H), 6.90 (s, 1H), 6.86 (d, *J* = 8.7 Hz, 2H), 5.42 (s, 2H), 3.80 (s, 3H), 3.36–3.18 (m, 6H), 1.44 (s, 9H), 1.25–1.04 (m, 7H). ^{13}C-NMR (101 MHz, CDCl3) δ 159.38, 154.75, 149.39, 143.79, 138.12, 131.91, 127.40 (2C), 126.68, 114.46 (2C), 108.53, 97.98, 79.55, 55.27, 53.36, 48.32, 47.35, 45.79, 44.38, 41.84, 28.40 (3C), 22.05, 12.24, 10.31. HPLC-MS (ESI): Purity = 96%, t_R = 2.659 min, *m/z* $[M+H]^+$ = 534.2.

***tert*-Butyl(2-((2-(cyclopropylmethyl)-1-(4-methoxybenzyl)-1*H*-imidazo[4,5-c]pyridin-6-yl)amino)ethyl)(ethyl)carbamate (e.8).** Obtained from intermediat**e c** (500 mg, 1.20 mmol) and 2-cyclopropylacetic acid (0.13 mL, 1.56 mmol) as a wine-colored sticky solid (96%, 552 mg); Rf (DCM:MeOH, 9:1) 0.57; ^{1}H-NMR (600 MHz, Chloroform-*d*) δ 8.34 (s, 1H), 6.96 (d, *J* = 8.7 Hz, 2H), 6.82–6.67 (m, 3H), 5.24 (s, 2H), 3.72 (s, 3H), 3.34–3.31 (m, 4H), 3.18 (q, *J* = 7.2 Hz, 2H), 2.69 (d, *J* = 6.8 Hz, 2H), 1.39 (s, 9H), 1.15–1.10 (m, 1H), 1.03 (t, *J* = 7.1 Hz, 3H), 0.57–0.53 (m, 2H), 0.52–0.47 (m, 2H). ^{13}C-NMR (151 MHz, $CDCl_3$) δ 178.61, 159.35, 154.07, 135.08, 127.77 (2C), 127.08, 114.36 (2C), 85.21, 79.42, 55.26, 53.38, 46.51, 45.79, 43.23, 41.68, 40.64, 32.38, 28.38 (3C), 14.02, 8.67, 7.24, 4.98, 4.37. HPLC-MS (ESI): Purity = 96%, t_R = 2.580 min, *m/z* $[M+H]^+$ = 480.3.

***tert*-Butyl(2-((2-cyclobutyl-1-(4-methoxybenzyl)-1*H*-imidazo[4,5-c]pyridin-6-yl)amino)ethyl)(ethyl)carbamate (e.9).** Obtained from intermediat**e c** (500 mg, 1.20 mmol) and cyclobutane carboxylic acid (0.15 mL, 1.56 mmol) as a wine-colored sticky solid (78%, 447 mg); Rf (DCM:MeOH, 9:1) 0.57; ^{1}H-NMR (600 MHz, Chloroform-*d*) δ 8.50 (s, 1H), 6.92 (d, *J* = 8.6 Hz, 2H), 6.77 (d, *J* = 8.6 Hz, 2H), 6.15 (s, 1H), 5.01 (s, 2H), 3.71 (s, 3H), 3.38–3.29 (m, 5H), 3.19 (q, *J* = 7.1 Hz, 2H), 2.52–2.43 (m, 2H), 2.28–2.22 (m, 2H), 2.04–1.88 (m, 2H), 1.38 (s, 9H), 1.02 (t, *J* = 7.1 Hz, 3H). ^{13}C-NMR (151 MHz, $CDCl_3$) δ 159.22, 158.00, 156.06, 154.76, 143.88, 139.08, 134.09, 128.46, 127.63 (2C), 127.45, 114.27 (2C), 84.66, 79.44, 55.19, 53.38, 46.05, 42.88, 42.11, 32.32, 28.38 (3C), 27.14, 18.53, 14.14. HPLC-MS (ESI): Purity = 96%, t_R = 2.571 min, *m/z* $[M+H]^+$ = 480.2.

***tert*-Butyl(2-((2-(3,3-difluorocyclobutyl)-1-(4-methoxybenzyl)-1*H*-imidazo[4,5-c]pyridin-6-yl)amino)ethyl)(ethyl)carbamate (e.10).** Obtained from intermediat**e c** (500 mg, 1.20 mmol) and 3,3-difluorocyclobutane-1-carboxylic acid (212 mg, 1.56 mmol) as a wine-colored sticky solid (79%, 491 mg); Rf (DCM:MeOH, 9:1) 0.57; ^{1}H-NMR (600 MHz, Chloroform-*d*) δ 8.35 (s, 1H), 6.98 (d, *J* = 8.7 Hz, 2H), 6.85–6.79 (m, 3H), 5.13 (s, 2H), 3.75 (s, 3H), 3.42–3.31 (m, 4H), 3.21 (q, *J* = 7.1 Hz, 2H), 3.07–2.96 (m, 1H), 2.84–2.73 (m, 4H), 1.39 (s, 9H), 1.05 (t, *J* = 7.1 Hz, 3H, H^{12}). ^{13}C-NMR (151 MHz, $CDCl_3$) δ 159.60, 156.04, 146.54, 134.04, 132.87, 127.71, 126.63 (2C), 120.47, 118.57, 116.80, 114.57 (2C), 85.67, 79.63, 55.28, 46.58, 45.69, 43.34, 41.71, 40.37, 39.03, 28.38 (3C), 21.83, 14.05. HPLC-MS (ESI): Purity = 98%, t_R = 2.589 min, *m/z* $[M+H]^+$ = 516.2.

***tert*-Butyl(2-((2-cyclohexyl-1-(4-methoxybenzyl)-1*H*-imidazo[4,5-c]pyridin-6-yl)amino)ethyl)(ethyl)carbamate (e.11).** Obtained from intermediat**e c** (500 mg, 1.20 mmol) and cyclohexane carboxylic acid (200 mg, 1.56 mmol) as a wine-colored sticky solid (68%, 412 mg); Rf (DCM:MeOH, 9:1) 0.57; ^{1}H-NMR (600 MHz, Chloroform-*d*) δ 8.19 (s, 1H), 7.43 (s, 1H), 7.03 (d, *J* = 8.7 Hz, 2H), 6.80 (d, *J* = 8.7 Hz, 2H), 5.21 (s, 2H), 3.73 (s, 3H), 3.38–3.27 (m, 4H), 3.19 (q, *J* = 7.1 Hz, 2H), 2.80–2.71 (m, 1H), 1.86–1.79 (m, 4H), 1.74–1.64 (m, 3H), 1.39 (s, 9H), 1.31–1.26 (m, 3H), 1.04 (t, *J* = 7.1 Hz, 3H). ^{13}C-NMR (151 MHz, $CDCl_3$) δ 164.23, 159.54, 155.97, 151.78, 147.27, 133.11, 129.45, 127.92 (2C), 126.60, 114.50 (2C), 86.72, 79.73, 55.26, 46.63, 45.47, 43.56, 41.32, 36.56, 31.49 (2C), 28.36 (3C), 25.98 (2C), 25.53, 14.12. HPLC-MS (ESI): Purity = 97%, t_R = 2.668 min, *m/z* $[M+H]^+$ = 508.3.

***tert*-Butyl(2-((2-(4,4-difluorocyclohexyl)-1-(4-methoxybenzyl)-1*H*-imidazo[4,5-c]pyridin-6-yl)amino)ethyl)(ethyl)carbamate (e.12).** Obtained from intermediat**e c** (500 mg, 1.20 mmol) and 4,4-difluorocyclohexane-1-carboxylic acid (256 mg, 1.56 mmol) as a wine-colored sticky solid (92%, 603 mg); Rf (DCM:MeOH, 9:1) 0.59; ^{1}H-NMR (600 MHz, Chloroform-*d*) δ 8.44 (s, 1H), 6.96 (d, *J* = 8.7 Hz, 2H), 6.82 (d, *J* = 8.7 Hz, 2H), 6.47 (s, 1H), 5.17 (s, 2H), 3.75 (s, 3H), 3.40–3.32 (m, 4H), 3.20 (q, *J* = 7.1 Hz, 2H), 2.84–2.77 (m, 1H), 2.24–2.18 (m, 2H), 2.11–2.01 (m, 2H), 1.91–1.83 (m, 2H), 1.83–1.68 (m, 2H), 1.40 (s, 9H), 1.05 (t, *J* = 7.1 Hz, 3H). ^{13}C-NMR (151 MHz, $CDCl_3$) δ 159.45, 157.88, 156.10, 154.48, 137.04, 133.57, 127.26 (2C), 123.94, 122.34, 120.74, 114.51 (2C), 85.17, 79.53, 55.28, 46.37, 45.94, 43.08, 41.99, 34.11, 33.17 (2C), 28.40 (3C), 27.68 (2C), 13.93. HPLC-MS (ESI): Purity = 97%, t_R = 2.615 min, *m/z* $[M+H]^+$ = 544.3.

***tert*-Butylethyl(2-((1-(4-methoxybenzyl)-2-(1-methylpyrrolidin-2-yl)-1*H*-imidazo[4,5-c]pyridin-6-yl)amino)ethyl)carbamate (e.13).** Obtained from intermediat**e c** (500 mg, 1.20 mmol) and 1-methylproline (202 mg, 1.56 mmol) as a wine-colored sticky solid (20%, 125 mg); Rf (DCM:MeOH, 9:1) 0.58; ^{1}H-NMR (600 MHz, Chloroform-*d*) δ 8.49 (s, 1H), 6.97 (d, *J* = 8.6 Hz, 2H), 6.80 (d, *J* = 8.6 Hz, 2H), 6.19 (s, 1H), 4.88 (s, 2H), 3.74 (s, 3H), 3.62 (dd, *J* = 8.2, 4.4 Hz, 1H), 3.38–3.32 (m, 4H), 3.18 (q, *J* = 7.1 Hz, 2H), 2.31–2.25 (m, 2H), 2.23 (s, 3H), 2.16–2.08 (m, 1H), 1.91–1.83 (m, 2H), 1.81–1.75 (m, 1H), 1.39 (s, 9H]), 1.05 (t, *J* = 7.1 Hz, 3H). ^{13}C-NMR (151 MHz, $CDCl_3$) δ 159.25, 159.05, 155.36, 154.72, 144.68, 133.93, 129.19, 128.09, 127.43 (2C), 114.17 (2C), 86.63, 79.57, 64.97, 56.55, 55.23, 46.72, 45.99, 44.03, 42.03, 40.79, 30.93, 28.40 (3C), 23.07, 13.86. HPLC-MS (ESI): Purity = 96%, t_R = 2.255 min, *m/z* $[M+H]^+$ = 509.3.

***tert*-Butylethyl(2-((1-(4-methoxybenzyl)-2-(3-(trifluoromethyl)phenyl)-1*H*-imidazo[4,5-c]pyridin-6-yl)amino)ethyl)carbamate (e.14).** Obtained from intermediat**e c** (500 mg, 1.20 mmol) and 3-(trifluoromethyl)benzoic acid (297 mg, 1.56 mmol) as a wine-colored sticky solid (70%, 481 mg); Rf (DCM:MeOH, 9:1) 0.59; ^{1}H-NMR (600 MHz, Chloroform-*d*) δ 8.49 (s, 1H), 8.36 (ddd, *J* = 1.6, 1.5, 0.8 Hz, 1H), 8.27 (ddd, *J* = 7.8, 1.6, 1.3 Hz, 1H), 7.92 (s, 1H), 7.73 (d, *J* = 8.7 Hz, 2H), 7.60 (ddd, *J* = 8.6, 1.5, 1.3 Hz, 1H), 7.53 (ddd, *J* = 8.6, 7.8, 0.8 Hz, 1H), 6.99 (d, *J* = 8.7 Hz, 2H), 5.32 (s, 2H), 3.76 (s, 3H), 3.42–3.39 (m, 4H), 3.24 (q, *J* = 7.1 Hz, 2H), 1.40 (s, 9H), 1.08 (t, *J* = 7.1 Hz, 3H). ^{13}C-NMR (151 MHz, $CDCl_3$) δ 170.61, 159.53, 134.48, 134.06, 132.87, 132.28, 129.89, 129.53, 128.59 (2C), 128.23, 127.75, 127.24, 126.86, 126.65, 126.21, 114.56 (2C), 86.37, 79.61, 55.85, 48.06, 45.50, 44.34, 43.44, 41.52, 28.37 (3C), 14.13. HPLC-MS (ESI): Purity = 98%, t_R = 2.712 min, *m/z* $[M+H]^+$ = 570.2.

***tert*-Butyl(2-((2-cycloheptyl-1-(4-methoxybenzyl)-1*H*-imidazo[4,5-c]pyridin-6-yl)amino)ethyl)(ethyl)carbamate (e.15).** Obtained from intermediat**e c** (500 mg, 1.20 mmol) and cycloheptane carboxylic acid (0.21 mL, 1.56 mmol) as a wine-colored sticky solid (37%, 232 mg); Rf (DCM:MeOH, 9:1) 0.53; ^{1}H-NMR (600 MHz, Chloroform-*d*) δ 8.30 (s, 1H), 7.02 (d, *J* = 8.7 Hz, 2H), 6.80 (d, *J* = 8.7 Hz, 2H), 6.66 (s, 1H), 5.16 (s, 2H), 3.74 (s, 3H), 3.40–3.38 (m, 1H), 3.36–3.28 (m, 4H), 3.19 (q, *J* = 7.1 Hz, 2H), 1.98–1.93 (m, 2H), 1.90–1.86 (m, 2H), 1.82–1.75 (m, 2H), 1.73–1.63 (m, 2H), 1.61–1.57 (m, 2H), 1.56–1.47 (m, 2H), 1.43 (s, 9H), 1.04 (t, *J* = 7.1 Hz, 3H). ^{13}C-NMR (151 MHz, $CDCl_3$) δ 181.97, 163.18, 162.53, 159.34, 155.88, 153.90, 134.43, 127.77 (2C), 127.34, 114.38 (2C), 85.36, 79.43, 55.25, 46.37, 45.49,

37.94, 33.36, 30.97 (2C), 28.39 (3C), 28.29, 27.91, 26.69, 26.45 (2C), 12.64. HPLC-MS (ESI): Purity = 98%, t_R = 2.791 min, *m/z* $[M+H]^+$ = 522.3.

***tert*-Butylethyl(2-((1-(4-methoxybenzyl)-2-((1r,4r)-4-(trifluoromethyl)cyclohexyl)-1*H*-imidazo[4,5-c]pyridin-6-yl)amino)ethyl)carbamate (e.16).** Obtained from intermediat**e c** (500 mg, 1.20 mmol) and (1r,4r)-4-(trifluoromethyl)cyclohexane-1-carboxylic acid (306 mg, 1.56 mmol) as a wine-colored sticky solid (27%, 184 mg); Rf (DCM:MeOH, 9:1) 0.64; ^{1}H-NMR (600 MHz, Chloroform-*d*) δ 8.45 (s, 1H), 6.96 (d, *J* = 8.7 Hz, 2H), 6.81 (d, *J* = 8.7 Hz, 2H), 6.41 (s, 1H), 5.15 (s, 2H), 3.75 (s, 3H), 3.37–3.35 (m, 5H), 3.21 (q, *J* = 7.1 Hz, 2H), 2.69 (tt, *J* = 11.9, 3.4 Hz, 1H), 2.05–2.00 (m, 2H), 1.95–1.90 (m, 2H), 1.81–1.72 (m, 2H), 1.40 (s, 9H), 1.36–1.30 (m, 2H), 1.04 (t, *J* = 7.1 Hz, 3H). ^{13}C-NMR (151 MHz, $CDCl_3$) δ 162.57, 159.40, 156.26, 154.60, 144.28, 137.61, 133.92, 128.40, 127.40 (2C), 126.55, 114.46 (2C), 85.11, 79.52, 55.26, 46.26, 45.98, 44.36, 42.02, 41.05, 38.15, 35.44, 30.00 (2C), 28.40 (3C), 24.68, 12.66. HPLC-MS (ESI): Purity = 98%, t_R = 2.703 min, *m/z* $[M+H]^+$ = 576.3.

***tert*-Butylethyl(2-((1-(4-methoxybenzyl)-2-((1r,4r)-4-(trifluoromethyl)cyclohexyl)-1*H*-imidazo[4,5-c]pyridin-6-yl)amino)ethyl)carbamate (e.17).** Obtained from intermediat**e c** (500 mg, 1.20 mmol) and (1s,4s)-4-(trifluoromethyl)cyclohexane-1-carboxylic acid (306 mg, 1.56 mmol) as a wine-colored sticky solid (34%, 234 mg); Rf (DCM: MeOH, 9:1) 0.53; ^{1}H-NMR (600 MHz, Chloroform-*d*) δ 8.29 (s, 1H), 6.93 (d, *J* = 8.7 Hz, 2H), 6.82 (d, *J* = 8.7 Hz, 2H), 6.43 (s, 1H), 5.21 (s, 2H), 3.75 (s, 3H), 3.42–3.29 (m, 5H), 3.21 (q, *J* = 7.1 Hz, 2H), 2.64 (tt, *J* = 11.8, 3.5 Hz, 1H), 2.25–2.22 (m, 2H), 2.14–2.10 (m, 2H), 2.01–1.97 (m, 2H), 1.79–1.75 (m, 2H), 1.43 (s, 9H), 1.05 (t, *J* = 7.1 Hz, 3H). ^{13}C-NMR (151 MHz, $CDCl_3$) δ 180.16, 178.97, 176.40, 159.54, 146.11, 132.66, 128.60, 127.77 (2C), 126.76, 114.51 (2C), 85.72, 79.53, 55.26, 46.48, 42.92, 41.15, 38.76, 35.67, 29.81, 28.37 (3C), 27.55, 25.76 (2C), 24.25, 21.75, 12.62. HPLC-MS (ESI): Purity = 97%, t_R = 2.703 min, *m/z* $[M+H]^+$ = 576.3.

***tert*-Butylethyl(2-((1-(4-methoxybenzyl)-2-(4-methoxycyclohexyl)-1*H*-imidazo[4,5-c]pyridin-6-yl)amino)ethyl)carbamate (e.18).** Obtained from intermediat**e c** (500 mg, 1.20 mmol) and 4-methoxycyclohexane-1-carboxylic acid (0.23 mL, 1.56 mmol) as a wine-colored sticky solid (15%, 99 mg); Rf (DCM:MeOH, 9:1) 0.45; ^{1}H-NMR (600 MHz, Chloroform-*d*) δ 8.27 (s, 1H), 7.03 (d, *J* = 8.7 Hz, 2H), 6.82 (d, *J* = 8.7 Hz, 2H), 6.41 (s, 1H), 5.20 (s, 2H), 3.75 (s, 3H), 3.41–3.39 (m, 1H), 3.35–3.32 (m, 4H), 3.28 (s, 3H), 3.22 (q, *J* = 7.2 Hz, 2H), 3.12 (tt, *J* = 10.6, 4.0 Hz, 1H), 2.20–2.14 (m, 2H), 1.79–1.74 (m, 2H), 1.68–1.64 (m, 2H), 1.40 (s, 9H),

1.24–1.21 (m, 2H), 1.09 (t, *J* = 7.2 Hz, 3H). ^{13}C-NMR (151 MHz, $CDCl_3$) δ 179.95, 179.68, 163.07, 159.50, 127.74 (2C), 126.96, 114.49 (2C), 79.58, 78.42, 75.15, 55.45, 44.33, 42.52, 41.64, 38.26, 31.51, 30.81, 29.71, 28.65 (2C), 28.39 (3C), 27.05, 25.51, 23.69 (2C), 12.66. HPLC-MS (ESI): Purity = 97%, t_R = 2.554 min, *m/z* $[M+H]^+$ = 538.3.

***tert*-Butylethyl(2-((1-(4-methoxybenzyl)-2-(6-(trifluoromethyl)pyridin-3-yl)-1*H*-imidazo[4,5-c]pyridin-6-yl)amino)ethyl)carbamate (e.19).** Obtained from intermediat**e c** (500 mg, 1.20 mmol) and 6-(trifluoromethyl)nicotinic acid (298 mg, 1.56 mmol) as a wine-colored sticky solid (18%, 121 mg); Rf (DCM:MeOH, 9:1) 0.53; ^{1}H-NMR (600 MHz, Chloroform-*d*) δ 8.95 (d, *J* = 2.1 Hz, 1H), 8.65 (s, 1H), 8.16 (d, *J* = 8.2 Hz, 1H), 7.73 (dd, *J* = 8.2, 2.1 Hz, 1H), 7.24 (s, 1H), 6.95 (d, *J* = 8.7 Hz, 2H), 6.82 (d, *J* = 8.7 Hz, 2H), 5.27 (s, 2H), 3.75 (s, 3H), 3.40–3.37 (m, 4H), 3.21 (q, *J* = 7.2 Hz, 2H), 1.43 (s, 9H), 1.05 (t, *J* = 7.1 Hz, 3H). ^{13}C-NMR (151 MHz, $CDCl_3$) δ 162.54, 159.49, 155.62, 149.42, 149.24, 144.61, 140.68, 137.86, 134.49, 128.93, 128.54, 127.05 (2C), 122.13, 120.38, 114.73 (2C), 113.88, 79.61, 64.84, 55.28, 47.84, 45.92, 44.37, 38.04, 28.36 (3C), 12.66. HPLC-MS (ESI): Purity = 98%, t_R = 2.668 min, *m/z* $[M+H]^+$ = 571.2.

5-chloro-2-((ethyl(2-((1-(4-methoxybenzyl)-2-methyl-1*H*-imidazo[4,5-c]pyridin-6-yl)amino)ethyl)amino)methyl)phenol (f.4). Obtained from intermediate **e.4** (70 mg, 0.21 mmol) and 4-chloro-2-hydroxybenzaldehyde (39 mg, 0.25 mmol) as a wine-colored sticky solid (65%, 66 mg); Rf (DCM:MeOH, 9:1) 0.63; ^{1}H-NMR (600 MHz, Chloroform-*d*) δ 8.35 (d, *J* = 1.0 Hz, 1H), 6.97 (d, *J* = 8.7 Hz, 2H), 6.86–6.81 (m, 3H), 6.74 (d, *J* = 2.1 Hz, 1H), 6.68 (dd, *J* = 8.0, 2.1 Hz, 1H), 6.10 (d, *J* = 1.0 Hz, 1H), 5.08 (s, 2H), 3.77 (s, 2H), 3.76 (s, 3H), 3.41 (t, *J* = 6.6 Hz, 2H), 2.78 (t, *J* = 6.5 Hz, 2H), 2.68 (q, *J* = 7.2 Hz, 2H), 2.46 (s, 3H), 1.08 (t, *J* = 7.1 Hz, 3H). ^{13}C-NMR (151 MHz, $CDCl_3$) δ 159.49, 158.68, 153.51, 153.41, 144.59, 136.35, 134.18, 133.92, 129.53, 127.68 (2C), 126.84, 120.08, 119.24, 116.47, 114.53 (2C), 85.45, 56.89, 55.30, 52.08, 47.88, 46.68, 40.48, 14.06, 10.85. HPLC-MS (ESI): Purity = 97%, t_R = 2.299 min, *m/z* $[M+H]^+$ = 480.2.

5-chloro-2-(((2-((2-cyclopropyl-1-(4-methoxybenzyl)-1*H*-imidazo[4,5-c]pyridin-6-yl)amino)ethyl)(ethyl)amino)methyl)phenol (f.5). Obtained from intermediate **e.5** (550 mg, 1.50 mmol) and 4-chloro-2-hydroxybenzaldehyde (282 mg, 1.80 mmol) as a wine-colored sticky solid (46%, 351 mg); Rf (DCM:MeOH, 9:1) 0.56; ^{1}H-NMR (600 MHz, Chloroform-*d*) δ 8.40 (d, *J* = 1.0 Hz, 1H), 7.03 (d, *J* = 8.7 Hz, 2H), 6.85–6.81 (m, 3H), 6.75 (d, *J* = 2.1 Hz,

1H), 6.68 (dd, *J* = 8.0, 2.1 Hz, 1H), 6.08 (d, *J* = 1.0 Hz, 1H), 5.22 (s, 2H), 3.75 (s, 3H), 3.75 (s, 2H), 3.42 (t, *J* = 6.5 Hz, 2H), 2.75 (t, *J* = 6.5 Hz, 2H), 2.64 (q, *J* = 7.2 Hz, 2H), 1.84 (tt, *J* = 8.2, 4.9 Hz, 1H), 1.16–1.13 (m, 2H), 1.05 (t, *J* = 7.2 Hz, 3H), 1.02–0.98 (m, 2H). ^{13}C-NMR (151 MHz, $CDCl_3$) δ 159.33, 158.81, 157.42, 153.93, 144.03, 138.03, 134.00, 133.98, 129.27, 127.69 (2C), 127.59, 120.40, 119.15, 116.44, 114.40 (2C), 85.15, 57.15, 55.28, 52.18, 47.65, 46.29, 40.48, 10.94, 8.20 (2C), 7.71. HPLC-MS (ESI): Purity = 98%, t_R = 2.352 min, *m/z* $[M+H]^+$ = 506.2.

5-chloro-2-((ethyl(2-((1-(4-methoxybenzyl)-2-(1-methylcyclopropyl)-1*H*-imidazo[4,5-c]pyridin-6-yl)amino)ethyl)amino)methyl)phenol (f.6). Obtained from intermediate **e.6** (440 mg, 1.16 mmol) and 4-chloro-2-hydroxybenzaldehyde (272 mg, 1.74 mmol) as an orange-colored sticky solid (63%, 381 mg); Rf (DCM:MeOH, 9:1) 0.52; ^{1}H-NMR (600 MHz, Chloroform-*d*) δ 8.44 (d, *J* = 1.0 Hz, 1H), 6.95 (d, *J* = 8.7 Hz, 2H), 6.84–6.80 (m, 3H), 6.74 (d, *J* = 2.1 Hz, 1H), 6.68 (dd, *J* = 8.0, 2.1 Hz, 1H), 5.89 (d, *J* = 1.0 Hz, 1H), 5.33 (s, 2H), 3.75 (s, 3H), 3.72 (s, 2H), 3.36 (t, *J* = 6.5 Hz, 2H), 2.71 (t, *J* = 6.5 Hz, 2H), 2.61 (q, *J* = 7.1 Hz, 2H), 1.35 (s, 3H), 1.10–1.07 (m, 2H), 1.03 (t, *J* = 7.2 Hz, 3H), 0.80–0.77 (m, 2H). ^{13}C-NMR (151 MHz, $CDCl_3$) δ 159.21, 158.78, 158.62, 154.05, 143.98, 138.70, 134.01, 129.27, 127.33 (2C), 127.27, 120.34, 119.12, 116.44, 114.42, 114.38 (2C), 85.79, 57.08, 55.26, 52.09, 47.64, 46.83, 40.35, 23.73, 14.16, 13.33 (2C), 10.90. HPLC-MS (ESI): Purity = 98%, t_R = 2.571 min, *m/z* $[M+H]^+$ = 520.2.

5-chloro-2-((ethyl(2-((1-(4-methoxybenzyl)-2-(1-(trifluoromethyl)cyclopropyl)-1*H*-imidazo[4,5-c]pyridin-6-yl)amino)ethyl)amino)methyl)phenol (f.7). Obtained from intermediate **e.7** (89 mg, 0.21 mmol) and 4-chloro-2-hydroxybenzaldehyde (50 mg, 0.32 mmol) as an orange-colored sticky solid (49%, 59 mg); Rf (DCM:MeOH, 9:1) 0.56; ^{1}H-NMR (600 MHz, Chloroform-*d*) δ 8.49 (d, *J* = 1.0 Hz, 1H), 6.90 (d, *J* = 8.7 Hz, 2H), 6.84–6.80 (m, 3H), 6.75 (d, *J* = 2.1 Hz, 1H), 6.68 (dd, *J* = 8.0, 2.1 Hz, 1H), 5.88 (d, *J* = 1.0 Hz, 1H), 4.78 (s, 2H), 3.75 (s, 3H), 3.74 (s, 2H), 3.35 (t, *J* = 6.3 Hz, 2H), 2.75 (t, *J* = 6.3 Hz, 2H), 2.67 (q, *J* = 7.2 Hz, 2H), 1.50–1.47 (m, 2H), 1.11–1.09 (m, 2H), 1.07 (t, *J* = 7.2 Hz, 3H). ^{13}C-NMR (151 MHz, $CDCl_3$) δ 159.35, 158.51, 157.05, 154.35, 143.80, 139.59, 129.69, 128.58, 127.40, 126.59 (2C), 123.88, 119.77, 119.30, 116.71, 114.47 (2C), 86.22, 72.27, 71.12, 63.56, 61.68, 55.27, 52.18, 42.84, 40.11, 10.68 (2C), 10.29. HPLC-MS (ESI): Purity = 97%, t_R = 2.483 min, *m/z* $[M+H]^+$ = 574.2.

5-chloro-2-(((2-((2-(cyclopropylmethyl)-1-(4-methoxybenzyl)-1*H*-imidazo[4,5-c]pyridin-6-yl)amino)ethyl)(ethyl)amino)methyl)phenol (f.8). Obtained from intermediate **e.8** (270 mg, 0.71 mmol) and 4-chloro-2-hydroxybenzaldehyde (168 mg, 1.07 mmol) as a wine-colored sticky solid (65%, 239 mg); Rf (DCM:MeOH, 9:1) 0.59; ^{1}H-NMR (600 MHz, Chloroform-*d*) δ 8.50 (d, *J* = 1.0 Hz, 1H), 6.94 (d, *J* = 8.7 Hz, 2H), 6.83–6.78 (m, 3H), 6.74 (d, *J* = 2.1 Hz, 1H), 6.67 (dd, *J* = 8.0, 2.1 Hz, 1H), 6.04 (d, *J* = 1.0 Hz, 1H), 5.11 (s, 2H), 3.74 (s, 3H), 3.73 (s, 2H), 3.41 (t, *J* = 6.5 Hz, 2H), 2.74 (t, *J* = 6.5 Hz, 2H), 2.67 (d, *J* = 6.8 Hz, 2H), 2.63 (q, *J* = 7.2 Hz, 2H), 1.15–1.10 (m, 1H), 1.04 (t, *J* = 7.2 Hz, 3H), 0.57–0.53 (m, 2H), 0.21–0.17 (m, 2H). ^{13}C-NMR (151 MHz, $CDCl_3$) δ 159.31, 158.82, 155.31, 154.21, 143.65, 138.98, 134.32, 133.97, 129.26, 127.45 (2C), 127.35, 120.42, 119.12, 116.41, 114.40 (2C), 85.31, 57.13, 55.27, 52.17, 47.60, 46.36, 40.44, 32.41, 10.92, 8.75, 4.98 (2C). HPLC-MS (ESI): Purity = 98%, t_R = 2.598 min, *m/z* $[M+H]^+$ = 520.2.

5-chloro-2-(((2-((2-cyclobutyl-1-(4-methoxybenzyl)-1*H*-imidazo[4,5-c]pyridin-6-yl)amino)ethyl)(ethyl)amino)methyl)phenol (f.9). Obtained from intermediate **e.9** (400 mg, 1.05 mmol) and 4-chloro-2-hydroxybenzaldehyde (247 mg, 1.58 mmol) as an orange-colored sticky solid (57%, 311 mg); Rf (DCM:MeOH, 9:1) 0.53; ^{1}H-NMR (400 MHz, Chloroform-*d*) δ 8.55 (d, *J* = 1.0 Hz, 1H), 6.96 (d, *J* = 8.7 Hz, 2H), 6.87–6.82 (m, 3H), 6.78 (d, *J* = 2.1 Hz, 1H), 6.71 (dd, *J* = 8.0, 2.1 Hz, 1H), 6.07 (d, *J* = 1.0 Hz, 1H), 5.05 (s, 2H), 3.78 (s, 3H), 3.77 (s, 2H), 3.65–3.54 (m, 1H), 3.45 (t, *J* = 6.5 Hz, 2H), 2.78 (t, *J* = 6.5 Hz, 2H), 2.66 (q, *J* = 7.1 Hz, 2H), 2.56–2.47 (m, 2H), 2.35–2.26 (m, 2H), 2.11–1.95 (m, 2H), 1.08 (t, *J* = 7.2 Hz, 3H). ^{13}C-NMR (101 MHz, $CDCl_3$) δ 159.34, 158.87, 158.40, 154.24, 143.96, 138.91, 134.34, 133.99, 129.24, 127.56, 127.46 (2C), 120.48, 119.12, 116.45, 114.40 (2C), 85.26, 57.20, 55.28, 52.28, 47.64, 46.22, 40.49, 32.39, 27.16 (2C), 18.57, 10.96. HPLC-MS (ESI): Purity = 98%, t_R = 2.624 min, *m/z* $[M+H]^+$ = 520.2.

5-chloro-2-(((2-((2-(3,3-difluorocyclobutyl)-1-(4-methoxybenzyl)-1*H*-imidazo[4,5-c]pyridin-6-yl)amino)ethyl)(ethyl)amino)methyl)phenol (f.10). Obtained from intermediate **e.10** (350 mg, 0.84 mmol) and 4-chloro-2-hydroxybenzaldehyde (197 mg, 1.26 mmol) as a wine-colored sticky solid (59%, 274 mg); Rf (DCM:MeOH, 9:1) 0.49; ^{1}H-NMR (600 MHz, Chloroform-*d*) δ 8.51 (d, *J* = 1.0 Hz, 1H), 6.91 (d, *J* = 8.7 Hz, 2H), 6.83–6.80 (m, 3H), 6.72 (d, *J* = 2.1 Hz, 1H), 6.67 (dd, *J* = 8.0, 2.1 Hz, 1H), 6.08 (d, *J* = 1.0 Hz, 1H), 5.05 (s, 2H), 3.75 (s, 3H), 3.74 (s, 2H), 3.43 (t, *J* = 6.5 Hz, 2H), 3.36–3.28 (m, 1H), 3.07–2.95 (m, 4H), 2.75 (t, *J* = 6.4 Hz, 3H), 2.64 (q, *J* = 7.2 Hz, 2H), 1.05 (t, *J* = 7.2 Hz, 3H). ^{13}C-NMR (151 MHz, $CDCl_3$) δ

159.49, 158.78, 155.33, 154.58, 144.18, 139.48, 133.99, 133.84, 129.29, 127.40 (2C), 127.09, 120.38, 119.15, 116.41, 114.54 (2C), 84.97, 57.12, 55.29, 52.17, 47.65, 46.29, 40.52, 40.40, 40.21, 20.95 (2C), 10.93. HPLC-MS (ESI): Purity = 97%, t_R = 2.580 min, *m/z* $[M+H]^+$ = 556.2.

5-chloro-2-(((2-((2-cyclohexyl-1-(4-methoxybenzyl)-1*H*-imidazo[4,5-c]pyridin-6-yl)amino)ethyl)(ethyl)amino)methyl)phenol (f.11). Obtained from intermediate **e.11** (220 mg, 0.54 mmol) and 4-chloro-2-hydroxybenzaldehyde (127 mg, 0.81 mmol) as a wine-colored sticky solid (93%, 274 mg); Rf (DCM:MeOH, 9:1) 0.51; ^{1}H-NMR (400 MHz, Chloroform-*d*) δ 8.52 (d, *J* = 0.9 Hz, 1H), 6.99 (d, *J* = 8.8 Hz, 2H), 6.89–6.85 (m, 3H), 6.79 (d, *J* = 2.1 Hz, 1H), 6.72 (dd, *J* = 8.0, 2.1 Hz, 1H), 6.08 (d, *J* = 1.0 Hz, 1H), 5.17 (s, 2H), 3.80 (s, 3H), 3.79 (s, 2H), 3.46 (t, *J* = 6.5 Hz, 2H), 2.79 (t, *J* = 6.5 Hz, 2H), 2.76–2.71 (m, 1H), 2.68 (q, *J* = 7.1 Hz, 2H), 1.90–1.85 (m, 4H), 1.79–1.72 (m, 3H), 1.36–1.31 (m, 3H), 1.10 (t, *J* = 7.1 Hz, 3H). ^{13}C-NMR (101 MHz, $CDCl_3$) δ 160.08, 159.39, 158.85, 153.91, 143.72, 138.23, 134.35, 134.05, 129.28, 127.56, 127.47 (2C), 120.43, 119.16, 116.48, 114.48 (2C), 85.55, 57.19, 55.30, 52.25, 47.73, 46.27, 40.53, 36.45, 31.74 (2C), 26.21 (2C), 25.71, 10.95. HPLC-MS (ESI): Purity = 97%, t_R = 2.685 min, *m/z* $[M+H]^+$ = 548.2.

5-chloro-2-(((2-((2-(4,4-difluorocyclohexyl)-1-(4-methoxybenzyl)-1*H*-imidazo[4,5-c]pyridin-6-yl)amino)ethyl)(ethyl)amino)methyl)phenol (f.12). Obtained from intermediate **e.12** (480 mg, 1.08 mmol) and 4-chloro-2-hydroxybenzaldehyde (254 mg, 1.62 mmol) as a wine-colored sticky solid (60%, 381 mg); Rf (DCM:MeOH, 9:1) 0.55; ^{1}H-NMR (400 MHz, Chloroform-*d*) δ 8.54 (d, *J* = 1.0 Hz, 1H), 6.97 (d, *J* = 8.7 Hz, 2H), 6.87–6.83 (m, 3H), 6.76 (d, *J* = 2.1 Hz, 1H), 6.70 (dd, *J* = 8.0, 2.1 Hz, 1H), 6.10 (d, *J* = 1.0 Hz, 1H), 5.16 (s, 2H), 3.78 (s, 3H, H^8), 3.77 (s, 2H), 3.46 (t, *J* = 6.5 Hz, 2H), 2.85–2.80 (m, 1H), 2.77 (t, *J* = 6.5 Hz, 2H), 2.66 (q, *J* = 7.2 Hz, 2H), 2.25–2.20 (m, 2H), 2.14–2.02 (m, 2H), 1.92–1.65 (m, 4H), 1.08 (t, *J* = 7.2 Hz, 3H). ^{13}C-NMR (101 MHz, $CDCl_3$) δ 159.48, 158.86, 157.04, 154.49, 143.46, 139.39, 134.19, 133.96, 129.27, 127.45, 127.36 (2C), 120.51, 119.12, 116.42, 114.56 (2C), 85.27, 57.17, 55.30, 52.27, 47.64, 46.30, 40.47, 34.08, 33.44, 33.20 (2C), 27.80 (2C), 10.94. HPLC-MS (ESI): Purity = 98%, t_R = 2.589 min, *m/z* $[M+H]^+$ = 584.2.

5-chloro-2-((ethyl(2-((1-(4-methoxybenzyl)-2-(1-methylpyrrolidin-2-yl)-1*H*-imidazo[4,5-c]pyridin-6-yl)amino)ethyl)amino)methyl)phenol (f.13). Obtained from intermediate **e.13** (100 mg, 0.24 mmol) and 4-chloro-2-hydroxybenzaldehyde (56 mg, 0.36 mmol) as a yellow sticky solid (39%, 51 mg); Rf (DCM:MeOH, 9:1) 0.47; ^{1}H-NMR (400 MHz, Chloroform-*d*) δ

8.52 (d, *J* = 1.0 Hz, 1H), 7.01 (d, *J* = 8.6 Hz, 2H), 6.87–6.84 (m, 3H), 6.79 (d, *J* = 2.1 Hz, 1H), 6.72 (dd, *J* = 8.0, 2.2 Hz, 1H), 6.05 (d, *J* = 1.0 Hz, 1H), 4.90 (s, 2H), 3.80 (s, 3H), 3.79 (s, 2H), 3.43 (t, *J* = 6.5 Hz, 2H), 3.23 (t, *J* = 8.1 Hz, 1H), 2.79 (t, *J* = 6.4 Hz, 2H), 2.68 (q, *J* = 7.1 Hz, 2H), 2.40–2.32 (m, 1H), 2.29 (s, 3H), 2.21–2.13 (m, 1H), 1.99–1.81 (m, 4H), 1.10 (t, *J* = 7.1 Hz, 3H). ^{13}C-NMR (101 MHz, $CDCl_3$) δ 159.21, 158.81, 154.12, 144.73, 138.77, 134.10, 129.31, 128.91, 127.46 (2C), 120.33, 119.17, 116.49, 114.31 (2C), 85.63, 64.77, 57.12, 56.52, 55.28, 52.26, 47.74, 46.81, 44.11, 40.70, 40.03, 30.99, 25.35, 23.10, 10.91. HPLC-MS (ESI): Purity = 97%, t_R = 2.027 min, *m/z* $[M+H]^+$ = 549.2.

5-chloro-2-((ethyl(2-((1-(4-methoxybenzyl)-2-(3-(trifluoromethyl)phenyl)-1*H*-imidazo[4,5-c]pyridin-6-yl)amino)ethyl)amino)methyl)phenol (f.14). Obtained from intermediate **e.14** (420 mg, 0.89 mmol) and 4-chloro-2-hydroxybenzaldehyde (210 mg, 1.34 mmol) as a wine-colored sticky solid (42%, 231 mg); Rf (DCM:MeOH, 9:1) 0.50 ; ^{1}H-NMR (400 MHz, Chloroform-*d*) δ 8.66 (d, *J* = 1.0 Hz, 1H), 7.95 (dd, *J* = 2.1, 1.5 Hz, 1H), 7.83 (ddd, *J* = 7.9, 2.1, 1.6 Hz, 1H), 7.75 (ddd, *J* = 7.7, 1.6, 1.5 Hz, 1H), 7.59 (dd, *J* = 7.9, 7.7 Hz, 1H), 7.01 (d, *J* = 8.7 Hz, 2H), 6.91–6.86 (m, 3H), 6.80 (d, *J* = 2.1 Hz, 1H), 6.73 (dd, *J* = 8.0, 2.1 Hz, 1H), 6.18 (d, *J* = 1.0 Hz, 1H), 5.25 (s, 2H), 3.83 (s, 2H), 3.81 (s, 3H), 3.52 (t, *J* = 6.4 Hz, 2H), 2.86 (t, *J* = 6.4 Hz, 2H), 2.74 (q, *J* = 7.1 Hz, 2H), 1.14 (t, *J* = 7.2 Hz, 3H). ^{13}C-NMR (101 MHz, $CDCl_3$) δ 159.50, 158.71, 156.41, 154.58, 152.85, 144.62, 139.85, 134.60, 134.25, 132.16, 131.60, 131.27, 130.53, 129.53, 129.32, 127.33 (2C), 126.69, 126.17, 124.96, 120.08, 119.28, 116.58, 114.67 (2C), 85.74, 56.97, 55.32, 52.27, 47.92, 40.32, 10.86. HPLC-MS (ESI): Purity = 98%, t_R = 2.677 min, *m/z* $[M+H]^+$ = 610.2.

5-chloro-2-(((2-((2-cycloheptyl-1-(4-methoxybenzyl)-1*H*-imidazo[4,5-c]pyridin-6-yl)amino)ethyl)(ethyl)amino)methyl)phenol (f.15). Obtained from intermediate **e.15** (150 mg, 0.36 mmol) and 4-chloro-2-hydroxybenzaldehyde (85 mg, 0.54 mmol) as a wine-colored sticky solid (37%, 76 mg); Rf (DCM:MeOH, 9:1); ^{1}H-NMR (600 MHz, Chloroform-*d*) δ 8.45 (d, *J* = 1.0 Hz, 1H), 6.95 (d, *J* = 8.7 Hz, 2H), 6.83–6.81 (m, 3H), 6.74 (d, *J* = 2.1 Hz, 1H), 6.67 (dd, *J* = 8.0, 2.1 Hz, 1H), 6.04 (d, *J* = 1.0 Hz, 1H), 5.11 (s, 2H), 3.75 (s, 3H), 3.74 (s, 2H), 3.41 (t, *J* = 6.6 Hz, 2H), 2.92–2.85 (m, 1H), 2.75 (t, *J* = 6.5 Hz, 2H), 2.64 (q, *J* = 7.1 Hz, 2H), 1.91–1.86 (m, 4H), 1.82–1.76 (m, 2H), 1.60 (dd, *J* = 5.3, 3.3 Hz, 4H), 1.44–1.41 (m, 2H), 1.05 (t, *J* = 7.2 Hz, 3H, H^{12}). ^{13}C-NMR (151 MHz, $CDCl_3$) δ 161.20, 159.38, 158.83, 153.94, 143.74, 138.12, 134.25, 134.02, 129.23, 127.55, 127.47 (2C), 120.41, 119.12, 116.45, 114.46 (2C),

85.43, 57.17, 55.27, 52.22, 47.70, 46.30, 40.54, 37.91, 33.45 (2C), 27.96 (2C), 26.70 (2C), 10.91. HPLC-MS (ESI): Purity = 98%, t_R = 2.729 min, *m/z* $[M+H]^+$ = 562.2.

5-chloro-2-((ethyl(2-((1-(4-methoxybenzyl)-2-((1r,4r)-4-(trifluoromethyl)cyclohexyl)-1*H*-imidazo[4,5-c]pyridin-6-yl)amino)ethyl)amino)methyl)phenol (f.16). Obtained from intermediate **e.16** (150 mg, 0.32 mmol) and 4-chloro-2-hydroxybenzaldehyde (75 mg, 0.48 mmol) as a wine-colored sticky solid (41%, 81 mg); Rf (DCM:MeOH, 9:1) 0.50; ^{1}H-NMR (600 MHz, Chloroform-*d*) δ 8.47 (d, *J* = 1.0 Hz, 1H), 6.94 (d, *J* = 8.7 Hz, 2H), 6.84–6.81 (m, 3H), 6.73 (d, *J* = 2.2 Hz, 1H), 6.67 (dd, *J* = 8.0, 2.3 Hz, 1H), 6.06 (d, *J* = 1.0 Hz, 1H), 5.12 (s, 2H), 3.75 (s, 3H), 3.74 (s, 2H), 3.42 (t, *J* = 6.5 Hz, 2H), 2.75 (t, *J* = 6.5 Hz, 2H), 2.70–2.67 (m, 1H), 2.65 (q, *J* = 7.2 Hz, 2H), 2.14–2.06 (m, 1H), 2.04–2.01 (m, 2H), 1.94–1.89 (m, 2H), 1.80–1.72 (m, 2H), 1.38–1.30 (m, 2H), 1.05 (t, *J* = 7.2 Hz, 3H). ^{13}C-NMR (151 MHz, $CDCl_3$) δ 159.49, 158.79, 158.44, 154.22, 143.60, 138.69, 134.13, 134.05, 129.27, 127.39 (2C), 120.36, 119.15, 116.44, 114.55 (2C), 85.36, 57.13, 55.27, 52.26, 47.72, 46.26, 41.93, 41.87, 41.27, 41.09, 40.48, 35.47, 30.00 (2C), 24.70, 10.89. HPLC-MS (ESI): Purity = 97%, t_R = 2.694 min, *m/z* $[M+H]^+$ = 616.2.

5-chloro-2-((ethyl(2-((1-(4-methoxybenzyl)-2-((1r,4r)-4-(trifluoromethyl)cyclohexyl)-1*H*-imidazo[4,5-c]pyridin-6-yl)amino)ethyl)amino)methyl)phenol (f.17). Obtained from intermediate **e.17** (170 mg, 0.36 mmol) and 4-chloro-2-hydroxybenzaldehyde (85 mg, 0.54 mmol) as a wine-colored sticky solid (35%, 77 mg); Rf (DCM:MeOH, 9:1) 0.63; ^{1}H-NMR (600 MHz, Chloroform-*d*) δ 8.47 (d, *J* = 1.0 Hz, 1H), 6.94 (d, *J* = 8.7 Hz, 2H), 6.83–6.81 (m, 3H), 6.73 (d, *J* = 2.1 Hz, 1H), 6.67 (dd, *J* = 8.0, 2.1 Hz, 1H), 6.06 (d, *J* = 1.0 Hz, 1H), 5.12 (s, 2H), 3.75 (s, 3H), 3.75 (s, 2H), 3.42 (t, *J* = 6.5 Hz, 2H), 2.76 (t, *J* = 6.5 Hz, 2H), 2.70–2.67 (m, 1H), 2.65 (q, *J* = 7.2 Hz, 3H), 2.13–2.07 (m, 1H), 2.04–2.01 (m, 2H), 1.94–1.89 (m, 2H), 1.80–1.72 (m, 2H), 1.38–1.30 (m, 2H), 1.06 (t, *J* = 7.2 Hz, 3H). ^{13}C-NMR (151 MHz, $CDCl_3$) δ 159.44, 158.78, 158.52, 154.20, 143.67, 138.51, 134.04, 134.02, 129.32, 127.39 (2C), 127.19, 120.34, 119.14, 116.42, 114.51 (2C), 85.28, 57.08, 55.29, 52.10, 47.69, 46.36, 46.24, 41.06, 40.44, 35.44, 29.99 (2C), 27.40, 24.69, 10.89. HPLC-MS (ESI): Purity = 98%, t_R = 2.878 min, *m/z* $[M+H]^+$ = 616.2.

5-chloro-2-((ethyl(2-((1-(4-methoxybenzyl)-2-(4-methoxycyclohexyl)-1*H*-imidazo[4,5-c]pyridin-6-yl)amino)ethyl)amino)methyl)phenol (f.18). Obtained from intermediate **e.18** (80 mg, 0.18 mmol) and 4-chloro-2-hydroxybenzaldehyde (42 mg, 0.27 mmol) as an orange-

colored sticky solid (70%, 73 mg); Rf (DCM:MeOH, 9:1) 0.47; ^{1}H-NMR (600 MHz, Chloroform-*d*) δ 8.42 (d, *J* = 1.0 Hz, 1H), 6.95 (d, *J* = 8.7 Hz, 2H), 6.85–6.81 (m, 3H), 6.74 (d, *J* = 2.1 Hz, 1H), 6.67 (dd, *J* = 8.0, 2.1 Hz, 1H), 6.07 (d, *J* = 1.0 Hz, 1H), 5.13 (s, 2H), 3.77 (s, 3H), 3.76 (s, 2H), 3.47 (tt, *J* = 11.3, 4.3 Hz, 1H), 3.42 (t, *J* = 6.4 Hz, 2H), 3.34 (s, 3H), 3.19 (tt, *J* = 10.7, 4.2 Hz, 1H), 2.78 (t, *J* = 6.4 Hz, 2H), 2.68 (q, *J* = 7.2 Hz, 2H), 2.18–2.13 (m, 2H), 1.90–1.84 (m, 2H), 1.81–1.72 (m, 2H), 1.25–1.21 (m, 2H), 1.08 (t, *J* = 7.2 Hz, 3H). ^{13}C-NMR (151 MHz, $CDCl_3$) δ 159.47, 158.69, 153.50, 144.28, 136.87, 134.14, 129.50, 127.52 (2C), 127.40, 119.22, 116.44, 114.53 (2C), 85.54, 78.32, 73.57, 56.94, 55.77, 55.30, 52.01, 47.89, 46.39, 40.43, 35.67, 31.57 (2C), 29.79 (2C), 29.00, 25.57, 10.87. HPLC-MS (ESI): Purity = 97%, t_R = 2.650 min, *m/z* $[M+H]^+$ = 578.2.

5-chloro-2-((ethyl(2-((1-(4-methoxybenzyl)-2-(6-(trifluoromethyl)pyridin-3-yl)-1*H*-imidazo[4,5-c]pyridin-6-yl)amino)ethyl)amino)methyl)phenol (f.19). Obtained from intermediate **e.19** (87 mg, 0.18 mmol) and 4-chloro-2-hydroxybenzaldehyde (42 mg, 0.27 mmol) as a wine-colored sticky solid (50%, 55 mg); Rf (DCM:MeOH, 9:1) 0.58; ^{1}H-NMR (600 MHz, Chloroform-*d*) δ 8.95 (d, *J* = 1.0 Hz, 1H), 8.63 (d, *J* = 1.8 Hz, 1H), 8.15 (d, *J* = 8.2 Hz, 1H), 7.73 (dd, *J* = 8.2, 1.8 Hz, 1H), 6.95 (d, *J* = 8.7 Hz, 2H), 6.86–6.83 (m, 3H), 6.73 (d, *J* = 2.1 Hz, 1H), 6.68 (dd, *J* = 8.0, 2.1 Hz, 1H), 6.12 (d, *J* = 1.0 Hz, 1H), 5.24 (s, 2H), 3.78 (s, 2H), 3.76 (s, 3H), 3.47 (t, *J* = 6.3 Hz, 2H), 2.81 (t, *J* = 6.3 Hz, 2H), 2.70 (q, *J* = 7.2 Hz, 2H), 1.08 (t, *J* = 7.2 Hz, 3H). ^{13}C-NMR (151 MHz, $CDCl_3$) δ 159.54, 158.60, 155.00, 149.46, 144.49, 140.74, 137.88, 134.69, 134.25, 129.57, 128.82, 127.04 (2C), 126.84, 120.39, 120.37, 119.88, 119.26, 116.50, 114.79 (2C), 85.39, 56.84, 55.31, 53.23, 52.20, 47.84, 41.84, 38.57, 12.62, 10.77. HPLC-MS (ESI): Purity = 98%, t_R = 2.975 min, *m/z* $[M+H]^+$ = 611.1.

Synthesis and characterization of Imidazopyridine Fluorescent Probe (14-NBD)

Procedure for the synthesis of intermediate a (Scheme 2)

A mixture of p-methoxybenzyl amine and *N*,*N*-Diisopropylethylamine (DIPEA) in tetrahydrofuran (THF) was added dropwise to a 0 °C solution of 2,4-dichloro-5-nitropyridine in THF. The solution was then warmed up to 25 °C and stirred for an additional 30 min. Water was then added, and the resulting mixture was extracted with ethyl acetate. The combined organic layer was dried over anhydrous Na_2SO_4 and concentrated under reduced pressure to produce the desired intermediate as a yellow solid in 98% yield.

2-chloro-*N*-(4-methoxybenzyl)-5-nitropyridin-4-amine (a). Obtained from 2,4-dichloro-5-nitropyridine (2.00 g, 10.36 mmol) and p-methoxybenzyl amine (2.56 g, 18.65 mmol) as a yellow solid (98%, 3.54 g); Rf (Hex: EtOAc, 1:1) 0.54; ^{1}H-NMR (600 MHz, DMSO-d_6) δ 8.96 (t, J = 6.1 Hz, 1H), 8.85 (s, 1H), 7.29 (d, J = 8.7 Hz, 2H), 6.94 (s, 1H), 6.89 (d, J = 8.7 Hz, 2H), 4.57 (d, J = 6.1 Hz, 2H), 3.71 (s, 3H). ^{13}C-NMR (151 MHz, DMSO) δ 159.91, 155.92, 150.57, 150.08, 130.29, 129.76 (2C), 115.42 (2C), 108.83, 56.41, 46.26. HPLC-MS (ESI): Purity = 98%, t_R = 2.457 min, *m/z* $[M-H]^+$ = 294.0.

Procedure for the synthesis of intermediate g (Scheme 2)

A mixture of 2-chloro-*N*-(4-methoxybenzyl)-5-nitropyridin-4-amine (**a**), *N*-boc ethylenediamine, and triethylamine was made in *N*,*N*-dimethylformamide (DMF). The mixture was heated under microwave radiation at 100 °C for 1 hour. When the reaction had completed, water was added, and the mixture was extracted with ethyl acetate (4 × 30 mL). The combined organic layer was dried over anhydrous Na_2SO_4, concentrated under reduced pressure, and purified via column chromatography. A yellow solid was obtained as the product.

***tert*-Butyl (2-((4-((4-methoxybenzyl)amino)-5-nitropyridin-2-yl)amino)ethyl)carbamate (g).** Obtained from intermediate **a** (3.50 g, 11.92 mmols) and *N*-boc ethylenediamine (2.26 mL, 14.30 mmols) as a yellow solid (96%, 4.78 g); R_f (DCM: MeOH, 9:1) 0.68; ^{1}H NMR (600 MHz, MeOH-d_4) δ 8.84 (s, 1H), 7.31 (d, J = 8.3 Hz, 2H), 6.93 (d, J = 8.4 Hz, 2H), 5.67 (s, 1H), 4.46 (s, 2H), 3.79 (s, 3H), 3.33 (t, J = 6.3 Hz, 2H), 3.20 (t, J = 6.3 Hz, 2H), 1.43 (s, 9H). ^{13}C NMR (151 MHz, MeOD) δ 161.46, 159.24, 157.20, 150.04, 149.55, 129.35, 128.20 (2C), 124.25, 113.86 (2C), 84.12, 78.77, 54.33, 45.37, 41.45, 39.31, 27.35 (3C). HPLC-MS (ESI): Purity = 97%, t_R = 2.598 min, m/z $[M+H]^+$ = 418.2.

Procedure for the synthesis of intermediate h (Scheme 2)

A mixture of *tert*-butyl (2-((4-((4-methoxybenzyl)amino)-5-nitropyridin-2-yl)amino)ethyl)carbamate (**g**), Zinc and acetic acid in DCM was stirred for 30 minutes at 25 °C. After the reaction had been completed, the mixture was filtered through a pad of celite and concentrated *in vacuo* to obtain the product, which was used in the next reaction without any further purification.

***tert*-Butyl (2-((5-amino-4-((4-methoxybenzyl)amino)pyridin-2-yl)amino)ethyl)carbamate (h).** Obtained from intermediate **g** (3.30 g, 7.90 mmols) as a wine-coloured solid (92%, 2.81

g); R_f (DCM: MeOH, 9:1) 0.40; ^{1}H NMR (600 MHz, MeOH-d_4) δ 7.32 (d, J = 8.2 Hz, 2H), 7.24 (s, 1H), 6.90 (d, J = 8.3 Hz, 2H), 5.75 (s, 1H), 4.41 (s, 2H), 3.78 (s, 3H), 3.16 – 3.13 (m, 4H), 1.44 (s, 9H). ^{13}C NMR (151 MHz, MeOD) δ 159.12, 158.86, 157.13, 149.76, 129.81, 128.34 (2C), 128.06, 113.71 (2C), 113.42, 86.01, 78.84, 54.34, 45.61, 41.99, 39.20, 27.38 (3C). HPLC-MS (ESI): Purity = 97%, t_R = 2.246 min, m/z $[M+H]^+$ = 388.2.

Procedure for the synthesis of intermediates i and j (Scheme 2)

(a) Amide coupling (intermediate i): Intermediate **h** (1 eq) was dissolved in DCM with 3-trifluoromethyl benzoic acid (1.3 eq) and 4-dimethylaminopyridine (DMAP, 0.1 eq). 1-Ethyl-3-(3-dimethylaminopropyl)carbodiimide hydrochloride (EDCI.HCl, 1.5 eq) was then added, and the reaction mixture was stirred at 25 °C for 16 hours. Water was added, and the solution was extracted with ethyl acetate, dried over anhydrous Na_2SO_4, and concentrated under reduced pressure. The residue was used in the subsequent reaction without any further purification.

(b) Cyclization (intermediate j): The amide intermediate **i** was dissolved in ethanol (10 mL), and 2 M NaOH solution (10 mL) was added. The resulting mixture was heated at 80 °C for 16 hours. When the reaction had gone to completion, the solvent was removed *in vacuo*, and saturated citric acid was added to the residue. Extraction was done with DCM (2 × 20 mL), and the combined organic extract was dried over anhydrous Na_2SO_4, filtered, and concentrated *in vacuo*. The residue was purified via column chromatography (DCM/MeOH) to obtain the desired product.

***tert*-Butyl (2-((1-(4-methoxybenzyl)-2-(3-(trifluoromethyl)phenyl)-1*H*-imidazo[4,5-c]pyridin-6-yl)amino)ethyl)carbamate (j).** Obtained from intermediate **h** (5.10 g, 13.16 mmols) and 3-trifluoromethyl benzoic acid (3.25 g, 17.11 mmols) as a wine-coloured sticky solid (65%, 4.63 g); R_f (DCM: MeOH, 9:1) 0.58; ^{1}H NMR (600 MHz, MeOH-d_4) δ 8.49 (s, 1H), 8.36 (ddd, J = 1.6, 1.5, 0.8 Hz, 1H), 8.27 (ddd, J = 7.8, 1.6, 1.3 Hz, 1H), 7.92 (s, 1H), 7.73 (d, J = 8.7 Hz, 2H), 7.60 (ddd, J = 8.6, 1.5, 1.3 Hz, 1H), 7.53 (ddd, J = 8.6, 7.8, 0.8 Hz, 1H), 6.99 (d, J = 8.7 Hz, 2H), 5.32 (s, 2H), 3.76 (s, 3H), 3.42–3.39 (m, 4H), 1.40 (s, 9H). ^{13}C NMR (151 MHz, MeOD) δ 170.61, 159.53, 134.48, 134.06, 132.87, 132.28, 129.89, 129.53, 128.59 (2C), 128.23, 127.75, 127.24, 126.86, 126.65, 126.21, 114.56 (2C), 86.37, 79.61, 55.85, 48.06, 45.50, 44.34, 43.44, 28.37 (3C). HPLC-MS (ESI): Purity = 96%, t_R = 2.650 min, m/z $[M+H]^+$ = 542.2.

Procedure for the synthesis of intermediate k (Scheme 2)

(a) Boc-deprotection: Intermediate **j** was dissolved in 4 M HCl/dioxane, and the mixture was stirred at 25 °C for 2 hours. When the reaction was complete, the solvent was removed *in vacuo,* and the residue was neutralized with Amberlyst A21 in a mixture of DCM and methanol. The Amberlyst was filtered off, the solvent was removed *in vacuo,* and the residue was used in the next reaction without further purification.

(b) Reductive amination: The crude product from step (a) above and 4-chloro-2-hydroxybenzaldehyde in methanol was stirred at 25 °C for 6 hours. The mixture was cooled at 0 °C, and sodium borohydride ($NaBH_4$) was added portion-wise. After the addition, the reaction was allowed to warm to room temperature (25 °C) for 3 hours. The solvent was removed *in vacuo,* and the residue was diluted with deionized water. The compound was extracted with DCM and dried over anhydrous sodium sulphate. The solvent was removed *in vacuo,* and the residue was purified via column chromatography to obtain the desired product.

5-chloro-2-(((2-((1-(4-methoxybenzyl)-2-(3-(trifluoromethyl)phenyl)-1*H*-imidazo[4,5-c]pyridin-6-yl)amino)ethyl)amino)methyl)phenol (k). Obtained from intermediate **j** (1.50 g, 3.49 mmols) and 3-trifluoromethyl benzoic acid (800 mg, 5.10 mmols) as a wine-coloured sticky solid (65%, 1.32 g); R_f (DCM: MeOH, 9:1) 0.60; ^{1}H NMR (600 MHz, MeOH-d_4) δ 8.47 (d, *J* = 1.0 Hz, 1H), 7.92 – 7.88 (m, 2H), 7.85 (d, *J* = 2.3 Hz, 1H), 7.71 (t, *J* = 7.8 Hz, 1H), 7.06 (d, *J* = 8.1 Hz, 1H), 6.95 (d, *J* = 8.7 Hz, 2H), 6.84 (d, *J* = 8.7 Hz, 2H), 6.74 (d, *J* = 2.1 Hz, 1H), 6.71 (dd, *J* = 8.0, 2.1 Hz, 1H), 6.47 (d, *J* = 1.0 Hz, 1H), 5.32 (s, 2H), 3.96 (s, 2H), 3.74 (s, 3H), 3.50 (t, *J* = 5.8 Hz, 2H), 2.96 (t, *J* = 5.8 Hz, 2H). ^{13}C NMR (151 MHz, MeOD) δ 159.51, 158.83, 155.74, 153.11, 144.35, 138.64, 133.98, 133.57, 132.42, 130.44, 130.29, 129.58, 127.65, 127.30 (2C), 126.70, 125.68, 124.66, 122.86, 121.00, 118.16, 115.57, 114.10 (2C), 86.40, 62.94, 58.97, 54.36, 48.88, 40.93. HPLC-MS (ESI): Purity = 96%, t_R = 2.466 min, m/z $[M+H]^+$ = 582.1.

Procedure for the synthesis of intermediate l (Scheme 2)

The intermediate **k** was stirred in neat TFA (10 mL) at 100 °C for 16 hours. Once the reaction was complete, TFA was removed under reduced pressure. The residue was dissolved in DCM/MeOH (9:1) and stirred with Amberlyst A21 for 1 hour. The resin was filtered off and

the filtrate concentrated under reduced pressure. The residue was purified via column chromatography to obtain the product.

5-chloro-2-(((2-((2-(3-(trifluoromethyl)phenyl)-1*H*-imidazo[4,5-c]pyridin-6-yl)amino)ethyl)amino)methyl)phenol (l). Obtained from intermediate **k** (1.00 g, 1.72 mmols) as a wine-coloured solid (75%, 596 mg); R_f (DCM: MeOH, 9:1) 0.18; ^{1}H NMR (600 MHz, MeOH-d_4) δ 8.40 (s, 1H), 8.36 (dd, *J* = 2.8, 2.2 Hz, 1H), 8.31 (dd, *J* = 7.8, 2.2 Hz, 1H), 7.83 (d, *J* = 2.2 Hz, 1H), 7.75 (ddd, *J* = 7.8, 2.8, 2.3 Hz, 1H), 7.27 (d, *J* = 7.8 Hz, 1H), 6.90 – 6.85 (m, 2H), 6.73 (s, 1H), 4.22 (s, 2H), 3.65 – 3.62 (m, 4H). ^{13}C NMR (151 MHz, MeOD) δ 156.96, 154.42, 153.25, 136.07, 132.34, 131.19, 130.11, 129.89, 128.88, 127.06, 126.60, 124.81, 123.36, 123.01, 119.59, 116.81, 115.04, 113.98, 89.42, 48.65, 46.31, 39.63. HPLC-MS (ESI): Purity = 98%, t_R = 2.413 min, m/z $[M+H]^+$ = 462.1.

Procedure for the synthesis of intermediate m (Scheme 2)

A mixture of intermediate **l**, N-boc glycinal and a catalytic amount of acetic acid in methanol was stirred at 25 °C for an hour. $NaBH_3CN$ was then added, and the resulting mixture was heated at 80 °C under reflux for 16 hours. When the reaction had completed, the methanol was removed *in vacuo* and the residue reconstituted in DCM. Saturated $NaHCO_3$ solution was then added to neutralize the acetic acid. The organic layer was washed with brine, dried over anhydrous Na_2SO_4 and concentrated *in vacuo*. The residue was purified via column chromatography to obtain intermediate **m**.

***tert*-Butyl(2-((4-chloro-2-hydroxybenzyl)(2-((2-(3-(trifluoromethyl)phenyl)-1H-imidazo[4,5-c]pyridin-6-yl)amino)ethyl)amino)ethyl)carbamate (m).** Obtained from intermediate **l** (370 mg, 0.80 mmols) and N-boc glycinal (254 mg, 1.60 mmols) as an off-white solid (75%, 339 mg); R_f (DCM: MeOH, 9:1) 0.56; ^{1}H NMR (600 MHz, MeOH-d_4) δ 8.38 (s, 1H, H^1), 8.37 (dd, *J* = 2.8, 2.2 Hz, 1H), 8.29 (dd, *J* = 7.8, 2.2 Hz, 1H), 7.80 (d, *J* = 2.2 Hz, 1H), 7.73 (ddd, *J* = 7.8, 2.8, 2.3 Hz, 1H), 7.01 (d, *J* = 7.8 Hz, 1H, H^{10}), 6.69 – 6.63 (m, 2H, H^4 and H^5), 6.53 (s, 1H, H^2), 3.78 (s, 2H, H^9), 3.45 (t, *J* = 6.3 Hz, 2H), 3.21 (t, *J* = 6.2 Hz, 2H), 2.82 (t, *J* = 6.2 Hz, 2H), 2.68 (t, *J* = 6.3 Hz, 2H), 1.38 (s, 9H). ^{13}C NMR (151 MHz, MeOD) δ 159.47, 157.87, 157.09, 155.59, 147.18, 138.23, 133.37, 131.33, 131.12, 130.37, 129.77, 128.72, 127.86, 126.57, 126.04, 124.87, 123.15, 121.86, 118.78, 115.49, 113.84, 93.10, 78.77,

55.73, 53.44, 39.82, 27.35 (3C). HPLC-MS (ESI): Purity = 98%, t_R = 2.562 min, m/z $[M+H]^+$ = 605.2.

Procedure for the synthesis of target compound 14-NBD (Scheme 2)

(a) Boc-deprotection: Intermediate **m** was dissolved in 4 M HCl/dioxane, and the mixture was stirred at 25 °C for 2 hours. When the reaction was complete, the solvent was removed *in vacuo,* and the residue was neutralized with Amberlyst A21 in a mixture of DCM and methanol. The Amberlyst was filtered off, the solvent was removed *in vacuo,* and the residue was used in the next reaction without further purification.

(b) Nucleophilic substitution: A mixture of the crude product from step (a) above, $NaHCO_3$ and NBD-Chloride in ethyl acetate, was stirred at 60 °C for 16 hours. The reaction was quenched with water and extracted with ethyl acetate. The organic layer was washed with brine, dried over anhydrous Na_2SO_4 and concentrated *in vacuo*. The residue was purified via column chromatography to obtain the fluorescent probe **14-NBD**.

5-chloro-2-(((2-((7-nitrobenzo[c][1,2,5]oxadiazol-4-yl)amino)ethyl)(2-((2-(3-(trifluoromethyl)phenyl)-1*H*-imidazo[4,5-c]pyridin-6-yl)amino)ethyl)amino)methyl)phenol (14-NBD). Obtained from intermediate **m** (339 mg, 0.56 mmols) and NBD-chloride (254 mg, 1.27 mmols) as a brick-red solid (50%, 187 mg); R_f (DCM: MeOH, 9:1) 0.56; ^{1}H NMR (600 MHz, DMSO-d_6) δ 8.39 (s, 1H), 8.37 (d, *J* = 8.8 Hz, 1H), 8.35 (dd, *J* = 2.8, 2.2 Hz, 1H), 8.31 (d, *J* = 8.8 Hz, 1H), 7.82 (dd, *J* = 7.8, 2.2 Hz, 1H), 7.74 (ddd, *J* = 7.8, 2.8, 2.3 Hz, 1H), 7.13 (dd, *J* = 8.4, 7.8 Hz, 1H), 6.85 (d, *J* = 7.8 Hz, 1H), 6.59 (ddd, *J* = 8.4, 2.3 Hz, 1H), 6.19 (d, *J* = 2.2 Hz, 1H), 6.04 (s, 1H), 3.70 (s, 2H), 3.53 (t, *J* = 6.3 Hz, 2H), 3.39 (t, *J* = 6.0 Hz, 2H), 2.82 – 2.76 (m, 4H). ^{13}C NMR (151 MHz, DMSO) δ 159.13, 158.92, 157.57, 155.85, 154.95, 149.82, 147.41, 143.12, 139.53, 138.17, 137.90, 135.58, 132.31, 131.80, 131.35, 129.01, 128.78, 126.30, 123.09, 118.92, 115.35, 99.50, 92.26, 86.51, 55.54, 53.70, 51.38, 44.39, 41.56, 36.89. HPLC-MS (ESI): Purity = 98%, t_R = 0.905 min, m/z $[M+H]^+$ = 668.2.

Biological Evaluation

In vitro Asexual Blood Stage Antiplasmodium Assay

Parasites were cultured as per the method of Trager and Jensen,[1] with modifications. IC_{50} values were determined using parasite lactate dehydrogenase as a marker for survival (Makler

et al, 1993). The test samples were prepared to a 10 mmol/L stock solution in 100% DMSO. Samples were tested as a suspension if not completely dissolved. Further dilutions to the desired starting concentration were freshly prepared in growth media on each occasion of the experiment. The standard antimalarial drugs chloroquine (CQ) and artesunate (Arts) were used as the reference drug in all experiments. A full dose-response was performed for all compounds in a 96-well plate to determine the concentration inhibiting 50% of parasite growth (IC_{50}–value). Test samples were tested at a starting concentration of 6mmol/L, which was then serially diluted 2-fold in growth medium to generate the tested concentration range. The same dilution technique was used for all samples. CQ and Arts were tested from a starting concentration of 1µg/mL. The highest concentration of solvent to which the parasites were exposed was <0.1% and has no measurable effect on the parasite viability (data not shown).

The assay plate was incubated at 37 °C for 72h in a sealed gas chamber under 3% O_2 and 4% CO_2 with the balance being N_2. After 72h, the wells in the assay plate were gently resuspended, and 15µL from each well was transferred to a duplicate plate containing 100µL of Malstat reagent and 25µL of nitroblue tetrazolium solution in each well. Plates were left to develop for 20 minutes in the dark and then absorbance of each well was quantified using a spectrophotometer at 620nM wavelength.

The remaining population of parasites at each concentration of the test compound was determined by comparing the absorbance of each well to the absorbance of a well containing the drug-free control. Survival was plotted against concentration and the IC_{50} values were obtained using a non-linear dose-response curve fitting analysis via the Dotmatics software platform.[2]

***In vitro* Cytotoxicity Assay**

In vitro cytotoxicity was performed on the Chinese Hamster Ovarian cell line by measuring cellular growth and survival calorimetrically through the 3-(4,5-dimethylthiazol-2-yl)-2,5-diphenyltetrazolium bromide (MTT) assay.[3,4] The formation of tetrazolium salt was used as a measure of chemosensitivity and growth.

2 mg/mL stock solutions of the test compounds were prepared in DMSO and stored at -20 °C until required. The stock solution was subsequently diluted with the assay medium to make up an initial concentration of 100 µg/mL. The initial 100 µg/mL solution is serially diluted 10-

fold to attain six assay concentrations of 100-0.001 µg/mL. Emetine was used as the reference compound in this assay. The highest concentration of DMSO, which was used as the negative control, had no measurable effect on cell viability. After 44 hours of exposure of the cells to the test compounds, a solution of MTT was added. This was followed by an additional 4 hours of incubation at 37 °C. The assay plates were centrifuged, medium removed, and DMSO was added to the crystals. The amount of formazan was ascertained by measuring the absorbance at 540 nm. GraphPad Prism software was then used to generate IC_{50} values through a non-linear dose-response curve analysis.

β-Hematin Inhibition Assay

20 mM stock solutions of controls and test compounds were made in DMSO. A solution containing water, 305.5 µM NP-40 and DMSO at a v/v ratio of 70%:20%:10%, respectively, was added to every well in columns 1-11 of a 96-well plate. 140 µL of water and 40 µL of 305.5 µM NP-40 were added to column 12 to mediate the formation of beta-hematin. 20 µL of test compounds (20 mM) were added to column 12 and 100 µL of this solution serially diluted to column 2, leaving column 1 blank (0 µM of test compound). 178.8 µL aliquot of hematin stock was suspended in 20 ml of a 1 M acetate buffer at pH 4.9. 100 µL of this hematin suspension was then added into each well, and the plates were incubated for ±5 hrs at 37°C. Subsequently, 32 µL of pyridine solution (20% water, 20% acetone, 10% 2M HEPES buffer at pH 7.4 and 50% pyridine) and 60 µL of acetone were added to all wells. The absorbances of plate wells were recorded at 405 nm on a SpectraMax plate reader. Sigmoidal dose-response curves with variable slopes were fitted to the absorbance data using GraphPad Prism to obtain the IC_{50} values for each compound.

In vitro Microsomal Stability Assay

The *in vitro* microsomal stability assay was performed in duplicate in a 96-well micro titre plate, using a single-point experiment design.[5] The test compounds (1 µM) were incubated individually in human (pool of 50, mixed gender), rat (pool of 711, male Sprague Dawley) and mouse (pool of 1634, male CD1) liver microsomes (final protein concentration of 0.4 mg/mL; Xenotech, Kansas, USA), suspended in 0.1M phosphate buffer (pH 7.4). Incubations were

started by addition of NADPH (1 mM) as cofactor and shaken for 30 minutes at 37ºC. The reactions were then quenched by adding 300 µL of ice-cold acetonitrile containing internal standard (carbamazepine, 0.0236 µg/mL) and centrifuged. The supernatant was transferred to a fresh 96-well plate and analysed by LC-MS/MS (Agilent Rapid Resolution HPLC, AB SCIEX 4500 MS). The relative loss of parent compound over time was monitored and plots (concentration vs. time) were prepared per compound to determine the first order rate constant for compound depletion. This was in turn used to calculate half-life, *in vitro* intrinsic clearance and *in vivo* hepatic extraction ratio.[6]

Metabolite Identification Studies

The test compound (10 µM) was incubated at 37 °C in a solution containing 1 mg/ml microsomes (MLM, male mouse CD1, Lot No. 1510043, Xenotech), magnesium chloride (5 mM) and NADPH (1 mM) in sodium phosphate buffer (100 mM, pH 7.4) for 60 minutes while shaking. The samples were then prepared by ice-cold acetonitrile precipitation, centrifuged, and filtered for LC-MS/MS analysis.

Controls containing all the sample constituents (not incubated), and in which NADPH, microsomes or the test compound were individually excluded were also prepared and handled similarly to the test sample. Verapamil (10 µM) was incubated concomitantly as a positive control.

Fluorescence Live-cell Microscopy

General Methods

Nunc Lab-Tek II eight-well chamber slides (Thermo Fisher Scientific, Massachusetts, USA) with a cover glass of No. 1.5 thickness were coated with 150 µL of 0.01% (w/v) poly-L-lysine solution. After ten minutes, the solution was removed, and the plates were left to air-dry. Ringer's solution was prepared according to the compositions outlined in Table S2, as previously described.[7] The pH was adjusted to 7.4, after which the solution was filtered through a 0.22 µm nylon syringe filter.

Table S1: **Constituents of Ringer's solution for live-cell imaging of the *P. falciparum*.**

Component	Molecular weight	Final Concentration	Mass required for 100mL
**NaCl**	58.44	122.5 mM	715.9 mg
**KCl**	74.56	5.4 mM	40.3 mg
**$CaCl_2$**	147.02	1.2 mM	17.6 mg
**$MgCl_2$**	95.21	0.8 mM	7.6 mg
**D-glucose**	180.16	11 mM	198.2 mg
**Hepes**	238.30	25 mM	595.8 mg
**NaH_2PO_4**	119.98	1 mM	12.0 mg

Human erythrocytes infected with *P. falciparum* trophozoites (NF54 strain) were generously donated by colleagues at the Division of Pharmacology, Department of Medicine, University of Cape Town. After harvesting and centrifugation of the cells, 5 μL of the parasitized erythrocyte pellets were resuspended in 5 mL Ringer's solution and vortexed to reduce clumping of the erythrocytes. 150 μL aliquots of suspended cells were placed in each well of the chamber slide and incubated for at least 20 minutes to allow the erythrocytes to adhere to the cover glass. After this, the Ringer's solution was removed and replaced with a fresh aliquot of Ringer's solution (150 μL) to remove non-adhered cells. This solution was then removed again and replaced with Ringer's solution (150 μL) containing appropriate concentration(s) of the fluorescent dye(s) as listed in Table S3.

Live-cell Confocal Microscopy

Confocal microscopy was performed using a Zeiss Axiovert 200 M LSM 150-META confocal microscope at the Confocal and Light Microscope Imaging Facility at the University of Cape Town. A Plan-Apochromat 63x/1.40 Oil DIC M27 objective lens was used, and the cells were incubated at 37°C. Images were captured and processed with ZEN 2018 (Carl Zeiss MicroscopyGmbH). Laser transmission was kept as low as possible to minimize phototoxicity to the cells.[8] Dye concentrations with respective excitation and emission settings are listed in Table S3.

Table S2: Concentrations of fluorescent dyes used for confocal imaging of *P. falciparum* with respective excitation lasers and emission filter settings.

Dye	Concentration	Excitation Laser	Emission filter
**DRAQ 5**	500 nM	580 nm	600-630 nm
**LysoTracker**	100 nM	561 nm	575-630 nm
**Nile Red**	100 nM	561 nm	575-630 nm
**ER-Tracker**	100 nM	561 nm	575-630 nm
**MitoTracker**	100 nM	561 nm	650-710 nm
**1.3-NBD**	50 nM	420 nm	500-550 nm
**3.14-NBD**	75 nM	420 nm	500-550 nm

Heme Fractionation Assay

Preparation of Counting Plate and Cell Fixation

The heme fractionation assay was performed according to the methods previously described.[9] After the IC_{50} of the target compounds was determined, a counting plate was prepared by adding 10 µL of the isolated washed trophozoites to the corresponding wells of a flat-bottomed 96-well plate. The cells were fixed with 0.125 % (v/v) glutaraldehyde in PBS pH 7.5 to a final volume of 200 µL and refrigerated at 4 °C overnight.

Hemocytometer Counting

Hemocytometer counting was performed on the first row of each plate only, corresponding to six samples, each at a different concentration of the drug tested. The hemocytometer determined cell counts for these six samples were statistically compared to the cell counts determined by flow cytometry to test for agreement. 10 µL of cells from the counting plate were loaded onto a bright-lined hemocytometer, and five large squares were counted after 10 minutes of settling. The concentration of cells in each well of the plate was determined using equation 1:

CH ¼ N x F x DF x 10,000 **(1)**

Where: CH = concentration of cells per ml as determined with hemocytometer

N = number of cells counted in five fields

F = number of fields counted = 5

DF = dilution factor

Flow Cytometry Counting

Cell counts for all samples on the counting plate were determined using flow cytometry. Samples were analyzed on a Becton Dickinson FACSCalibur using SSC/$FL1_{530nm}$ with CellQuestPro software. Typically, 10,000 events were counted for each sample. Samples were prepared by diluting 100 µL of cells from the counting plate with 800 µL of 1 × SYBR® green I in PBS at pH 7.5 and incubated for 30 minutes in the dark at 37 °C. Next, each sample was spiked with 100 µL of Trucount™ beads (Becton Dickinson) such that each sample contained a known fixed amount of fluorescent beads in a final volume of 1 mL. The concentration of cells in the acquisition tube was calculated according to equation 2:

CF= (T/B) x CB **(2)**

Where:

CF = concentration of cells per ml as determined with flow cytometry

T = number of trophozoites gated

B = number of fluorescent beads gated

CB = concentration of fluorescent beads in the acquisition tube per ml (calibrated bead count per acquisition is unique to each lot of tubes obtained from the supplier).

Heme Fractionation

After thawing the stock plate, 50 µL of water was added, and the plate was sonicated for 5 min in an ultrasound bath (53 kHz, 320 W, Bandelin Sonerex). This was followed by adding 50 µL each of 0.2 M HEPES buffer at pH 7.5 and water. The plate was then centrifuged at 3600 rpm for 20 minutes. Very carefully, without disturbing the pellet, the supernatant was transferred to an adjacent set of wells on the same plate. The supernatant was processed further with 50 µL of 4% SDS, sonicated for 5 minutes and then incubated at 37 °C for 30 minutes. 50 µL of 0.3 M NaCl and 50 µL of 25% (v/v) pyridine in 0.2 M HEPES buffer at pH 7.5 were added, and 200 µL of the solution was transferred to a flat-bottomed UV-Star 96-well plate. This fraction corresponds to the hemoglobin fraction.

The pellet was treated with 50 µL water, 50 µL of 4% SDS and resuspended. The plate was sonicated for 5 minutes and incubated at 37 °C for 30 minutes to solubilize free heme. This was followed by adding 50 µL 0.2 M HEPES buffer at pH 7.5, 50 µL 0.3 M NaCl and 50 µL of 25% pyridine. The plate was centrifuged at 3700 rpm for 20 minutes. Very carefully, without disturbing the pellet, the supernatant was transferred to an adjacent set of wells on the same

plate. The supernatant was diluted to a final volume of 400 µL with water. This fraction corresponds to the free heme fraction. 200 µL of this solution was transferred to the flat-bottomed UV-Star 96-well plate, the same plate previously used for the hemoglobin fraction. The remaining pellet containing hemozoin was solubilized in 50 µL of water and 50 µL 0.3 M NaOH. The plate was sonicated for 15 minutes and incubated at 37 °C for 30 minutes. 50 µL each of 0.2 M HEPES buffer at pH 7.5, 0.3 M HCl and 25 % pyridine was added, followed by 150 µL of water. This fraction corresponds to the hemozoin fraction. 200 µL of this solution was transferred to vacant wells in the flat-bottomed UV-Star 96-well plate containing the hemoglobin and free heme fractions. The UV-visible spectra of heme as Fe(III)heme-pyridine complex were recorded using a multi-well plate reader (SpectraMax 340PC, Molecular Devices). The absorbance maxima of the Fe(III)heme-pyridine complex in each well was used to calculate the percentage of heme species in each sample as the final volume for each fraction was identical.

Heme Curve

The total amount of heme in each fraction was quantified using a standard heme curve prepared from a 100 µg/mL standard heme solution of hematin (porcine) in 0.3 M NaOH. Serial dilutions of the standard were carried out in a 96-well plate with 100 µL 0.3 M NaOH as a blank. 50 µL of each of the following solutions were added to 100 µL of hematin standard: 0.2 M HEPES buffer pH 7.5, 4% (w/v) SDS, 0.3 M NaCl, 0.3 M HCl, 25% pyridine in 0.2 M HEPES buffer pH 7.5 and water. The visible spectra of heme as Fe(III)heme-pyridine complex were recorded in a multi-well plate reader. The amount of heme Fe per cell was calculated by dividing the total heme Fe in each fraction by the number of cells determined in each fraction. Chloroquine and pyrimethamine were used as positive and negative controls respectively.

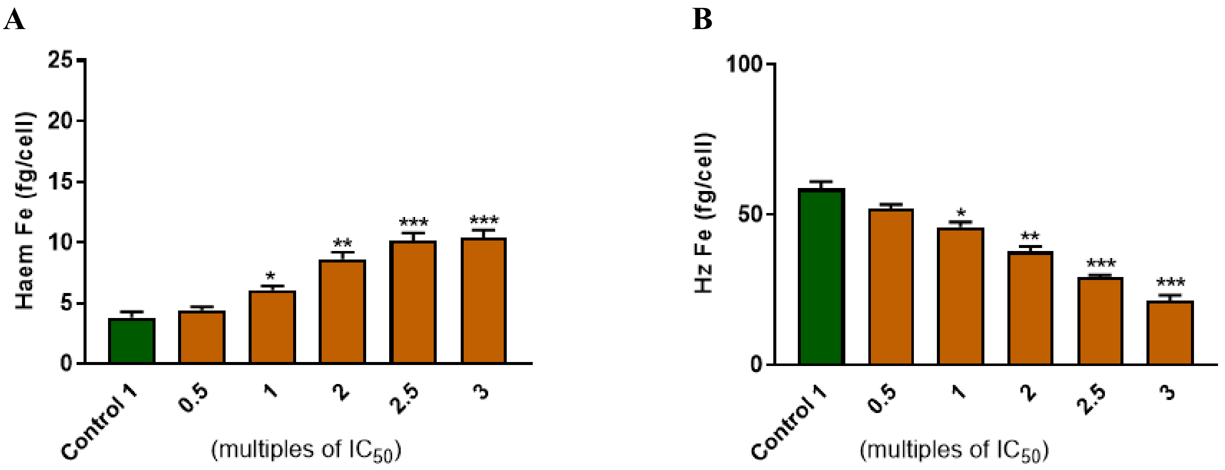

Figure S1: Dose-dependent heme fractionation profiles of compound **CQ** The amount of "free" heme Fe (**A**) and hemozoin (Hz) Fe (**B**) at increasing concentrations of the compounds.

Docking Studies

Compounds were docked against the β-hematin surface using the previously publishes 3D crystal structure.[10] The Fe-O bonds were treated as zero order bonds to constrain the input geometry. The receptor grid encompassed the (100) and (001) faces of the crystal. The protonation states of both the crystal and the ligands were generated at pH 4.8 ± 0.5 using Epik. The ligands were minimized using Schrödinger's OPLSe force field. The output conformers were docked using Grid-based ligand docking with energetics (Glide extra precision) Epik states penalties were added to the docking score, and intramolecular hydrogen bonds were rewarded. The planarity of conjugated π groups was also enhanced.

References

(1) Trager, W.; Jensen, J. Human Malaria Parasites in Continuous Culture. *Science (80-.).* **1976**, *193* (4254), 673–675. https://doi.org/10.1126/science.781840.

(2) Piper, R. C.; Williams, J. A.; Makler, M. T.; Gibbins, B. L.; Hinrichs, D. J.; Ries, J. M.; Bancroft, J. E. Parasite Lactate Dehydrogenase as an Assay for Plasmodium Falciparum Drug Sensitivity. *Am. J. Trop. Med. Hyg.* **1993**, *48* (6), 739–741. https://doi.org/10.4269/ajtmh.1993.48.739.

(3) Mosmann, T. Rapid Colorimetric Assay for Cellular Growth and Survival: Application to Proliferation and Cytotoxicity Assays. *J. Immunol. Methods* **1983**, *65* (1–2), 55–63. https://doi.org/10.1016/0022-1759(83)90303-4.

(4) van Meerloo, J.; Kaspers, G. J. L.; Cloos, J. Cell Sensitivity Assays: The MTT Assay; 2011; pp 237–245. https://doi.org/10.1007/978-1-61779-080-5_20.

(5) Di, L.; Kerns, E. H.; Gao, N.; Li, S. Q.; Huang, Y.; Bourassa, J. L.; Huryn, D. M. Experimental Design on Single-Time-Point High-Throughput Microsomal Stability Assay. *J. Pharm. Sci.* **2004**, *93* (6), 1537–1544. https://doi.org/10.1002/jps.20076.

(6) Obach, R. S. Prediction of Human Clearance of Twenty-Nine Drugs from Hepatic Microsomal Intrinsic Clearance Data: An Examination of in Vitro Half-Life Approach and Nonspecific Binding to Microsomes. *Drug Metab. Dispos.* **1999**, *27* (11), 1350–1359.

(7) Pisciotta, J. M.; Coppens, I.; Tripathi, A. K.; Scholl, P. F.; Shuman, J.; Bajad, S.; Shulaev, V.; Sullivan, D. J. The Role of Neutral Lipid Nanospheres in Plasmodium Falciparum Haem Crystallization. *Biochem. J.* **2007**, *402* (1), 197–204. https://doi.org/10.1042/BJ20060986.

(8) Wissing, F.; Sanchez, C. P.; Rohrbach, P.; Ricken, S.; Lanzer, M. Illumination of the Malaria Parasite Plasmodium Falciparum Alters Intracellular PH. *J. Biol. Chem.* **2002**, *277* (40), 37747–37755. https://doi.org/10.1074/jbc.M204845200.

(9) Combrinck, J. M.; Fong, K. Y.; Gibhard, L.; Smith, P. J.; Wright, D. W.; Egan, T. J. Optimization of a Multi-Well Colorimetric Assay to Determine Haem Species in Plasmodium Falciparum in the Presence of Anti-Malarials. *Malar. J.* **2015**, *14* (1), 253. https://doi.org/10.1186/s12936-015-0729-9.

(10) Pagola, S.; Stephens, P. W.; Bohle, D. S.; Kosar, A. D.; Madsen, S. K. The Structure of Malaria Pigment β-Haematin. *Nature* **2000**, *404* (6775), 307–310. https://doi.org/10.1038/35005132.
